# An antifouling membrane-fusogenic liposome for effective intracellular delivery in vivo

**DOI:** 10.1038/s41467-024-46533-z

**Published:** 2024-05-20

**Authors:** Huimin Kong, Chunxiong Zheng, Ke Yi, Rachel L. Mintz, Yeh-Hsing Lao, Yu Tao, Mingqiang Li

**Affiliations:** 1 Laboratory of Biomaterials and Translational Medicine, Center for Nanomedicine, The Third Affiliated Hospital, Sun Yat-sen University, Guangzhou, 510630 China; 2 School of Chemistry, South China Normal University, Guangzhou, 510006 China; 3 Department of Biomedical Engineering, Washington University in St. Louis, St. Louis, MO 63110 USA; 4 Department of Pharmaceutical Sciences, University at Buffalo, The State University of New York, Buffalo, NY 14214 USA

**Keywords:** Drug delivery, Nanoparticles, Transfection

## Abstract

The membrane-fusion-based internalization without lysosomal entrapment is advantageous for intracellular delivery over endocytosis. However, protein corona formed on the membrane-fusogenic liposome surface converts its membrane-fusion performance to lysosome-dependent endocytosis, causing poorer delivery efficiency in biological conditions. Herein, we develop an antifouling membrane-fusogenic liposome for effective intracellular delivery in vivo. Leveraging specific lipid composition at an optimized ratio, such antifouling membrane-fusogenic liposome facilitates fusion capacity even in protein-rich conditions, attributed to the copious zwitterionic phosphorylcholine groups for protein-adsorption resistance. Consequently, the antifouling membrane-fusogenic liposome demonstrates robust membrane-fusion-mediated delivery in the medium with up to 38% fetal bovine serum, outclassing two traditional membrane-fusogenic liposomes effective at 4% and 6% concentrations. When injected into mice, antifouling membrane-fusogenic liposomes can keep their membrane-fusion-transportation behaviors, thereby achieving efficient luciferase transfection and enhancing gene-editing-mediated viral inhibition. This study provides a promising tool for effective intracellular delivery under complex physiological environments, enlightening future nanomedicine design.

## Abstract (web summary)

Protein corona formation on the surface of liposome nanocarriers can decrease the efficiency of intracellular delivery. Here, the authors develop antifouling membrane-fusogenic liposomes for effective intracellular delivery in vivo.

## Introduction

Nanoparticle-based intracellular delivery remains challenging for biopharmaceutical and biomedical advancements due to the intractable lysosomal degradation or clearance after cellular endocytosis^1–4^. To address this limitation, a promising membrane fusion-based delivery strategy was developed recently^5–15^, which is usually achieved using a membrane-fusogenic liposome (MFlip) formulated by a specific lipid composition with an optimized ratio^5,9,15–18^. This MFlip can implement a biomimetic fusion process (i.e., close membrane contact, lipid blending, and inner content transferring) to bypass lysosomal entrapment for direct intracellular cargo transportation^13,19,20^. However, its delivery efficiency is usually dissatisfactory in protein-enriched biological environments after systemic administration^21^. This is because MFlip will inevitably interact with serum proteins to form thick protein corona on the surface, hindering the close contact of surface fusogenic lipids with cell membranes to interfere with the membrane-fusion process^22–24^. As a result, the membrane-fusion-based cell-internalization process of MFlips is prone to convert to endocytosis in the presence of high-concentration proteins, leading to lysosomal entrapment and reduced delivery efficiency^18,25,26^. Hence, we propose that inhibition of protein corona formation on the MFlip surface can maintain its membrane-fusion effect under protein-enriched conditions to enhance the efficiency and practicability of in vivo intercellular delivery.

Zwitterionic materials, with pairs of oppositely-charged groups coexisting on one molecule, have been reported with excellent resistance to the non-specific adsorption of biocomponents^27–29^. Due to the electrostatically-induced aquation, a strong hydration layer could be formed on the zwitterionic material surface, thereby alleviating protein adsorption to obtain an antifouling performance^30^. Based on this protein-resistance capacity, we optimistically assume that introducing an abundant ratio of zwitterionic groups onto the surfaces of MFlips can preserve their membrane-fusion efficiency in the protein-enriched biological environment by forming a non-fouling surface with reduced protein adsorption^27,28,30–33^. While some MFlips have utilized zwitterionic lipids like DMPC and DOPE, their membrane-fusion performance in the protein environment has often been subpar, compromised by the concurrently-used cationic lipids^15,18,21,34^.

**Fig. 1:**
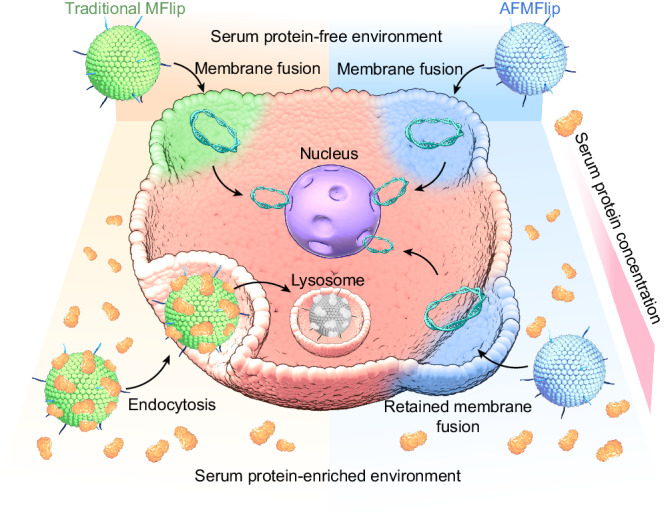
Schematic illustration of membrane fusion effects induced by MFlips. This diagram compares the cellular internalization behaviors of traditional MFlips with our antifouling MFlips (AFMFlips), highlighting differences in their interactions with serum proteins.

Herein, we rationally developed an antifouling MFlip (AFMFlip) with a strong protein-resistance capacity to achieve stable membrane fusion under serum-enriched conditions for efficient in vivo intracellular delivery (Fig. 1). Such AFMFlip was constructed by three lipid composites, including zwitterionic group-capped 1,2-dioleoyl-*sn*-glycero-3-phosphocholine (DOPC), 1,2-dioleoyloxy-3-(trimethylammonium) propane (DOTAP), and 1,2-distearoyl-*sn*-glycero-3-phosphoethanolamine-*N*-methoxy(polyethylene glycol) (DSPE-PEG), with an optimized molar ratio of 90/5/5. With the relatively higher portion of zwitterionic phosphorylcholine groups and a relatively less cationic DOTAP presenting on the AFMFlip surface, a much lower level of protein absorption was observed on its surface compared with the previously reported traditional MFlips (denoted as MFlip-a and MFlip-b) after serum exposure^5,9,17^. Owing to this excellent protein-adsorption-resistance ability, AFMFlip could achieve robust membrane-fusion-mediated cellular uptake and gene transfection in the medium containing up to 38% fetal bovine serum (FBS), as the critical concentration, far exceeding MFlip-a and MFlip-b (only up to 4% and 6% FBS, respectively). Furthermore, after intravenous injection into mice, AFMFlips still retained their membrane-fusion-mediated transport behavior in vivo, further accomplishing a more efficient luciferase transfection in the liver and an enhanced treatment of hepatitis B virus (HBV) infection via CRISPR/Cas9 gene editing. Overall, this AFMFlip presents a pioneering type of MFlips that remarkably maintains membrane-fusion effectiveness even in serum protein-enriched environments, offering an efficient nanocarrier for in vivo intracellular delivery of therapeutics.

## Results

### Preparation and optimization of AFMFlips

**Fig. 2:**
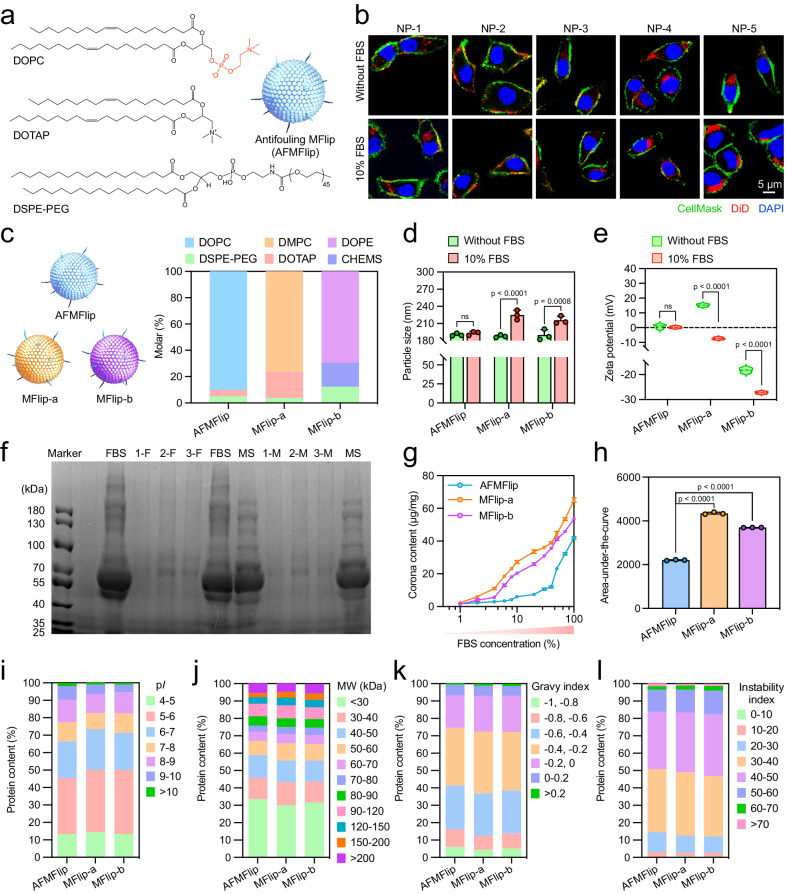
Preparation, optimization, and anti-protein-adsorption effect of AFMFlips. **a**, **b** Preparation and optimization of AFMFlips with serum-resistance membrane-fusion capacity. Liposomes were labeled with 1,1’-dioctadecyl-3,3,3’,3’-tetramethylindodicarbocyanine,4-chlorobenzenesulfonate salt (DiD). The nuclei were stained with DAPI and the cell membranes were labeled with CellMask. The experiment was repeated three times independently with similar results. **c** Composite comparison of AFMFlip and the previously reported MFlips (MFlip-a and MFlip-b). **d**, **e** Hydrodynamic sizes (**d**) and zeta potentials (**e**) of AFMFlip, MFlip-a, and MFlip-b after 1-h incubation in the medium without or with 10% FBS at 37 °C. Data are presented as mean ± SD and statistically analyzed using two-tailed *t* test (*n* = 3 biologically independent samples). ns: no significance (*p* > 0.05). Measurements were conducted in water at room temperature. **f** Sodium dodecyl sulfate-polyacrylamide gel electrophoresis (SDS-PAGE) analysis of corona proteins on AFMFlip, MFlip-a, and MFlip-b after 1-h incubation in the medium with 10% FBS or 10% mouse serum. FBS, fetal bovine serum; 1-F, AFMFlip incubated with 10% FBS; 2-F, MFlip-a incubated with 10% FBS; 3-F, MFlip-b incubated with 10% FBS; MS, mouse serum; 1-M, AFMFlip incubated with 10% mouse serum; 2-M, MFlip-a incubated with 10% mouse serum; 3-M, MFlip-b incubated with 10% mouse serum. The experiment was repeated three times independently with similar results. **g** The bicinchoninic acid (BCA) quantification of protein corona from AFMFlip, MFlip-a, and MFlip-b after 1-h FBS incubation with different concentrations. Data are presented as mean ± SD (*n* = 3 biologically independent samples). **h** The area under the curve (AUC) analysis based on the BCA-quantification results from **g**. Data are presented as mean ± SD and statistically analyzed using one-way ANOVA (*n* = 3 biologically independent samples). **i**–**l** Protein classification for corona proteins recovered from AFMFlip, MFlip-a, and MFlip-b after 1-h incubation in 10% FBS-containing medium by liquid chromatography with tandem mass spectrometry (LC-MS/MS) analysis. The identified proteins from corona were classified based on isoelectric point (pI) (**i**), molecular weight (MW) (**j**), gravy index (**k**), and instability index (**l)**. Source Data are provided as a Source Data file.

We formulated a series of MFlips (NP-1 to NP-5) containing three composites: zwitterionic phosphorylcholine-ending DOPC, cationic DOTAP, and DSPE-PEG, with varied molar ratios (Fig. 2a). Introducing phosphorylcholine on MFlip surfaces was hypothesized to balance its membrane-fusion transport and protein-resistance capacities. These liposomes with varying molar ratios of DOPC, DOTAP, and DSPE-PEG showed similar hydrodynamic sizes of ~200 nm and favorable polymer dispersity indexes (PDI) below 0.25. Concurrently, the zeta potentials increased progressively from −7.5 mV to 19.2 mV with the increasing ratios of the positively-charged DOTAP (Supplementary Table 1). These MFlips were labeled with 1,1’-dioctadecyl-3,3,3’,3’-tetramethylindodicarbocyanine,4-chlorobenzenesulfonate salt (DiD) and introduced to HeLa cells in the culture medium without or with 10% FBS, followed by confocal laser scanning microscopy (CLSM) imaging. As shown in Fig. 2b and Supplementary Fig. 1a, cells treated with NP-2 in FBS-free medium showed that most of DiD (red) signals were uniformly distributed on the CellMask-labeled cell membrane (green) to yield yellow overlaps, suggesting the potential uptake via membrane fusion. More importantly, when exposed to the 10% FBS-containing medium, only cells treated with NP-2 were observed with the red-green-colocalized fluorescence patterns. In contrast, cells treated with other liposomes showed either dotted or spread red fluorescence signals throughout. To further substantiate these observations, the liposomes were labeled with both 3,3’-dioctadecyloxacarbocyanine perchlorate (DiO) and 1,1’-dioctadecyl-3,3,3’,3’-tetramethylindocarbocyanine perchlorate (DiI), followed by incubation with HeLa cells. The membrane-fusion efficiency was then quantified using a fluorescence resonance energy transfer (FRET)-based assay (Supplementary Fig. 1b)^35,36^. The results indicated that NP-2 achieved the highest membrane fusion efficiency in both 10% FBS-free and -containing media. Thus, NP-2, with a molar ratio of 90/5/5 (DOPC/DOTAP/DSPE-PEG), a diameter of 190.5 nm, and a slightly positive charge (1.2 mV) (Supplementary Fig. 2), has the potential to retain the fusion behavior in the protein-containing condition.

### Protein-adsorption resistance of AFMFlips

The maintenance of membrane fusion in protein-containing conditions should be attributed to the decreased protein absorption on the surface due to both the zwitterionic DOPC introduction and reduced reliance on cationic DOTAP usage. To investigate the protein-resistance properties, two previously reported MFlips, MFlip-a with a strongly positive surface charge and MFlip-b without antifouling composites, were used for comparisons (Fig. 2c)^9,35^. Primarily, the physical characterizations of these MFlips were determined after 1-h incubation without or with 10% FBS at 37 °C. The exposure to FBS caused negligible changes in particle size and zeta potential of AFMFlip (Fig. 2d, e). However, this treatment remarkably increased the particle size of MFlip-a from 187.7 nm to 224.8 nm and MFlip-b from 189.7 nm to 215.7 nm (Fig. 2d). Meanwhile, the zeta potential of MFlip-a also dramatically decreased from +15.2 mV to −7.4 mV, and the zeta potential of MFlip-b decreased from −18.3 mV to −27.2 mV after FBS incubation (Fig. 2e). Hence, the physical properties of AFMFlips can be well maintained in serum protein-containing conditions, probably due to protein corona inhibition.

To directly verify the protein corona inhibition, MFlips were isolated after incubation in 10% FBS or mouse serum for semiquantitative sodium dodecyl sulfate-polyacrylamide gel electrophoresis (SDS-PAGE) analysis and quantitative bicinchoninic acid (BCA)-based assay. SDS-PAGE images in Fig. 2f exhibited more indistinct protein bands in the lane of AFMFlip after incubation with FBS and mouse serum compared with MFlip-a and MFlip-b, coinciding with the results of BCA protein quantitation (Supplementary Fig. 3). More importantly, this relatively-low protein absorption of AFMFlip is independent of the protein concentration, confirming the preferable antifouling capacity of our AFMFlip compared to conventional MFlip-a and MFlip-b (Fig. 2g h).

Next, the obtained protein corona compositions from AFMFlip, MFlip-a, and MFlip-b after incubation with 10% FBS were identified by liquid chromatography with tandem mass spectrometry (LC-MS/MS) analysis (Supplementary Fig. 4). After FBS treatment, the total relative raw abundance of proteins adsorbed by AFMFlip was 61.1% and 57.0% lower than that of MFlip-a and MFlip-b, respectively, which was highly accordant with the above SDS-PAGE and BCA results (Fig. 2f, Supplementary Fig. 3, and Supplementary Fig. 5). Supplementary Figs. 6–8 and Supplementary Table 2 described the twenty most abundant corona proteins of AFMFlip, MFlip-a, and MFlip-b after incubation in FBS. To further characterize the overall protein property of the corona compositions, proteins were analyzed according to the diverse parameters, including isoelectric point (pI), molecular weight (MW), gravy index, and instability index (Fig. 2i–l). Results revealed proteins with low pI (<7 at physiological pH) were predominantly enriched on the surface of each nanoparticle (Fig. 2i and Supplementary Fig. 9), consistent with previous studies^37^. Besides, the proteins with intermediate MW (30-90 kDa) occupied the largest fraction (Fig. 2j and Supplementary Fig. 10). Moreover, the tendency of a small gravy index and unstable index of all the samples indicated that most of the hydrophilic and stable proteins were absorbed on the surface (Fig. 2k, l).

### Cellular internalization pathway of AFMFlips in protein-containing medium

**Fig. 3:**
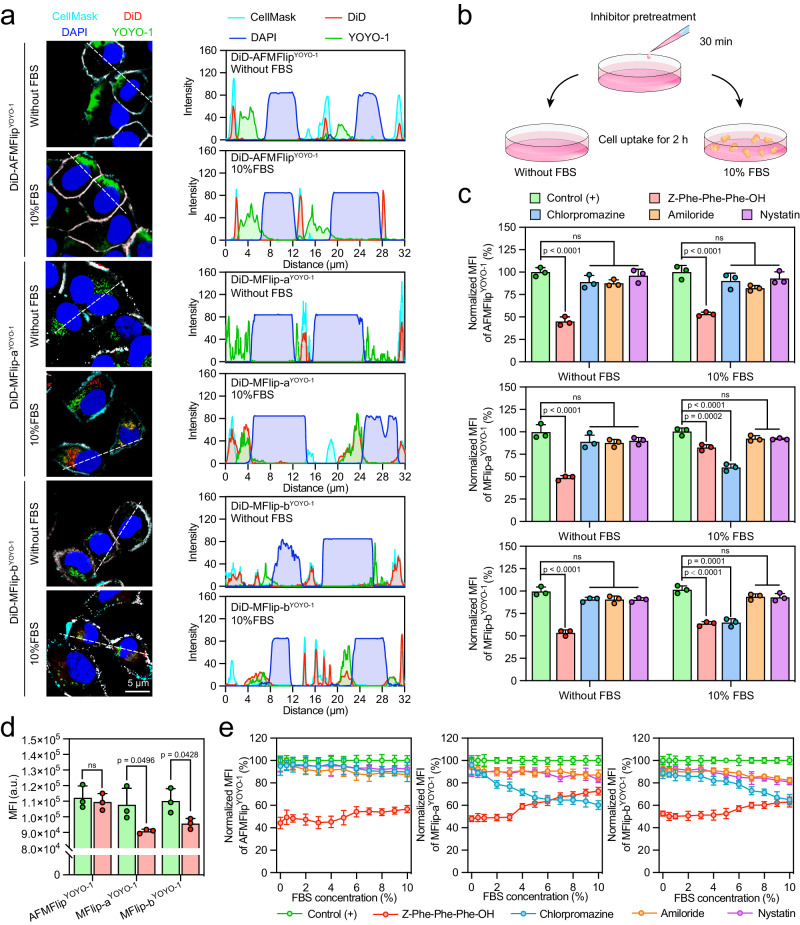
Cellular internalization of AFMFlips in the protein-enriched environment. **a** Confocal laser scanning microscopy (CLSM) imaging and corresponding line scanning analysis to investigate the cellular uptake of liposomes in the medium without or with 10% FBS. Liposomes were labeled with DiD. The nuclei were stained with DAPI, and the cell membranes were labeled with CellMask. Colocalization profiles are shown on the right side and were analyzed along the white lines by ImageJ software. The experiment was repeated three times independently with similar results. **b** Scheme of the mechanism investigation under the different endocytosis or membrane-fusion inhibitor treatment in the absence or presence of FBS. **c** Fluorescence-activated cell sorting (FACS) analysis of HeLa cells after treatment with AFMFlip^YOYO-1^, MFlip-a^YOYO-1^, and MFlip-b^YOYO-1^ for 2 h in the FBS-free and 10% FBS-containing medium. Cells were pretreated with the membrane-fusion (Z-Phe-Phe-Phe-OH) or endocytosis (chlorpromazine, amiloride, and nystatin) inhibitors at designed concentrations for 30 min at 37 °C. Data are presented as mean ± SD and statistically analyzed using one-way ANOVA (*n* = 3 biologically independent samples). ns: no significance (*p* > 0.05). **d** Mean fluorescence intensity (MFI) to evaluate the cellular uptake of AFMFlip^YOYO-1^, MFlip-a^YOYO-1^, and MFlip-b^YOYO-1^ without or with 10% FBS incubation. Data are presented as mean ± SD and statistically analyzed using two-tailed *t* test (*n* = 3 biologically independent samples). ns: no significance (*p* > 0.05). **e** Uptake inhibition assay of AFMFlip^YOYO-1^, MFlip-a^YOYO-1^, and MFlip-b^YOYO-1^ under the medium with different concentrations of FBS from 0% to 10% in the presence of different inhibitors. Data are presented as mean ± SD (*n* = 3 biologically independent samples). Source Data are provided as a Source Data file.

Strong protein-adsorption-resistance is the key to maintaining the membrane-fusion-mediated intracellular transport of AFMFlip under a serum protein-enriched physiological condition. Herein, we applied AFMFlip, MFlip-a, and MFlip-b to deliver plasmid cargos as the Methods described (Supplementary Fig. 11). To systematically demonstrate the above hypothesis, the dual-labeled plasmid-loaded MFlips (DiD-AFMFlip^YOYO-1^, DiD-MFlip-a^YOYO-1^, and DiD-MFlip-b^YOYO-1^), in which the plasmids and lipid shells were labeled with YOYO-1 and DiD respectively, were prepared to investigate cellular internalization in medium without or with 10% FBS by CLSM observation after 1-h incubation (Fig. 3a). These dual-labeled MFlips all performed obvious membrane-fusion interaction with HeLa cells in the FBS-free medium, as the fluorescent signals of the DiD-labeled shell (red) were mostly lay on the CellMask-labeled plasma membrane (cyan), while YOYO-1-labeled plasmids (green) were spread into the cytoplasm in the CLSM images (Fig. 3a and Supplementary Fig. 12a). Further results from fluorescence intensity line scanning analysis also indicated the fusion effect with lipid membrane merging and concomitant inner cargo release. However, when changing to a 10% FBS-containing medium, only DiD-AFMFlip^YOYO-1^ still interacted with cells via a membrane-fusion-based manner, while DiD-MFlip-a^YOYO-1^ and DiD-MFlip-b^YOYO-1^ were found with some overlapped red and green signals in the cytoplasm. Fluorescence intensity profiles of line scanning analysis further confirmed these results, implying the participation of endocytosis of MFlip-a and MFlip-b in the FBS-containing medium.

Furthermore, we quantified the membrane-fusion efficiency of AFMFlip, MFlip-a, and MFlip-b at various time points using FRET analysis. As shown in Supplementary Fig. 12b, AFMFlip, MFlip-a, and MFlip-b achieved about 90% fusion performance after 1 h cell incubation in the FBS-free medium. However, upon exposure to 10% FBS, only AFMFlip maintained a high efficiency of fusing with cells at 1 h incubation, whereas MFlip-a and MFlip-b showed significant declines. Propidium iodide (PI)-staining revealed <2% cell damage from all MFlips over time, maintaining membrane integrity post-treatment (Supplementary Fig. 12c).

For further cell-internalization mechanism validation, plasmid cargos were stained with YOYO-1 to form AFMFlip^YOYO-1^, MFlip-a^YOYO-1^, and MFlip-b^YOYO-1^, followed by incubation under 0% FBS or 10% FBS condition with HeLa cells that were pretreated with the inhibitors of different delivery pathways (Fig. 3b). These inhibitors included Z-Phe-Phe-Phe-OH (a membrane-fusion inhibitor)^18,38^, chlorpromazine (a clathrin-mediated endocytosis inhibitor)^39^, amiloride (a macropinocytosis-mediated endocytosis inhibitor)^40^, and nystatin (a caveolae-mediated endocytosis inhibitor)^41^. When incubated without FBS, the cell uptake of AFMFlip^YOYO-1^, MFlip-a^YOYO-1^, and MFlip-b^YOYO-1^ was conformably decreased only by the interference of Z-Phe-Phe-Phe-OH to 53.4%, 49.8%, and 46.4% respectively, suggesting their membrane-fusion interaction with cells (Fig. 3c). In the FBS-containing medium, AFMFlip^YOYO-1^ remained this trend with an uptake suppression of 46.8% only by Z-Phe-Phe-Phe-OH pretreatment but not any other endocytosis inhibitor. By contrast, the cellular uptake of MFlip-a ^YOYO-1^ and MFlip-b^YOYO-1^ in the serum-containing medium suffered significant influence by both Z-Phe-Phe-Phe-OH (27.3% and 40.0%, respectively) and chlorpromazine (39.8% and 35.1%, respectively). Z-Phe-Phe-Phe-OH + chlorpromazine treatment further decreased the internalization of MFlip-a^YOYO-1^ and MFlip-b^YOYO-1^ to 66.2% and 63.6%, respectively, confirming that they entered cells through both membrane fusion and clathrin-mediated endocytosis under the interference of serum proteins (Supplementary Fig. 13). Besides, the cellular uptake results showed that AFMFlip^YOYO-1^ kept its high uptake efficiency, while that of MFlip-a^YOYO-1^ and MFlip-b^YOYO-1^ was significantly impaired after serum incubation (Fig. 3d). These data collectively suggested that the protein corona formed on MFlip-a and MFlip-b will affect their internalization pathway, attenuating the membrane-fusion-mediated uptake but enhancing clathrin-mediated endocytosis. Excitingly, our established AFMFlip could effectively retain membrane-fusion-dependent behavior in the presence of serum protein-containing medium, resulting from the ability to alleviate protein corona formation.

**Fig. 4:**
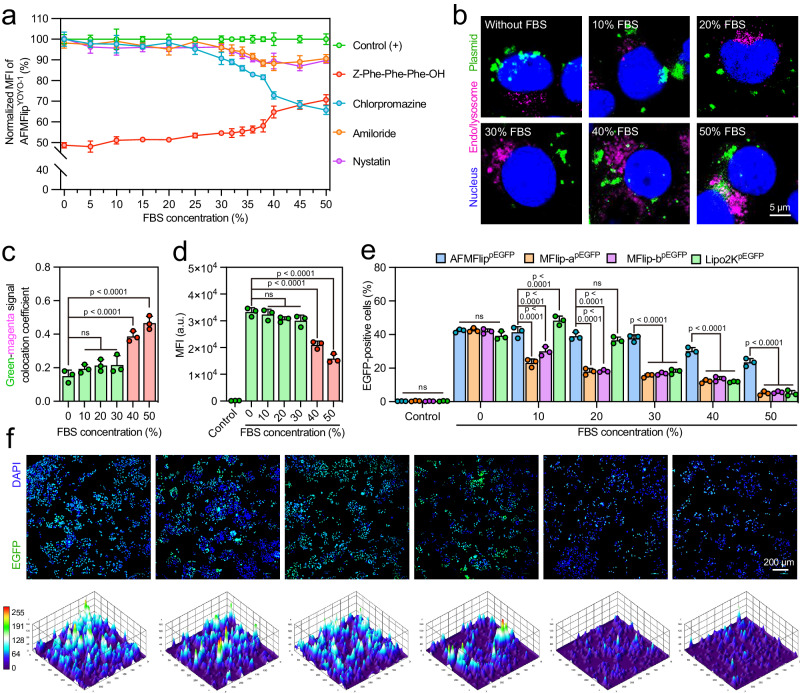
AFMFlip for serum-resistant membrane-fusion-mediated delivery. **a** Uptake inhibition assay of AFMFlip^YOYO-1^ with the medium containing 0-50% FBS. The critical protein-resistance concentration was set as the FBS concentration with a significant change in the transportation mechanism. Data are presented as mean ± SD (*n* = 3 biologically independent samples). **b** CLSM observation of HeLa cells co-incubated with AFMFlip^YOYO-1^ in the medium containing 0-50% FBS for 2 h, showing their intracellular trafficking process. The nuclei were stained with DAPI, and the cellular lysosomes were labeled with LysoTracker. The experiment was repeated three times independently with similar results. **c** Pearson’s coefficient index of the fluorescence signals between green and magenta based on **b** was determined by ImageJ software. Data are presented as mean ± SD and statistically analyzed using one-way ANOVA (*n* = 3 biologically independent samples). ns: no significance (*p* > 0.05). **d** MFI of cells incubated with AFMFlip^YOYO-1^ in the medium containing FBS at the concentration from 0% to 50%. Data are presented as mean ± SD and statistically analyzed using one-way ANOVA (n = 3 biologically independent samples). ns: no significance (*p* > 0.05). **e** EGFP transfection efficiency of AFMFlip^pEGFP^, MFlip-a^pEGFP^, MFlip-b^pEGFP^, and Lipo2K^pEGFP^ in the medium containing 0–50% FBS, analyzed via FACS. Data are presented as mean ± SD and statistically analyzed using one-way ANOVA (*n* = 3 biologically independent samples). ns: no significance (*p* > 0.05). **f** EGFP expression in HeLa cells observed by fluorescence microscopy transfected with AFMFlip^pEGFP^ at different FBS concentrations. The experiment was repeated three times independently with similar results. Source Data are provided as a Source Data file.

To determine the critical protein-resistance concentration, at which the membrane-fusion transportation would switch to endocytosis, we investigated the uptake in the presence of inhibitors with different concentrations of FBS (from 0 to 10%) (Fig. 3e). In this FBS concentration range, AFMFlip^YOYO-1^ still implemented the exclusive membrane-fusion-mediated uptake that was only affected by Z-Phe-Phe-Phe-OH treatment. Oppositely, once the FBS concentration increased to 4%, cell uptake of MFlip-a^YOYO-1^ in the presence of Z-Phe-Phe-Phe-OH was elevated obviously, while it decreased significantly in the presence of chlorpromazine, implying the transportation reversion from membrane fusion to endocytosis. Similar phenomena were found in the MFlip-b^YOYO-1^-treated group at a critical protein-resistance concentration of 6%. To further determine the critical protein-resistance concentration of AFMFlip, we incubated AFMFlip^YOYO-1^ with HeLa cells in the presence of inhibitors in the medium with higher FBS concentrations. Further exploration showed that in the medium containing up to 38% FBS, the membrane-fusion transportation of AFMFlip just began to convert to clathrin-mediated endocytosis (Fig. 4a), far exceeding the anti-FBS critical concentration of MFlip-a (4%) and MFlip-b (6%). We concluded that the FBS concentration of 38% could be the threshold for AFMFlip to maintain the dominant delivery route by membrane fusion. Collectively, these results demonstrated that AFMFlip could maintain membrane-fusion-mediated interactions with cells in the biologically relevant protein-enriched environment due to its unique antifouling capacity, superior to the previously reported MFlips.

### Protein-resistant plasmid transfection by AFMFlip

In our assumption, AFMFlip could be an efficient nanocarrier to deliver plasmid cargos via lysosome-independent membrane fusion in the plasma surroundings. To test this, AFMFlip^YOYO-1^, MFlip-a^YOYO-1^, and MFlip-b^YOYO-1^ were incubated with HeLa cells in the medium with 10% FBS, followed by observing the association of their intracellular trafficking with lysosomes. As depicted in Supplementary Fig. 14, cells treated with AFMFlip^YOYO-1^ were observed with negligible colocalization between the YOYO-1-labeled plasmids (green) and LysoTracker-labeled lysosomes (magenta) in the serum-containing environment, indicating the lysosome-bypassing intracellular route via stable membrane fusion by AFMFlip. In contrast, distinct fluorescence overlaps between plasmids and lysosomal compartments were shown in cells incubated with MFlip-a^YOYO-1^ and MFlip-b^YOYO-1^, suggesting extensive lysosomal trapping. For further protein-resistance delivery evaluation, the colocalization between AFMFlip^YOYO-1^ and cellular lysosomes was analyzed under gradually increasing concentrations of FBS (0, 10%, 20%, 30%, 40%, and 50%). Resultantly, the plasmids were located throughout the cells without any fluorescence overlap of lysosomal components, until the FBS concentration reached 40% or more (Fig. 4b). Pearson’s coefficient index calculations confirmed this result (Fig. 4c). Predictably, AFMFlip^YOYO-1^ maintained high uptake until the FBS concentration rose to 40% (Fig. 4d), in accordance with the previously-detected critical protein-resistance concentration (38%). Therefore, AFMFlip showed a higher resistance to proteins than traditional MFlips to deliver cargo into cells via a membrane-fusion-mediated lysosome-independent manner. Owing to this excellent protein-resistance membrane-fusion-mediated transportation, AFMFlip could achieve steadily enhanced green fluorescence protein (EGFP)-encoding plasmid (pEGFP) transfection (nearly 40% efficiency) until the FBS concentration rose to 30%, at which the transfection capacities of MFlip-a, MFlip-b, and commercial Lipofectamine 2000 (Lipo2K) significantly reduced to 15.4%, 16.7%, and 17.9%, respectively (Fig. 4e). Fluorescence images in Fig. 4f also showed that cells treated with AFMFlip^pEGFP^ exhibited relatively high EGFP expression even in the 30% FBS medium, verifying the protein-resistant membrane-fusion-mediated delivery for efficient plasmid transfection.

### In vivo protein-resistance capacity of AFMFlip

**Fig. 5:**
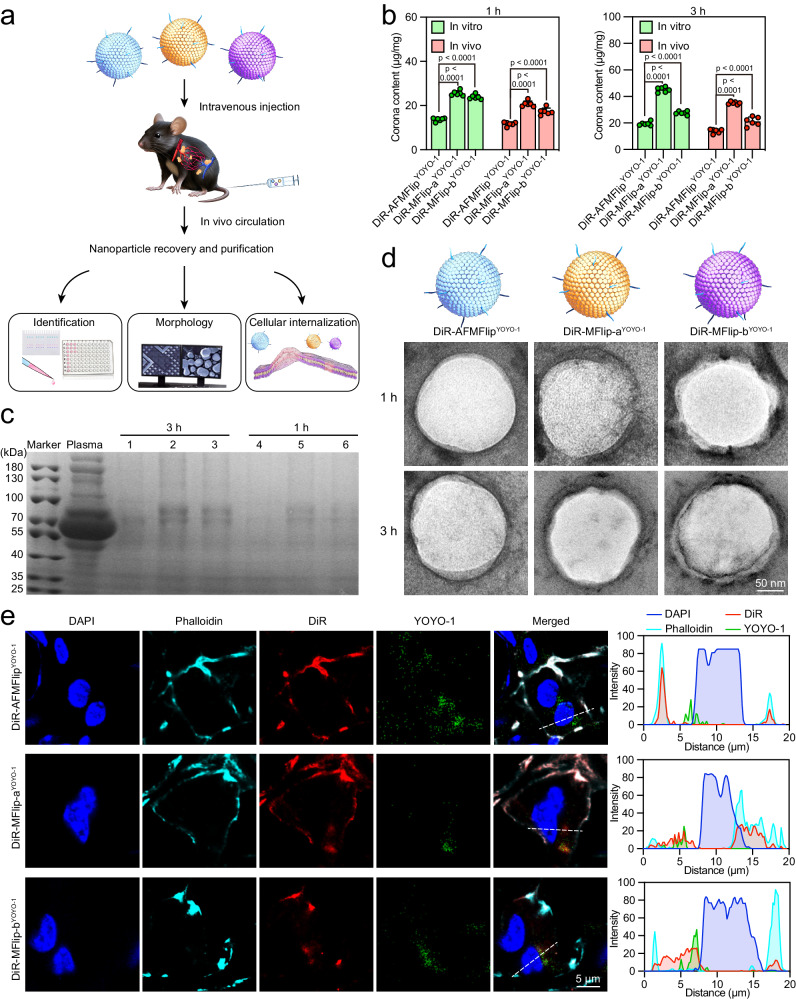
In vivo protein-adsorption resistance and intracellular transportation of AFMFlips. **a** Scheme of experimental design. **b** The BCA analysis for protein quantification. The in vitro mimicking corona was formed by incubating nanoparticles with 10% mouse serum. Data are presented as mean ± SD and statistically analyzed using one-way ANOVA (*n* = 6 biologically independent samples). **c** SDS-PAGE analysis of the recovered DiR-AFMFlip^YOYO-1^, DiR-MFlip-a^YOYO-1^, and DiR-MFlip-b^YOYO-1^ after in vivo administration. The experiment was repeated three times independently with similar results. **d** Transmission electron microscopy (TEM) observation of DiR-AFMFlip^YOYO-1^, DiR-MFlip-a^YOYO-1^, and DiR-MFlip-b^YOYO-1^, recovered after intravenous injection for 1 h and 3 h. The experiment was repeated three times independently with similar results. **e** CLSM observation of the liver tissues from mice with the treatment of DiR-AFMFlip^YOYO-1^, DiR-MFlip-a^YOYO-1^, and DiR-MFlip-b^YOYO-1^ via intravenous injection for 3 h. The nuclei were stained with DAPI, and the cell membranes were labeled with Phalloidin. Colocalization profiles are shown on the right side and were analyzed along the white lines by ImageJ software. The experiment was repeated three times independently with similar results. Source Data are provided as a Source Data file.

Inspired by the above results, we next evaluated the protein corona formation and intracellular delivery behaviors in vivo (Fig. 5a). Generally, we intravenously injected DiR-AFMFlip^YOYO-1^, DiR-MFlip-a^YOYO-1^, and DiR-MFlip-b^YOYO-1^ into C57BL/6 mice respectively, followed by recovering the injected nanoparticles from the blood circulation via size exclusion chromatography and membrane ultrafiltration^42^. The successful recovery of MFlips was demonstrated by dynamic light scattering (DLS) measurements and Stewart assays (Supplementary Fig. 15-19). These recovered nanoparticles were applied to identify and quantify their surface protein corona using BCA assay, SDS-PAGE analysis, and transmission electron microscopy (TEM) observation. BCA quantification showed an average total protein content of about 11.7 µg mg^−1^ and 13.8 µg mg^−1^ on DiR-AFMFlip^YOYO-1^ after 1 h and 3 h post-administration respectively, much lower than that on DiR-MFlip-a^YOYO-1^ (20.8 µg mg^−1^ and 29.2 µg mg^−1^) and DiR-MFlip-b^YOYO-1^(17.3 µg mg^−1^ and 21.2 µg mg^−1^) (Fig. 5b). This result highly coincided with protein quantification results after incubating with 10% mouse serum in vitro (Fig. 5b). Similarly, SDS-PAGE analysis exhibited the lightest electrophoretic band from recycled DiR-AFMFlip^YOYO-1^, indicating the least protein corona formation after the in vivo circulation for 1 h and 3 h (Fig. 5c). Consistently, TEM images in Fig. 5d displayed that the thickness of absorbed protein layering the surface of DiR-AFMFlip^YOYO-1^ was much thinner than that of DiR-MFlip-a^YOYO-1^ and DiR-MFlip-b^YOYO-1^ at 3 h after the circulation. These results consistently affirmed that AFMFlip possesses strong adsorption resistance when encountering protein-enriched surroundings in vivo.

This strong serum protein-adsorption-resistance capacity ensures the subsequent membrane-fusion-mediated delivery in vivo, which was demonstrated by CLSM observation of the localization of plasmids and lipid shells in the liver tissue. As shown in Fig. 5e and Supplementary Fig. 20, liver cells from mice treated with DiR-AFMFlip^YOYO-1^ showed observable fluorescence overlay between the DiR-labeled lipid layers (red) with the phalloidin-labeled membrane (cyan), while the YOYO-1-labeled plasmids (green) diffused in the cell cytoplasm. Comparably, the treatments with the other two MFlips resulted in some DiR fluorescence colocalization with the YOYO-1 fluorescence inside the cells, implying the coexistence of both fusion and endocytosis. Accordingly, AFMFlip could ensure the exclusive membrane-fusion-mediated transport after in vivo circulation owing to the excellent antifouling effect, allowing efficient intracellular delivery without lysosomal entrapment.

### In vivo luciferase transfection using AFMFlips

**Fig. 6:**
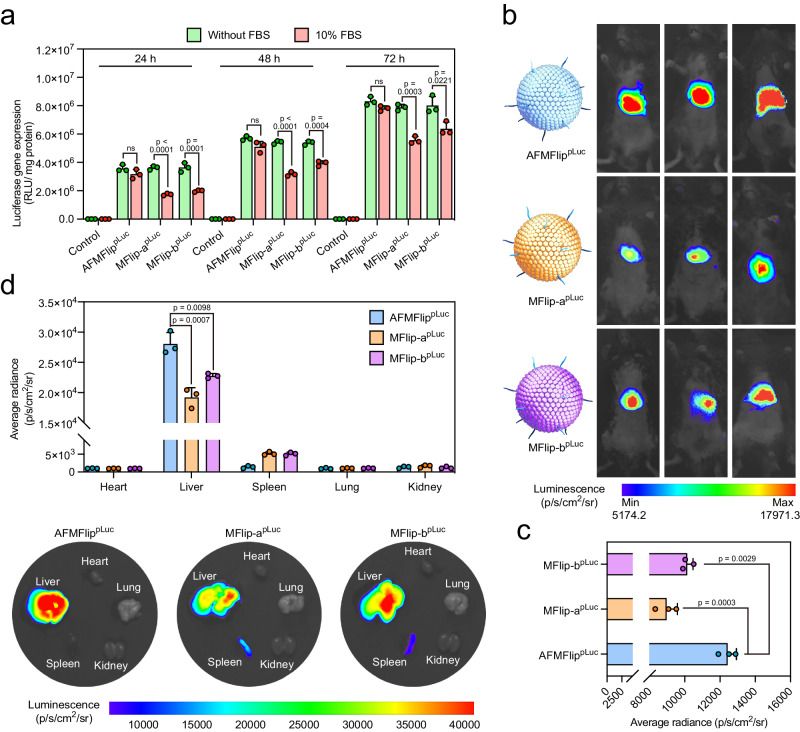
In vivo luciferase transfection using AFMFlips. **a** Luciferase transfection efficiency in HeLa cells with PBS (control), AFMFlip^pLuc^, MFlip-a^pLuc^, and MFlip-b^pLuc^ without or with serum for 24, 48, and 72 h. The transfection dose of the luciferase plasmid was 0.5 μg mL^−1^). Data are presented as mean ± SD and statistically analyzed using two-tailed *t* test (*n* = 3 biologically independent samples). ns: no significance (*p* > 0.05). **b** In vivo bioluminescence images of mice at 48 h after receiving an intravenous injection of AFMFlip^pLuc^, MFlip-a^pLuc^, and MFlip-b^pLuc^ (*n* = 3 biologically independent animals). **c** Average bioluminescence intensity of mice treated with AFMFlip^pLuc^, MFlip-a^pLuc^, and MFlip-b^pLuc^ after 48 h. Data are presented as mean ± SD and statistically analyzed using one-way ANOVA (*n* = 3 biologically independent animals). **d** Representative ex vivo bioluminescence images of luciferase expression and corresponding quantification analysis in major organs (heart, liver, spleen, lung, and kidney) from mice at 48 h post-injection of AFMFlip^pLuc^, MFlip-a^pLuc^, and MFlip-b^pLuc^. The above bioluminescence images were taken at 10 min after intraperitoneal administration of D-luciferin. Data are presented as mean ± SD and statistically analyzed using one-way ANOVA (*n* = 3 biologically independent animals). Source Data are provided as a Source Data file.

Considering the main accumulation of AFMFlips in the liver after intravenous injection (Supplementary Fig. 21), we sought to apply AFMFlip as the carrier to deliver luciferase plasmids to the mouse liver for efficient expression. Before the application in vivo, we preliminarily evaluated the capacity of AFMFlips to efficiently deliver plasmids encoding luciferase reporter gene (pLuc) in HeLa cells in vitro without or with FBS. As depicted in Fig. 6a, in the serum-free medium, AFMFlip^pLuc^, MFlip-a^pLuc^, and MFlip-b^pLuc^ all induced equivalent bioluminescent expression levels, which increased with time. When exposed to a 10% FBS-containing medium, only the cells transfected with AFMFlip^pLuc^ retained high transfection efficiency. This protein interference decreased the luciferase expression of MFlip-a^pLuc^ by 51.9% at 24 h, 41.3% at 48 h, and 29.4% at 72 h, respectively, as well as MFlip-b^pLuc^ by 45.3% at 24 h, 26.9% at 48 h, and 20.5% at 72 h, respectively.

Subsequently, we investigated the luciferase gene delivery efficiency in mice by intravenous administration of the formulated AFMFlip^pLuc^, MFlip-a^pLuc^, and MFlip-b^pLuc^. After 48 h post-injection, bioluminescence was evaluated by the imaging of living bodies and harvested organs. The results revealed that the mice treated with AFMFlip^pLuc^ exhibited the highest level of luciferase intensity at the liver region, which was 1.39-fold and 1.23-fold to MFlip-a^pLuc^ and MFlip-b^pLuc^ treatment, respectively (Fig. 6b, c). In addition, ex vivo imaging of the major organs (liver, kidney, lung, spleen, and heart) from mice supported the most efficient luciferase plasmid delivery for liver tissue-specific expression using AFMFlip, outperforming the other two MFlips (Fig. 6d). These results demonstrated that AFMFlip could successfully achieve efficient membrane fusion-mediated delivery under physiological conditions, suggesting the potential in therapeutic nanomedicine for application in vivo.

### In vivo CRISPR/Cas9 delivery for HBV therapy

**Fig. 7:**
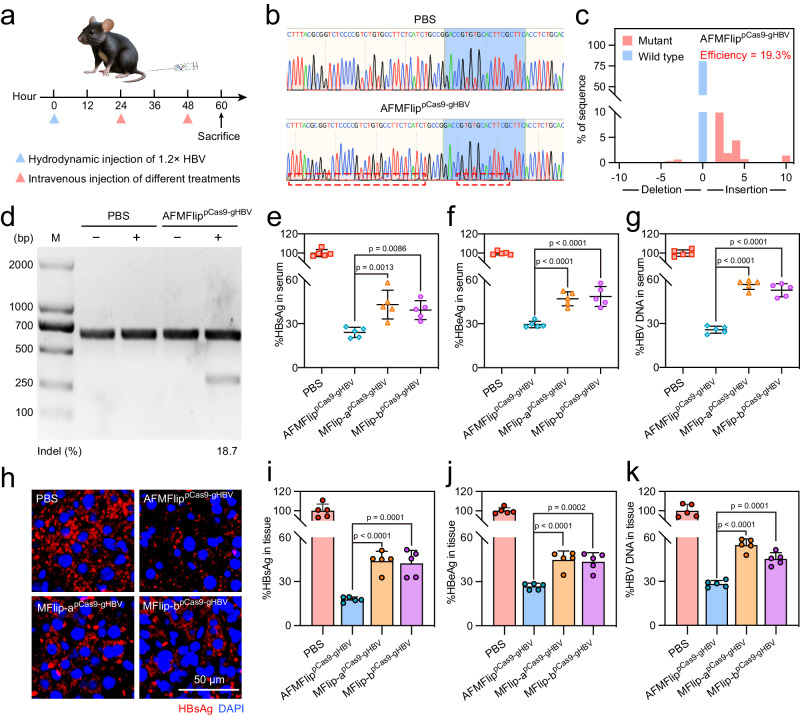
AFMFlip-based nanomedicine for hepatitis B virus (HBV) infection treatment in mice. **a** Schematic illustration of the protocol of PBS, AFMFlip^pCas9-gHBV^, MFlip-a^pCas9-gHBV^, and MFlip-b^pCas9-gHBV^ treating HBV infection. **b** Sanger DNA sequencing results of the gene editing at HBV-targeting sites amplified from the liver tissue DNA of mice treated with PBS and AFMFlip^pCas9-gHBV^. The experiment was repeated three times independently with similar results. **c** Tracking of indels by decomposition (TIDE) analysis of sequencing results at the targeted HBV genome from liver tissue DNA of mice treated with AFMFlip^pCas9-gHBV^. The editing efficiency for TIDE analysis was calculated online (https://tide.nki.nl/). The experiment was repeated three times independently with similar results. **d** T7 endonuclease I (T7E1) assay at the HBV-targeting site in the liver tissue DNA from mice treated with PBS and AFMFlip^pCas9-gHBV^. The cutting efficiency of indel was determined by band densitometry using ImageJ software. The experiment was repeated three times independently with similar results. **e**–**g** Analysis of HBV-related viral loads in the serum from HBV-replication mice treated with PBS, AFMFlip^pCas9-gHBV^, MFlip-a^pCas9-gHBV^, and MFlip-b^pCas9-gHBV^, including HBsAg (**e**), HBeAg (**f**), and HBV DNA (**g**). Data are presented as mean ± SD and statistically analyzed using one-way ANOVA (*n* = 5 biologically independent animals). **h** Immunofluorescence staining of HBsAg in the liver sections harvested from HBV-replication mice treated with PBS, AFMFlip^pCas9-gHBV^, MFlip-a^pCas9-gHBV^, and MFlip-b^pCas9-gHBV^. The experiment was repeated three times independently with similar results. **i**–**k** Determination analysis of HBsAg (**i**), HBeAg (**j**), HBV DNA (**k**) from liver tissues. All values shown were normalized to the negative control group with PBS treatment. Data are presented as mean ± SD and statistically analyzed using one-way ANOVA (*n* = 5 biologically independent animals). Source Data are provided as a Source Data file.

With the comprehensive validation of serum-resistance membrane fusion-mediated delivery, AFMFlip was utilized to design a clustered regularly interspaced short palindromic repeats/associated protein 9 (CRISPR/Cas9)-based nanomedicine to cleave viral genome for HBV treatment^43–46^. Herein, we inserted the commercial CRISPR/Cas9 plasmid (pX330) with the guide RNA site targeting open reading frameworks of HBV viral sites encoded with polymerase- (P) and X protein (X) to form pCas9-gHBV for AFMFlip encapsulation (Supplementary Fig. 22). To evaluate the in vivo gene editing by AFMFlip^pCas9-gHBV^ to suppress HBV, the HBV-replication mouse model was established by hydrodynamic injection of the plasmid encoding the HBV genome 1.2 times (pBB4.5–1.2× HBV) to mice via tail vein^47–49^. After 24-h injection, these mice were divided into four groups in random, followed by intravenous injection of PBS, AFMFlip^pCas9-gHBV^, MFlip-a^pCas9-gHBV^, and MFlip-b^pCas9-gHBV^ for 2 dosages, respectively (Fig. 7a). The biosafety of AFMFlip^pCas9-gHBV^ was investigated by the blood biochemistry tests of serum samples, the observation of body weight, and the histopathological analysis of major organs after treatment. As a result, negligible changes in the serum levels of albumin (ALB), alanine aminotransferase (ALT), aspartate aminotransferase (AST), alkaline phosphatase (ALP), blood urea nitrogen (BUN), creatine kinase (CK), creatinine (CREA), and lactate dehydrogenase (LDH) were detected, and less than 5% of body weight changes were observed in mice after receiving all those treatments (Supplementary Fig. 23). Furthermore, the hematoxylin and eosin (H&E) staining of tissues of major organs (heart, liver, spleen, lung, and kidney) in mice from different treatment groups showed no obvious pathological damage compared with that from the healthy mice (Supplementary Fig. 24). All these results indicated the minimal toxicity of AFMFlip for in vivo application.

To evaluate the in vivo CRISPR/Cas9-based gene-editing efficiency, we extracted the DNA from mouse liver tissues for Sanger DNA sequencing and T7 endonuclease I (T7E1)-based analysis of the HBV genome at the target locus. Sanger DNA sequencing maps at the HBV-targeting site of the liver tissue DNA from HBV-replication mice with AFMFlip^pCas9-gHBV^ treatment showed a distinct composite sequence trace compared with PBS treatment, confirming the induced mutations by gene editing (Fig. 7b). Furthermore, the online tracking of indels by decomposition (TIDE) analysis of Sanger DNA sequencing quantified the gene-editing efficiency of AFMFlip^pCas9-gHBV^ treatment as 19.3%, much higher than that of MFlip-a^pCas9-gHBV^ (13.4%) and MFlip-b^pCas9-gHBV^ (15.1%) (Fig. 7c and Supplementary Fig. 25). Moreover, the electrophoretogram analysis of T7E1-incubated DNA in the liver from mice with AFMFlip^pCas9-gHBV^ treatment exhibited obvious cleavage bands at the targeted site compared to the PBS treatment, with an 18.7% mutation frequency (Fig. 7d). The above results revealed that AFMFlip^pCas9-gHBV^ could perform potent gene editing at the HBV-targeting site in vivo.

With this potent efficiency of the CRISPR/Cas9-based gene editing, HBV-related viral loads both in the serum and in liver tissues should be downregulated, as demonstrated by detecting hepatitis B surface antigen (HBsAg), hepatitis B e antigen (HBeAg), and HBV DNA. In contrast to the control group (PBS), mice with AFMFlip^pCas9-gHBV^ treatment had an obvious decrease in the serum levels of HBsAg by 75.9%, HBeAg by 70.5%, and HBV DNA by 74.2% respectively, significantly lower than that treated with MFlip-a^pCas9-gHBV^ (56.9%, 53.0%, and 43.3%, respectively) and MFlip-b^pCas9-gHBV^ (60.8%, 52.4%, and 47.2% respectively) (Fig. 7e–g). The immunofluorescence analysis of the liver-residual HBsAg also verified these results, exhibiting the least immunofluorescence signal of HBsAg in the liver tissues after AFMFlip^pCas9-gHBV^ treatment (Fig. 7h). In addition, AFMFlip^pCas9-gHBV^ treatment could also lead to a remarkable suppression of HBsAg (81.2%), HBeAg (73.2%), and HBV DNA (71.7%) in the liver tissues from HBV-replication mice, more effective than that treated with MFlip-a^pCas9-gHBV^ (56.1%, 55.1%, and 45.2% respectively), and MFlip-b^pCas9-gHBV^ (57.5%, 56.4%, and 58.7% respectively) (Fig. 7i–k). Collectively, this excellent anti-HBV efficacy illustrated that the AFMFlip is suitable for delivering therapeutics in vivo for effective disease treatment.

## Discussion

Membrane fusion is a promising intracellular delivery strategy, through which cargos can be directly released into the cytoplasm without being trapped in lysosomes^50,51^. Recently developed MFlips could perform biomimetic membrane-fusion-based delivery. This functionality was attributed to the similar chemical structure, fluidity, and phase-transition behavior of lipid materials with cell membrane lipid layers^5,9,18,21^. Such membrane-fusion systems possess the dominant advantages of great biocompatibility, high cargo-loading capacity, feasible designability, and easy preparation to deliver broad therapeutic medicines, including nucleic acids, proteins, and chemical drugs. However, they are challenged with poor reproducibility of the membrane-fusion effect in the complex physiological environment, which greatly limits their further in vivo applications and nanomedicine translation to the clinic.

The protein corona formed on the MFlip surface during body circulation is the main cause of a failure of membrane-fusion-based transportation in vivo. The integration of antifouling zwitterionic materials on the MFlip surface can prevent protein corona formation, thereby sustaining the membrane-fusion capability under protein-enriched physiological conditions. We chose DOPC, with a phosphorylcholine heading group, as the neutral lipid. We screened the optimal molecular ratio for the membrane-fusion effect combined with a cationic DOTAP and DSPE-PEG in the presence or absence of protein-enriched conditions (Fig. 2). As expected, the constructed AFMFlip with abundant zwitterionic DOPC showed an excellent anti-protein-adsorption capacity, enabling them to achieve stable membrane-fusion-mediated delivery in the medium containing up to 38% FBS, far outperforming the conventional MFlip-a (4%) and MFlip-b (6%) (Figs. 3–4). In vivo investigations also confirmed the performance of AFMFlips for gene transfection and CRISPR/Cas9-based HBV inhibition up to nearly 20% gene-editing efficiency (Figs. 5–7). We verified that the strong inhibition of protein corona formation on the AFMFlip surface endowed its maintaining membrane-fusion effect in the protein-enriched physiological environment, achieving enhanced delivery efficiency for in vivo nanomedical applications. Considering that these lipid composites are commonly used clinically, AFMFlip has the potential to accelerate the translation of extensive nanomedicines. Further exploration should be emphasized on improving their cell-type targeting selectivity by such as tissue/cell-specific peptide modification^52,53^. Moreover, the protein-resistant membrane-fusion-mediated delivery efficiency of AFMFlips can also be optimized by combining other anti-protein-adsorption molecules with a superior antifouling effect.

In summary, we have developed an AFMFlip with strong protein-adsorption resistance to accomplish stable and efficient membrane-fusion-mediated intracellular delivery in vivo. The integration of zwitterionic DOPC on the surface enables AFMFlips to adsorb fewer proteins, alleviating corona formation. Consequently, AFMFlip, with a specific DOPC/DOTAP/DSPE-PEG ratio (90/5/5), can not only facilitate a membrane-fusion-mediated intracellular delivery without lysosomal entrapment, but also sustain this functionality in the serum-enriched environment due to the abundant zwitterionic DOPC. Such effect of AFMFlips ensures the effective gene delivery in vitro, efficient luciferase transfection in vivo, and improved gene-editing efficiency of CRISPR/Cas9-based therapeutics in the in vivo HBV model. This AFMFlip can serve as a promising tool for efficient in vivo intracellular delivery of various drugs, excavating more extensive nanomedical applications.

## Methods

### Ethical statement

The research presented here complies with all relevant ethical regulations. All experiments involving animals complied with the Guidelines for Care and Use of Laboratory Animals of Sun Yat-sen University and were approved by the Institutional Animal Care and Use Committee of Sun Yat-sen University.

### Materials

1,2-dimyristoyl-*sn*-glycero-3-phosphocholine (DMPC), 1,2-dioleoyl-*sn*-glycero-3-phosphocholine (DOPC), 1,2-dioleoyl-*sn*-glycero-3-phosphoethanolamine (DOPE), 1,2-dioleoyl-3-trimethylammonium-propane (DOTAP), and 1,2-distearoyl-*sn*-glycero-3-phosphoethanolamine-*N*-[methoxy(polyethylene glycol)-2000] (DSPE-PEG) were obtained from Sunlipo Biotech Research Center for Nanomedicine (Shanghai, China). Cholesterol was obtained from Merck Ltd. (Beijing, China). Ammonium thiocyanate was bought from Macklin Inc. (Shanghai, China). DiD, DiR, and polymerase chain reaction (PCR) kit with Taq reagent were purchased from Beyotime Biotechnology Co., Ltd. (Shanghai, China). DiO, DiI, YOYO-1, LysoTracker Red (DND-99), and D-Luciferin luciferase substrate were bought from Yeasen Co., Ltd. (Shanghai, China). pCDNA3.1-Luc encoding luciferase (catalog: #185358) was purchased from Miaoling Biotechnology Co., Ltd. (Wuhan, China). CellMask plasma membrane stain and lipofectamine 2000 were bought from Thermo Fisher Scientific, Inc. (Pittsburgh, PA, USA). The commercial CRISPR/Cas9 plasmid (pX330, catalog: #42230) was purchased from Addgene (USA). The 1.2× HBV expression plasmid (pBB4.5–1.2×HBV) was kindly provided by Professor Ran Chen (Zhongshan School of Medicine, Sun Yat-sen University). The gRNA targeting HBV (5’-GACCGTGTGCACTTCGCTTC-3’) and PCR primers (5’-GGGTACCCAGCAGGTCTGGAGCAAA-3’ and 5’-GGAATTCGGAAAGAAGTCAGAAGGCAAA-3’) were purchased from Tsingke Biotechnology Co., Ltd (Beijing, China). The polyclonal anti-HBsAg antibody (catalog: #bs-1557G) was bought from Bioss Biotechlonogy Co., Ltd (Beijing, China). The Cy3-conjugated donkey anti-goat IgG H&L (catalog: #GB21404) was obtained from Servicebio Technology Co., Ltd (Wuhan, China). T7 Endonuclease I (T7E1) was bought from New England Biolabs, Inc. (Ipswich, MA, USA). The blood/cell/tissue DNA Isolation Kit was bought from Vazyme Biotech Co., Ltd. (Nanjing, China). SanPrep Column DNA Gel Extraction Kit was obtained from Sangon Biotechnology Co., Ltd. (Shanghai, China). The HBsAg, HBeAg, and HBV DNA ELISA kits were obtained from Shanghai Fusheng Industrial Co., Ltd. (Shanghai, China).

### Cell line and animal experiments

The human cervical cancer cell line HeLa (catalog: #SCSP-504) was purchased from the National Collection of Authenticated Cell Cultures (Shanghai, China) and cultured in DMEM with 10% FBS and 1% penicillin/streptomycin at 37 °C.

All animal experiments complied with Guidelines for Care and Use of Laboratory Animals of Sun Yat-sen University and approved by the Institutional Animal Care and Use Committee of Sun Yat-sen University (protocol number: SYSU-IACUC-2021-000714). Male C57/BL mice (3–5 weeks old) were obtained from the Laboratory Animal Center of Sun Yat-sen University (Guangzhou, China). The mice were housed under SPF conditions and fed with standard food and water. The room was maintained at a controlled temperature of 25 °C and humidity of 30%-70% with a 12 h light/dark cycle.

### Synthesis of membrane-fusogenic liposomes

The core of the plasmid/CaCO_3_ was constructed as follows. Solution 1 (50 μL) contained a mixture of CaCl_2_ solution (0.5 M, 16 μL), plasmid (1 μg μL^−1^, 1 μL), and sterile deionized water (33 μL). Solution 2 (50 μL) was obtained by mixing Na_2_CO_3_ solution (0.01 M, 16 μL) and sterile deionized water (34 μL). Next, solution 2 was pipetted gently to solution 1 to obtain a mixing solution (100 μL) with CaCO_3_ nanoparticles carrying plasmids.

The core-shell structured AFMFlip was prepared using the following film hydration/extrusion method. Briefly, DSPE-PEG, DOPC, and DOTAP were dissolved in chloroform and stored at 4 °C. DiD or DiR (5 mM) was mixed in the organic solvent for lipophilic dye incorporation. Then, lipid films of DSPE-PEG, DOPC, and DOTAP with specific molar ratios were prepared by evaporating the organic solvent and then drying in a vacuum desiccator overnight. Next, the lipid films were rehydrated with the solutions of plasmid-loaded CaCO_3_ nanoparticles. The resultant mixture solution was repetitively extruded under polycarbonate nanoporous membranes with a decreasing pore size (800, 400, and 200 nm). Similarly, traditional membrane-fusogenic liposomes (MFlips) are synthesized with the same processes but using different lipid components at different molar ratios according to the previous reports (Supplementary Fig. 11a). Finally, the solution containing the plasmid-loaded nanoparticles underwent purification through centrifugation at 21,000 × *g* for 50 min at 4 °C. Subsequently, the precipitates were collected. To determine the encapsulation efficiency of plasmids within nanoparticles, the concentration of unencapsulated free plasmid in the supernatant of the solution after centrifugation was quantified using a NanoDrop 2000 spectrophotometer (Wilmington, DE, USA). The encapsulation efficiency was calculated using the following formula: *EE* (%) = (*CT*–*CF*)/*CT* × 100%, where *EE* represents the encapsulation efficiency, *CT* is the total concentration of plasmids, and *CF* denotes the concentration of unencapsulated, free plasmids in the supernatant.

### Physicochemical characterization

Nanoparticle size and zeta potential were measured by a dynamic light scattering (DLS) system with a Litesizer 500 particle analyzer (Anton Paar, Austria) at room temperature. The images of morphology were acquired by transmission electron microscopy (FEI Tecnai G2 F30, Philips-FEI) at a 120 kV accelerating voltage. Freshly prepared samples were dropped onto the 200-mesh carbon-coated copper grid. The excess solvent was dried off after 10 min, and then 5 μL of phosphotungstic acid was applied for negative staining.

### Membrane fusion effect observation

For the optimization of stable membrane fusion in the FBS-containing medium, the various DiD-labeled MFlips consisting of DOPC, DSPE-PEG, and DOTAP were prepared with different molar ratios (DSPE-PEG/DOPC/DOTAP = 5/95/0, 5/90/5, 5/85/10, 5/75/20, 5/65/30). HeLa cells in 2 mL of DMEM culture medium were seeded in a 6-well plate at a density of 2 × 10^5^ per well and incubated at 37 °C for 24 h. The medium was then replaced with 1 mL of DMEM solution containing various nanoparticles. After 1-h co-incubation, cells were washed carefully with PBS, and the plasma membranes were labeled with CellMask. Then, DAPI was used to stain the cell nuclei for 10 min, followed by another careful PBS washing. After that, cells were observed by CLSM imaging (Leica STELLARIS STED).

The DiD-labeled AFMFlip (DSPE-PEG/DOPC/DOTAP/DiD = 5/90/5/5), DiD-labeled MFlip-a (DSPE-PEG/DMPC/DOTAP/DiD = 3.8/76.2/20.0/5.0), and DiD-labeled MFlip-b (DSPE-PEG/DOPE/CHEMS/DiD = 12.3/69.5/18.2/5.0) were then constructed. The plasmid cargos were labeled with YOYO-1 following the manufacturer’s instructions. HeLa cells in 2 mL of DMEM culture medium were seeded in a six-well plate at a density of 2 × 10^5^ cells per well and incubated at 37 °C for 24 h. The medium was then replaced with 1 mL of DMEM solution containing various formulations (2000 ng mL^−1^ plasmid). After 1-h co-incubation, cells were washed carefully with PBS, and the plasma membranes were labeled with CellMask. Then, DAPI was used to stain the cell nuclei for 10 min, followed by another careful PBS washing. After that, cells were observed by CLSM imaging.

### FRET-based membrane-fusogenic quantitative assay

The liposomes were labeled with both DiO and DiI, which can exhibit the FRET effect. When these liposomes fused with cell membranes, the increased distance significantly reduced FRET efficiency^35,36^. The prepared liposomes were added to HeLa cells at a density of 2 × 10^6^ cells per mL without or with 10% FBS for 1 h at 37 °C. The initial FRET effect of the liposome (excitation using Ex (DiO) = 483 nm, but emission at Em (DiI) = 565 nm) was measured as 0% fusion using a Tecan Spark spectrofluorometer. The spectrum of 100% fusion was determined after adding Triton X-100 at 0.1% (v/v). After incubation with cells in the presence/absence of 10% FBS for 1 h at 37 °C, the fluorescence spectra of liposome/cell mixtures were recorded to determine the membrane-fusion performance using the formula: *Fusion* (%) = (*F*_*DiO*_−*F0*)/(*F100*−*F0*).

To determine the quantitative membrane-fusion efficiency of AFMFlip, MFlip-a, and MFlip-b at different time intervals, liposomes labeled with DiO and DiI were added into the cells in a 96-well plate in the presence/absence of 10% FBS with different incubation times at 37 °C. The fluorescence intensity of DiO was measured at an excitation of 483 nm and an emission of 501 nm using a microplate reader (BioTek Synergy).

### Analysis of protein corona on MFlips

The solution containing 20% (v/v) FBS or mouse serum was added to the equivalent solution containing AFMFlip, MFlip-a, or MFlip-b. Then, the mixture was incubated with shaking at 100 × *g* for 1 h at 37 °C. To remove unbound serum proteins, the liposomes were pelleted by centrifugation at 21,000 × *g* for 50 min at 4 °C. After that, the collected precipitates were washed carefully with sterile deionized water, followed by measurements of particle sizes and zeta potentials at room temperature.

Following the manufacturer’s instructions, these precipitates dispersed in PBS were detected by the BCA to calculate protein concentrations. Generally, the standard curve was performed by a gradient concentration dilution to a protein standard sample, starting from 1 μg/mL. The reagents A and B at a 50:1 mixed ratio were added with 200 μL per well in the 96-well plate. The standard or unknown samples were dispensed with 20 μL of each well, followed by a 30-min incubation at 37 °C. After that, the microplate reader (BioTek Synergy) was used to measure the absorbance at 574 nm.

For the SDS-PAGE analysis, the samples were resuspended in the SDS-PAGE loading buffer, followed by 10-min sonication. After that, the solutions were boiled for five min, and then they were loaded in 4–20% Tris-Gly Plus Precast PAGE Gel (Beyotime). The gel was started by running at 80 mV for 5 min, then at 120 mV until the loading buffer band reached the end of the gel. The dying process with EZ Blue Gel was carried out overnight at room temperature; subsequently, samples were washed with distilled water for 2 h.

For liquid chromatography-tandem mass spectrometry (LC-MS/MS) analysis, the proteins were separated with 12.5% SDS-PAGE (constant current 14 mA, 90 min) and then digested in-gel with trypsin. The peptides were analyzed by a Q Exactive mass spectrometer (Thermo Scientific) that was coupled to Easy nLC (Proxeon Biosystems, now Thermo Fisher Scientific) for 60/120/240 min. The spectra were under analysis via the MaxQuant engine. The mass tolerance was twenty ppm. In the meantime, MS/MS tolerance was 0.1 Da. The UniProt database was searched for protein identification.

### Membrane-fusion mechanism investigation

In 500 μL of DMEM culture medium, HeLa cells were seeded in a 24-well plate at a density of 5 × 10^4^ cells per well and incubated at 37 °C for 24 h. Z-Phe-Phe-Phe-OH (a membrane-fusion transportation mediator), chlorpromazine (an inhibitor of clathrin-mediated endocytosis), amiloride (an inhibitor of macropinocytosis), and nystatin (an inhibitor of caveolae-mediated endocytosis) were added separately to the medium at their recommended concentrations (100 μg mL^−1^, 10 μg mL^−1^, 100 μg mL^−1^, and 15 μg mL^−1^, respectively). After the pretreatments of different inhibitors for 30 min, these solutions were replaced with DMEM solutions containing various MFlip formulations with the plasmid concentration of 2000 ng mL^−1^ in different FBS concentrations. After incubation for two hours, the cells were removed with the medium followed by careful PBS washing, collected, and then analyzed by flow cytometry using a BD FACS Calibur flow cytometer (BD Biosciences) equipped with a 488 nm laser and a 525/40 nm filter to obtain the mean fluorescence intensity (MFI) from YOYO-1-labeled plasmids.

### Cell uptake and intracellular distribution analysis

Before the experiments, HeLa cells in 2 mL of DMEM culture medium were seeded in a 6-well plate at a density of 2 × 10^5^ cells per well and incubated at 37 °C for 24 h. The medium was then replaced with 1 mL of DMEM solution containing various formulations (2 μg mL^−1^ plasmid) in different concentrations of FBS, in which the plasmid cargos were labeled with YOYO-1 following the manufacturer’s instructions. After staining for two hours, the cells were removed with the medium, followed by careful PBS washing, collected, and then analyzed by flow cytometry to obtain the MFI.

For intracellular distribution analysis, after co-incubation for 1 h, the cells were washed three times with PBS. Then, the cells were stained with LysoTracker Red for 30 min at 37 °C. Then the nuclei were stained with DAPI for 10 min. After three PBS washes, the cells were imaged by CLSM.

### Effects of FBS concentration on gene transfection of AFMFlips

For EGFP gene transfection, HeLa cells were seeded in a 24-well plate at a density of 5 × 10^4^ cells per well and incubated at 37 °C for 24 h. The medium was then replaced with 1 mL of DMEM solution containing AFMFlip^pEGFP^ (2 μg mL^−1^ plasmid) in a variety of FBS concentrations (0%, 10%, 20%, 30%, 40%, and 50%). After 48-h transfection, the cells were removed with the medium followed by careful PBS washing, then either collected for EGFP-positive cell analysis by the flow cytometer equipped with a 488 nm laser and a 525/40 nm filter, or the cells were applied to a 10-min DAPI staining for imaging by an inverted fluorescence microscope (Nikon Ti2-U).

### In vivo investigation of the anti-protein-adsorption capacity of MFlips

DiR-AFMFlip^YOYO-1^, DiR-MFlip-a^YOYO-1^, and DiR-MFlip-b^YOYO-1^ were intravenously administrated into the C57BL/6 mice (male, 3–5 weeks). After injection for 1 h and 3 h, the nanoparticles were recovered by collecting nearly 500 μL blood samples in tubes through cardiac puncture. After striation at 4 °C overnight, the samples were centrifugated for ten min at 1500 × g at 4 °C, followed by the supernatant collection of plasma.

Subsequently, size exclusion chromatography was performed to separate excess plasma proteins from the nanoparticles of DiR-AFMFlip^YOYO-1^, DiR-MFlip-a^YOYO-1^, and DiR-MFlip-b^YOYO-1^. Generally, each of the above-collected plasma was loaded onto a Sepharose CL-4B column with PBS equilibration. Then, 30 chromatographic fractions (1 mL per fraction) were collected. Each chromatographic fraction was analyzed by DLS and Stewart assay. To quantify lipid concentration using the Stewart assay, each chromatographic fraction that might contain nanoparticles was under a mixture with both chloroform solution and ammonium ferrothiocyanate for 1 min. Then, after centrifugation at 20,000 × *g* for 1 min, the partitions from the chloroform phase were collected to measure their absorbance values at 485 nm on a UV-2600 UV-Vis Spectrophotometer (Shimadzu, MD, USA).

Accordingly, membrane ultrafiltration was performed for nanoparticle purification and collection. The above fractions containing nanoparticles were collected together and then concentrated to nearly 500 μL by centrifugation at 9600 × *g* using protein concentrator spin columns (10000 MWCO). The collected samples above were detected by BCA and SDS-PAGE analysis as described above in “Analysis of protein corona on MFlips”. Besides, nanoparticle-corona was further observed by TEM using the aforementioned protocol.

### In vivo intracellular transportation pathway observation

DiR-AFMFlip^YOYO-1^, DiR-MFlip-a^YOYO-1^, and DiR-MFlip-b^YOYO-1^ were intravenously administrated into the C57BL/6 mice (male, 3–5 weeks). After injection for 1 h and 3 h, the mice were sacrificed. The liver sections were harvested for freezing sectioning and CLSM observation. Phalloidin was used to label the cell membranes for 40 min^10^. After that, DAPI was used to stain the nuclei for ten min. After three times of careful PBS washing, the sections were imaged by CLSM.

### In vivo biodistribution study

AFMFlip, MFlip-a, and MFlip-b were labeled with DiR dye, followed by intravenous administration into the C57BL/6 mice (male, 3–5 weeks). In vivo imaging was performed using the VISQUE Invivo Smart-LF system at 1, 2, 3, 5, and 8 h post injection. Furthermore, 3 and 8 h after injection, the mice were sacrificed to collect major organs (heart, liver, spleen, lung, and kidney) and blood samples for ex vivo imaging analysis.

### In vitro luciferase gene delivery by MFlips

For luciferase gene transfection, pCDNA3.1-Luc encoding luciferase was used as a reporter plasmid. HeLa cells were seeded in a 24-well plate at a density of 5 × 10^4^ cells per well and incubated at 37 °C for 24 h. The medium was then replaced with 1 mL DMEM solution containing AFMFlip^pLuc^, and MFlip-b^pLuc^ (2 μg mL^−1^ plasmid) with or without 10% FBS. After transfection for 24 h, 48 h, and 72 h, the cells were under careful PBS washing, followed by cell lysis to collect proteins to measure the luciferase gene expression level. The total protein contents from the lysis solution were measured by BCA. After adding D-Luciferin luciferase substrate for 10 min, the luciferase expression with emitted light was detected using BioTek (SYNERGY H1MF) plate reader as the relative light unit per mg protein.

### In vivo luciferase delivery using MFlips

AFMFlip^pLuc^, MFlip-a^pLuc^, and MFlip-b^pLuc^ were intravenously administrated into the C57BL/6 mice (male, 3–5 weeks). After injection for 48 h, the mice were under intraperitoneal injection with luciferase substrate for in vivo bioluminescence imaging. Immediately following that, the mice were sacrificed, and the heart, liver, spleen, lung, and kidney were resected from each mouse for ex vivo bioluminescence imaging.

### AFMFlip-based nanomedicine for HBV infection treatment in mice

To build HBV-replication models, C57BL/6 mice (male, 3–5 weeks) received a hydrodynamic tail vein injection with 6 μg of pBB4.5–1.2× HBV in PBS solution per mouse^48,49^. After 24 h, PBS solution, AFMFlip^pCas9-gHBV^ solution (containing 6 μg pCas9-gHBV), MFlip-a^pCas9-gHBV^ solution (containing 6 μg pCas9-gHBV), and MFlip-b^pCas9-gHBV^ solution (containing 6 μg pCas9-gHBV) were injected into different mouse groups via the tail vein according to the scheme (Fig. 6a). The mouse models were then under sacrification at the endpoint of treatments. The blood samples and major organs of mice were collected and harvested for further biochemistry analysis, immunofluorescent staining, and viral load quantification. The serum samples were diluted in PBS to measure serum HBsAg, HBeAg, and HBV DNA using ELISA kits. For immunofluorescent staining of liver sections, the goat anti-HBsAg antibody (bs-1557G) was used at a dilution of 1:200, followed by the incubation and staining of Cy3-conjugated donkey anti-goat IgG H&L (catalog: #GB21404) at a dilution of 1:200. The results were visualized using an inverted fluorescence microscope (Nikon Ti2-U). The collected mouse liver tissues were lysed on ice, and the supernatant was diluted with PBS to measure HBsAg, HBeAg, and HBV DNA using ELISA kits.

DNA extraction kits were applied to purify DNA from the liver tissues. DNA fragment with CRISPR/Cas9 target location was PCR amplified using target-specific primers and purified by Tiangen gel extraction kit (Tiangen). Direct sequencing by Thermo Scientific 3730xl DNA Analyzer as well as T7E1 analysis were conducted after acquiring the amplified PCR products.

### In vivo biosafety evaluation

In vivo biosafety evaluation was performed by weight monitoring, blood biochemical test, and the H&E staining of tissues. After the HBV mouse models were sacrificed, serum levels of ALB, ALT, AST, ALP, BUN, CK, CREA, and LDH were determined using their respective activity assay kits with provided protocols. The major organs, including the heart, liver, spleen, lung, and kidney, were also collected for H&E staining to assess the biosafety of the nanoparticles.

### Statistical analysis

All the data displayed represent the results from multiple independent experiments, presented as mean ± SD unless otherwise mentioned. GraphPad Prism (GraphPad Software) was used for all of the calculations. Data comparisons were performed with two-tailed *t* test and one-way analysis of variance. A difference of *p* < 0.05 was considered statistically significant; ns: not significant (*p* > 0.05).

### Reporting summary

Further information on research design is available in the Nature Portfolio Reporting Summary linked to this article.

### Supplementary information

Supplementary Information

Reporting Summary

Peer Review File

### Source data

Source data

## Data Availability

The data that support the findings of this study are available within the article and its Supplementary Information files. The proteomics data generated in this study have been deposited in the iProx database under accession code IPX0007997000. The DNA sequencing data have been deposited in the NCBI Trace Archive database under accession code PRJNA1066418. The source data generated in this study are provided in the Source Data file. Source data are provided with this paper.
