# Supplementary material for: An antifouling membrane-fusogenic liposome for effective intracellular delivery in vivo
Source: Nat Commun. 2024 May 20;15:4267. doi: 10.1038/s41467-024-46533-z (PMC11106281; doi:10.1038/s41467-024-46533-z)
Supplement: Supplementary file 1 — Supplementary Information [file 41467_2024_46533_MOESM1_ESM.pdf]

Supplementary Information

An antifouling membrane-fusogenic liposome for effective intracellular delivery in vivo

Huimin Kong[1,†], Chunxiong Zheng[1,2,†], Ke Yi[1], Rachel L. Mintz[3], Yeh-Hsing Lao[4], Yu Tao[1], Mingqiang Li[1,*]

[1] Laboratory of Biomaterials and Translational Medicine, Center for Nanomedicine, The Third Affiliated Hospital, Sun Yat-sen University, Guangzhou 510630, China

[2] School of Chemistry, South China Normal University, Guangzhou 510006, China

[3] Department of Biomedical Engineering, Washington University in St. Louis, St. Louis, MO 63110, USA

[4] Department of Pharmaceutical Sciences, University at Buffalo, The State University of New York, Buffalo, NY 14214, USA

[†] These authors contributed equally: Huimin Kong and Chunxiong Zheng

*E-mail: limq567@mail.sysu.edu.cn

Contents

Supplementary Fig. 1. Optimization of AFMFlip with serum-resistance membrane-fusion capacity.

Supplementary Fig. 2. Size distribution and zeta potential of the optimized AFMFlip.

Supplementary Fig. 3. Protein corona quantification with bicinchoninic acid (BCA) assay in the presence of 10% FBS or 10% mouse serum.

Supplementary Fig. 4. Quality evaluation of liquid chromatography with tandem mass spectrometry (LC-MS/MS) analysis results.

Supplementary Fig. 5. Total relative raw abundances of proteins on AFMFlip, MFlip-a, and MFlip-b after 10% FBS incubation.

Supplementary Fig. 6. Heat map of the top-most abundant proteins on AFMFlip after incubation with 10% FBS.

Supplementary Fig. 7. Heat map of the top-most abundant proteins on MFlip-a after incubation with 10% FBS.

Supplementary Fig. 8. Heat map of the top-most abundant proteins on MFlip-b after incubation with 10% FBS.

Supplementary Fig. 9. The relative protein content of the corona proteins identified on AFMFlip, MFlip-1, and MFlip-b by LC-MS/MS after interaction with 10% FBS according to their calculated isoelectric point (pI).

Supplementary Fig. 10. The relative protein content of the corona proteins identified on AFMFlip, MFlip-1, and MFlip-b by LC-MS/MS after interaction with 10% FBS according to their molecular weight.

Supplementary Fig. 11. Structural characterization and plasmid-loading capacity of AFMFlip, MFlip-a, and MFlip-b.

Supplementary Fig. 12. Membrane-fusion performance of AFMFlip, MFlip-a, and MFlip-b in the medium with or without 10% FBS.

Supplementary Fig. 13. The cellular uptake of AFMFlip, MFlip-a, and MFlip-b by HeLa cells in the absence or presence of serum for 2 h under the following conditions: 50 μg mL^{-1} Z-Phe-Phe-Phe-OH (membrane fusion inhibitor), 10 μg mL^{-1} chlorpromazine (clathrin-mediated endocytosis inhibitor); 100 μg mL^{-1} amiloride (micropinocytosis inhibitor); 15 μg mL^{-1} nystatin (caveolae-mediated endocytosis inhibitor), or dual-inhibitor treatments.

Supplementary Fig. 14. Confocal laser scanning microscopy (CLSM) images of HeLa cells incubated with AFMFlip^{YOYO-1}, MFlip-a^{YOYO-1}, and MFlip-b^{YOYO-1} under the medium containing 10% FBS.

Supplementary Fig. 15. The photograph of recovery plasma solutions after separation by size exclusion chromatography.

Supplementary Fig. 16. DLS measurements of chromatographic fractions (1 mL) separated from the plasma of mice treated with DiR-AFMFlip^{YOYO-1}, DiR-MFlip-a^{YOYO-1}, and DiR-MFlip-b^{YOYO-1} at 1 h and 3 h post-injection.

Supplementary Fig. 17. The established standard curve and regression equation from the Stewart assay.

Supplementary Fig. 18. The lipid concentration in the chromatographic fractions from numbers 7 to 16 for each group.

Supplementary Fig. 19. The lipid determination of concentrated chromatographic fractions containing lipid nanoparticles by membrane ultrafiltration.

Supplementary Fig. 20. CLSM images of hepatocytes in the liver tissues from mice treated with DiR-AFMFlip^{YOYO-1}, DiR-MFlip-a^{YOYO-1}, and DiR-MFlip-b^{YOYO-1} for 1 h post-injection.

Supplementary Fig. 21. In vivo distribution of AFMFlip, MFlip-a, and MFlip-b.

Supplementary Fig. 22. Plasmid profile of pCas9-gHBV encoded with the Cas9 protein and gRNA targeting the HBV genome among open reading frameworks of polymerase- (P) and X protein (X).

Supplementary Fig. 23. The detection of mice's blood biochemistry indexes and weight changes in different groups. The biochemical blood indexes included albumin (ALB), alanine aminotransferase (ALT), aspartate aminotransferase (AST), alkaline phosphatase (ALP), blood urea nitrogen (BUN), creatine kinase (CK), creatinine (CREA), and lactate dehydrogenase (LDH).

Supplementary Fig. 24. Hematoxylin and eosin (H&E) staining of mouse tissues from the major organs (heart, liver, spleen, lung, and kidney) after the injection with PBS, AFMFlip$^{pCas9-gHBV}$, MFlip-a$^{pCas9-gHBV}$, or MFlip-b$^{pCas9-gHBV}$.

Supplementary Fig. 25. Tracking of indels by decomposition (TIDE) analysis of the editing efficacy in the HBV-targeting site after treatment with PBS, MFlip-a$^{pCas9-gHBV}$, and MFlip-b$^{pCas9-gHBV}$.

Supplementary Table 1. The five AFMFlip systems (NP-1 to NP-5) formulated with different molar ratios of lipid components.

Supplementary Table 2. Raw abundance (log10) of the top 20 most-abundant proteins identified in the coronas of AFMFlip, MFlip-a, and MFlip-b after incubation with FBS.

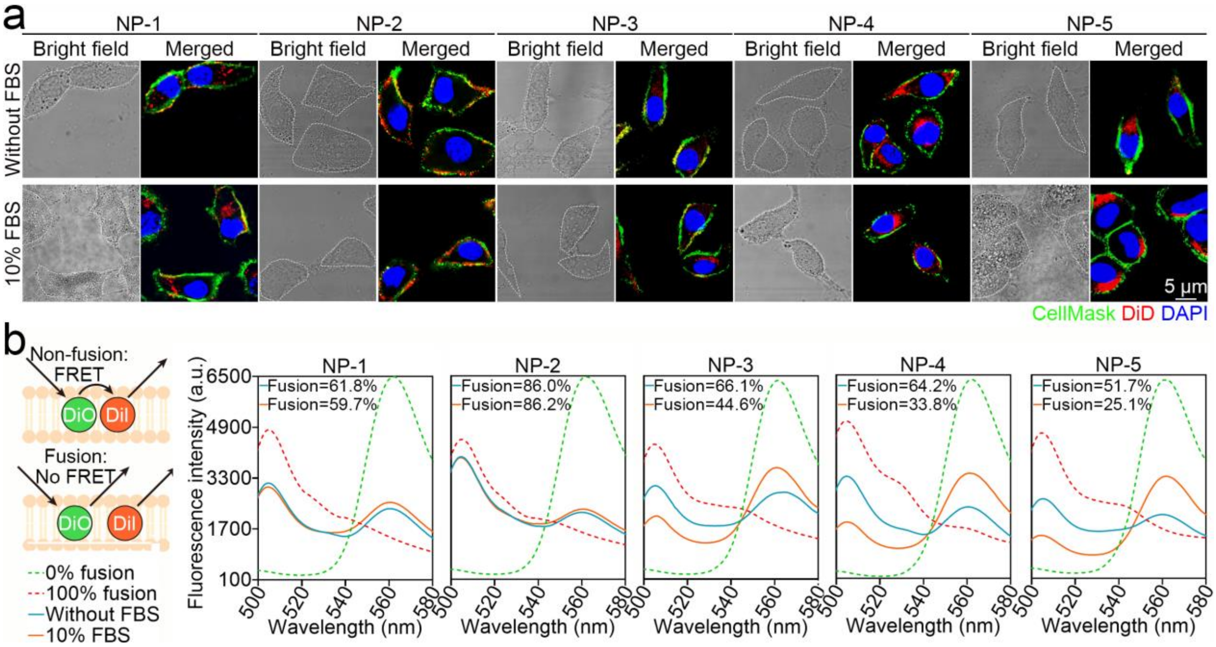

Supplementary Fig. 1. Optimization of AFMFlip with serum-resistance membrane-fusion capacity. (**a**) CLSM images of HeLa cells with bright field to observe the cellular uptake of liposomes (NP-1 to NP-5 with different lipid component ratios) in the medium with or without 10% FBS. Liposomes were labeled with 1,1'-dioctadecyl-3,3,3',3'-tetramethylindodicarbocyanine,4-chlorobenzenesulfonate salt (DiD). The nuclei were stained with DAPI and the cell membranes were labeled with CellMask. The merged images are also presented in the main text (Fig. 2b). Experiment was repeated three times independently with similar results. (**b**) FRET analysis to evaluate the membrane-fusion efficacy after 1 h incubation with cells in the medium with or without 10% FBS at 37 °C.

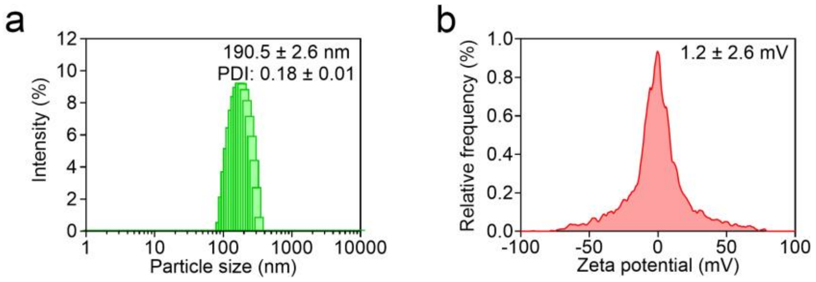

Supplementary Fig. 2. Size distribution and zeta potential of the optimized AFMFlip. (**a**) The dynamic light scattering (DLS) measurement of size distribution of the optimized AFMFlip. (**b**) The analysis of zeta potential distribution of the optimized AFMFlip. Data are presented as mean ± SD (n = 3 biologically independent samples).

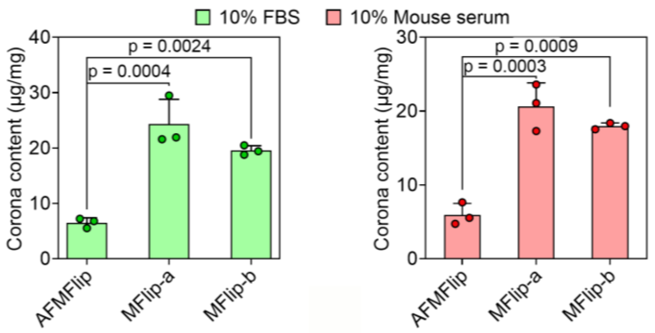

Supplementary Fig. 3. Protein corona quantification with bicinchoninic acid (BCA) assay in the presence of 10% FBS or 10% mouse serum. Data are presented as mean ± SD and statistically analyzed using one-way ANOVA (n = 3 biologically independent samples).

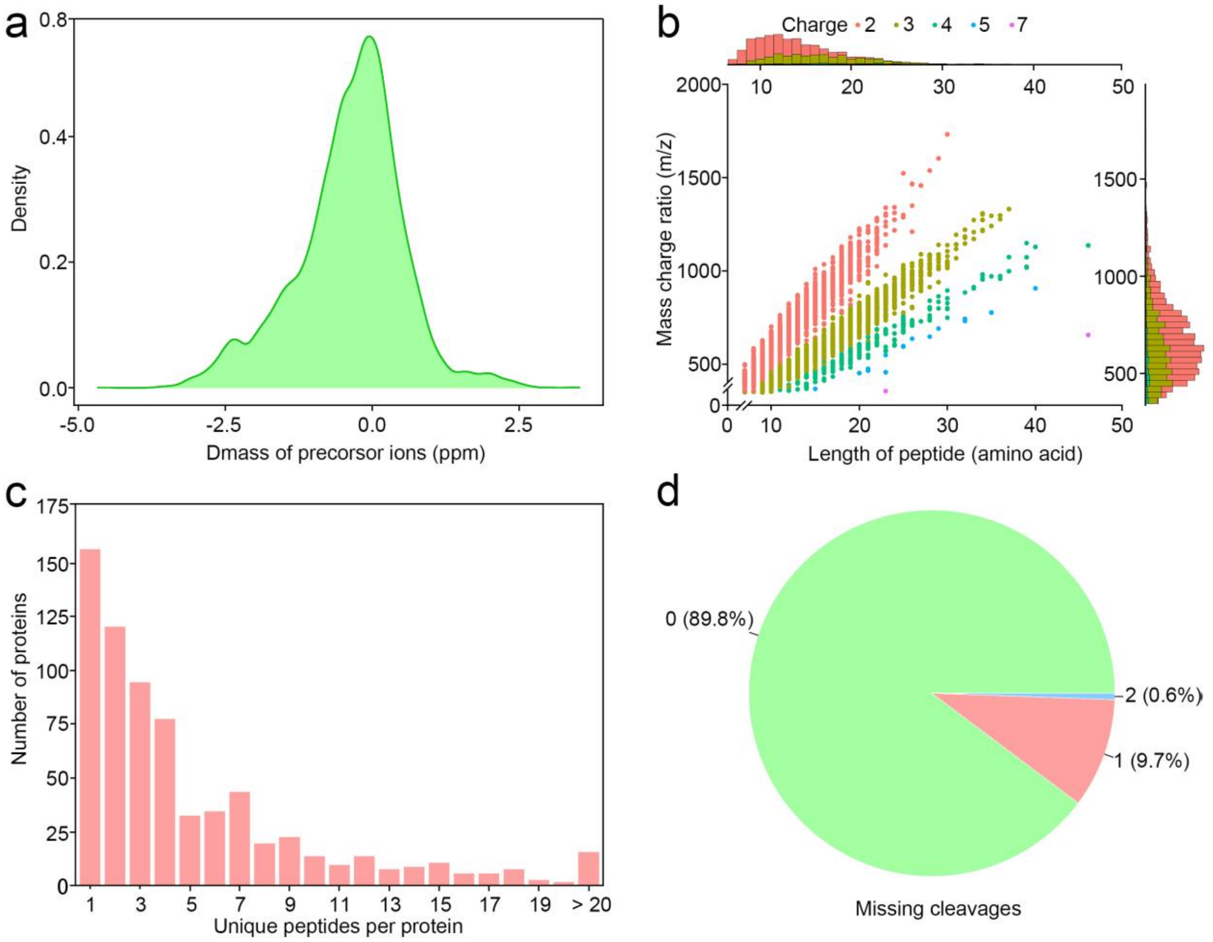

Supplementary Fig. 4. Quality evaluation of liquid chromatography with tandem mass spectrometry (LC-MS/MS) analysis results. (**a**) The distribution map of mass deviation. The x-axis is the mass deviation of precursor ions, while the y-axis is the corresponding density of precursor ions. (**b**) The distribution map of peptide length. (**c**) The distribution map of unique peptides. (**d**) The distribution map of missing cleavages.

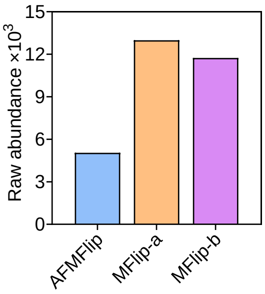

Supplementary Fig. 5. Total relative raw abundances of proteins on AFMFlip, MFlip-a, and MFlip-b after 10% FBS incubation. The inbucation was conducted for 1 h at 37 °C. The raw abundance of top 20 proteins is presented in Supplementary Table 2.

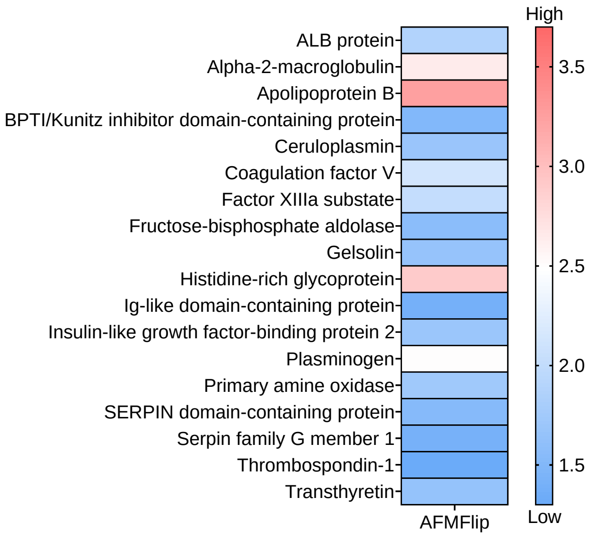

Supplementary Fig. 6. Heat map of the top-most abundant proteins on AFMFlip after incubation with 10% FBS. The inbucation was conducted for 1 h at 37 °C. The raw abundance of top 20 most-abundant proteins identified in the coronas of AFMFlip is presented in Supplementary Table 2.

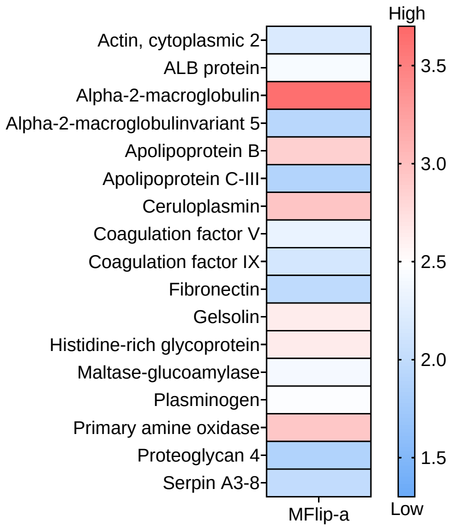

Supplementary Fig. 7. Heat map of the top-most abundant proteins on MFlip-a after incubation with 10% FBS. The inbucation was conducted for 1 h at 37 °C. The raw abundance of top 20 most-abundant proteins identified in the coronas of MFlip-a is presented in Supplementary Table 2.

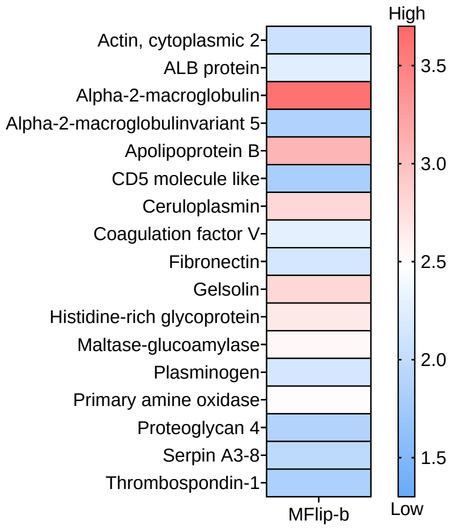

Supplementary Fig. 8. Heat map of the top-most abundant proteins on MFlip-b after incubation with 10% FBS. The inbucation was conducted for 1 h at 37 °C. The raw abundance of top 20 most-abundant proteins identified in the coronas of MFlip-b is presented in Supplementary Table 2.

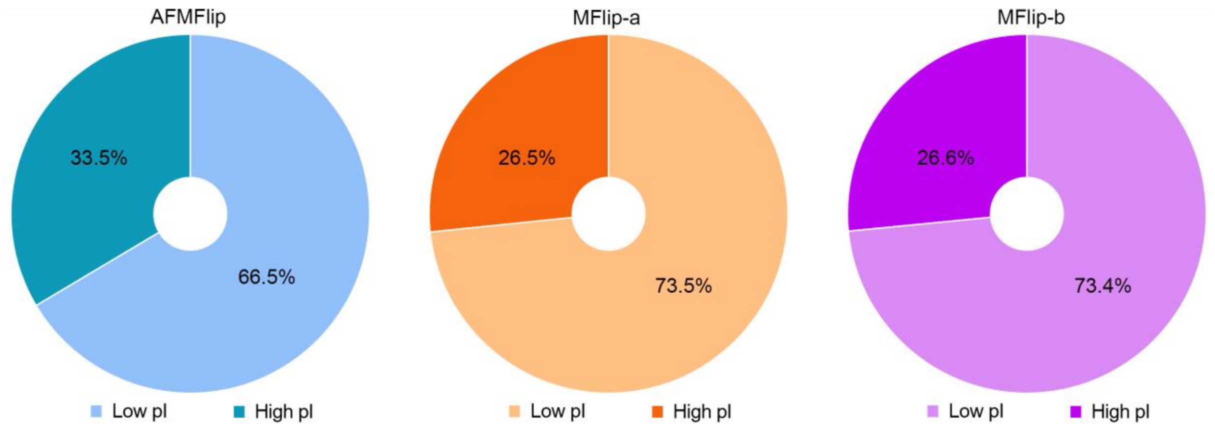

Supplementary Fig. 9. The relative protein content of the corona proteins identified on AFMFlip, MFlip-1, and MFlip-b by LC-MS/MS after interaction with 10% FBS according to their calculated isoelectric point (pI). The pI values are defined as follows: Low (4-7) and high (>7).

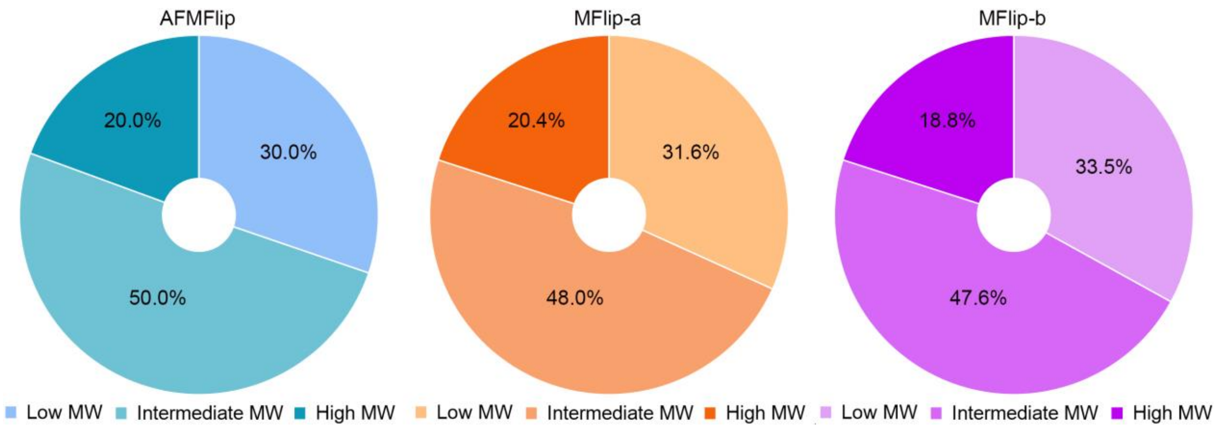

Supplementary Fig. 10. The relative protein content of the corona proteins identified on AFMFlip, MFlip-1, and MFlip-b by LC-MS/MS after interaction with 10% FBS according to their molecular weight. Molecular weights (kDa) are defined as follows: Low (<30), intermediate (30-90), and high (>90).

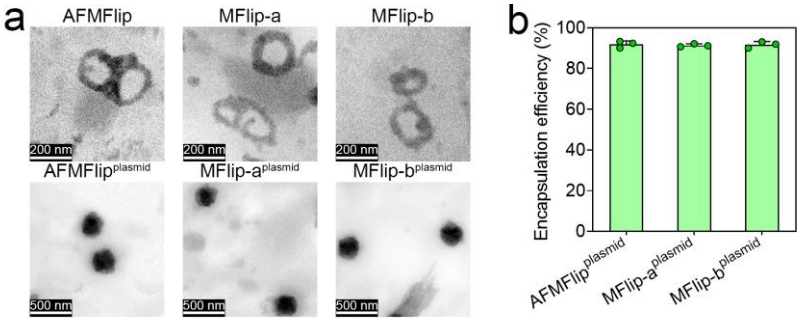

Supplementary Fig. 11. Structural characterization and plasmid-loading capacity of AFMFlip, MFlip-a, and MFlip-b. (**a**) TEM images of AFMFlip, MFlip-a, and MFlip-b with and without the encapsulation of $CaCO_3$/plasmid nanoparticles. Experiment was repeated three times independently with similar results. (**b**) Plasmid encapsulation efficiencies of the AFMFlip, MFlip-a, and MFlip-b. Data are presented as mean ± SD (n = 3 biologically independent samples).

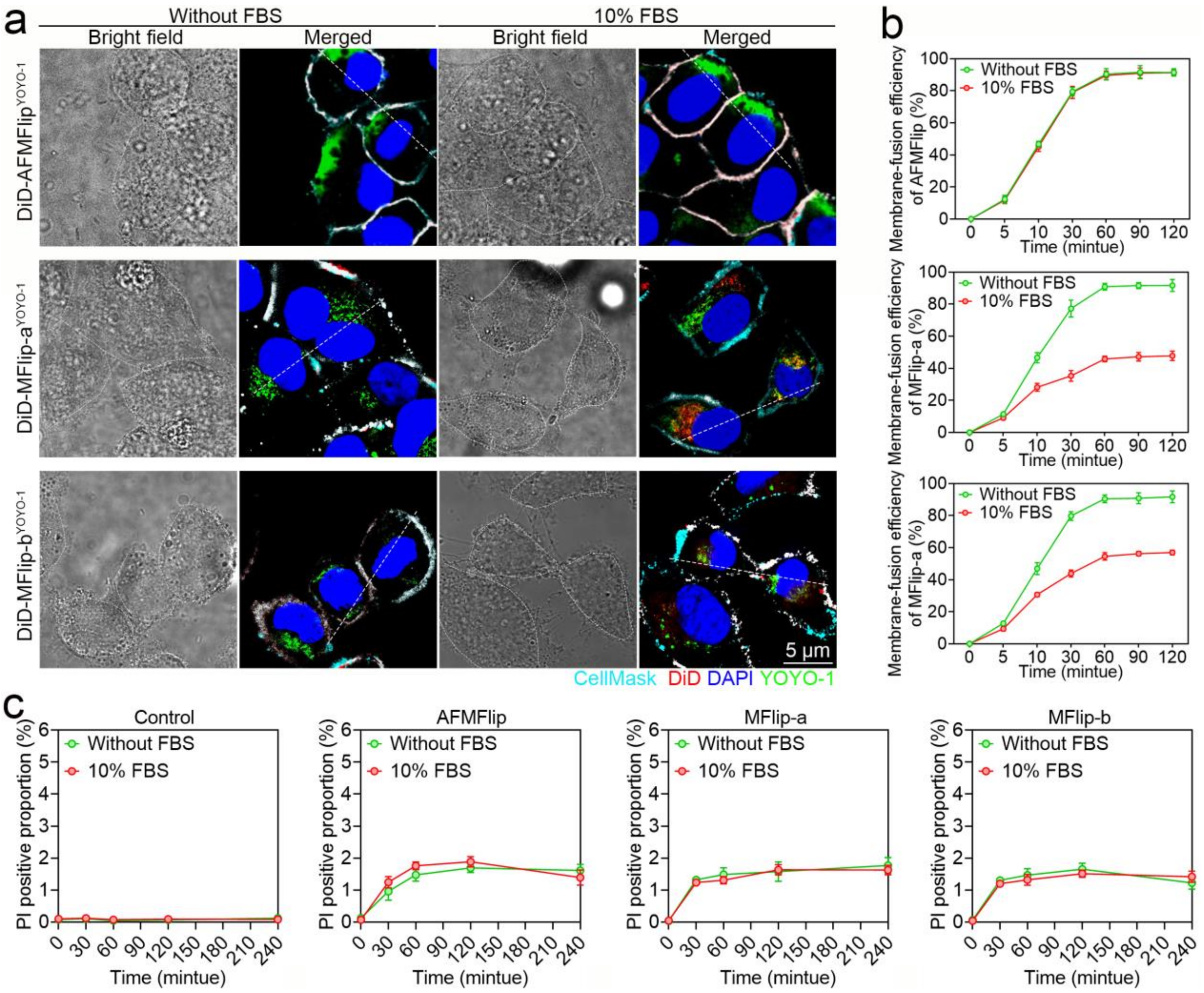

Supplementary Fig. 12. Membrane-fusion performance of AFMFlip, MFlip-a, and MFlip-b in the medium with or without 10% FBS. (**a**) CLSM images of HeLa cells with bright field to observe the cellular uptake of DiD-AFMFlip^{YOYO-1}, DiD-MFlip-a^{YOYO-1}, and DiD-MFlip-b^{YOYO-1} in the medium with or without 10% FBS. Liposomes were labeled with DiD. The nuclei were stained with DAPI and the cell membranes were labeled with CellMask. The merged images are also presented in the main text (Fig. 3a). Experiment was repeated three times independently with similar results. (**b**) Time-dependent membrane-fusion performance based on FRET analysis of AFMFlip, MFlip-a, and MFlip-b after cell incubation without or with 10% FBS. Data are presented as mean ± SD (n = 3 biologically independent samples). (**c**) Quantification of propidium iodide (PI)-positive cells after treatment with AFMFlip, MFlip-a, and MFlip-b under the medium without or with 10% FBS at 37 °C. Data are presented as mean ± SD (n = 3 biologically independent samples).

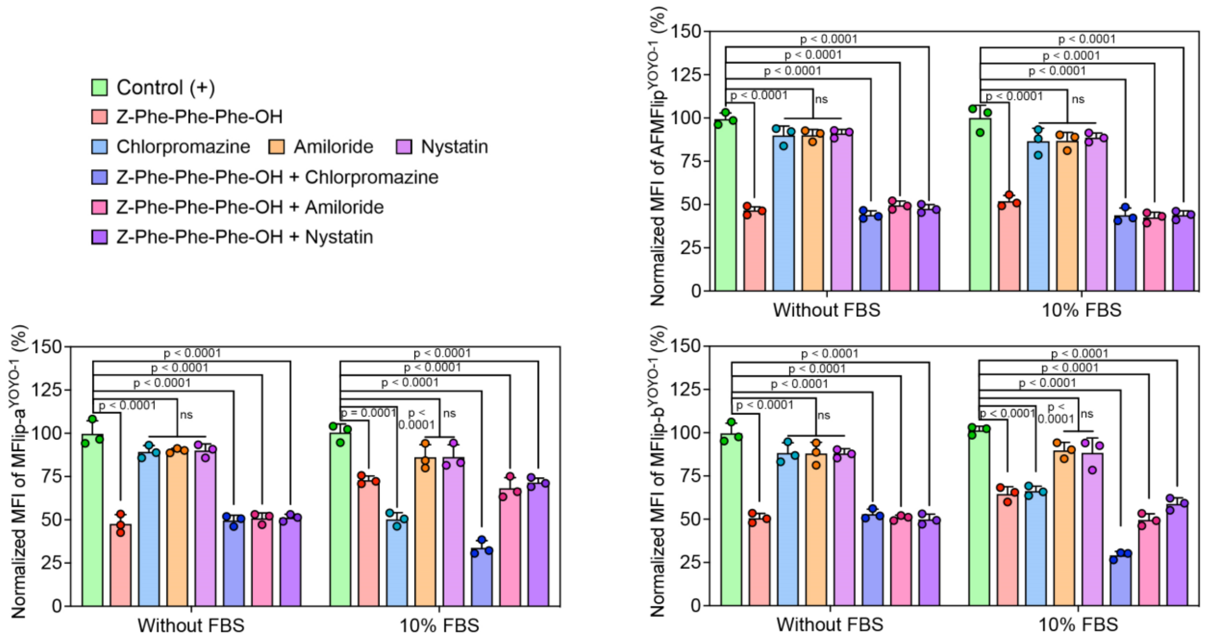

Supplementary Fig. 13. The cellular uptake of AFMFlip, MFlip-a, and MFlip-b by HeLa cells in the absence or presence of serum for 2 h under the following conditions: 50 μg mL^{-1} Z-Phe-Phe-Phe-OH (membrane fusion inhibitor), 10 μg mL^{-1} chlorpromazine (clathrin-mediated endocytosis inhibitor); 100 μg mL^{-1} amiloride (micropinocytosis inhibitor); 15 μg mL^{-1} nystatin (caveolae-mediated endocytosis inhibitor), or dual-inhibitor treatments. Data are presented as mean ± SD and statistically analyzed using one-way ANOVA (n = 3 biologically independent samples). ns: no significance ($p > 0.05$).

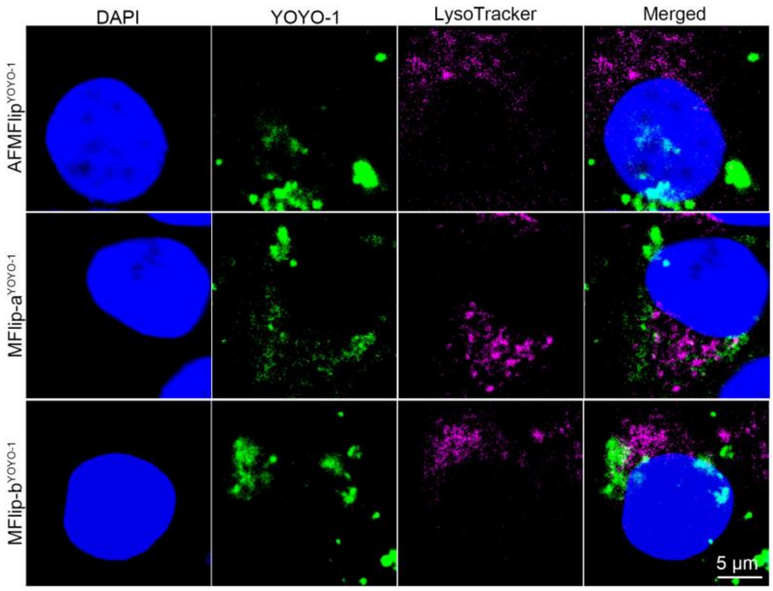

Supplementary Fig. 14. Confocal laser scanning microscopy (CLSM) images of HeLa cells incubated with AFMFlip$^{\mathbf{YOYO\text{-}1}}$, MFlip-a$^{\mathbf{YOYO\text{-}1}}$, and MFlip-b$^{\mathbf{YOYO\text{-}1}}$ under the medium containing 10% FBS. Cellular nuclei were stained with DAPI and lysosomes were stained with LysoTracker. Experiment was repeated three times independently with similar results.

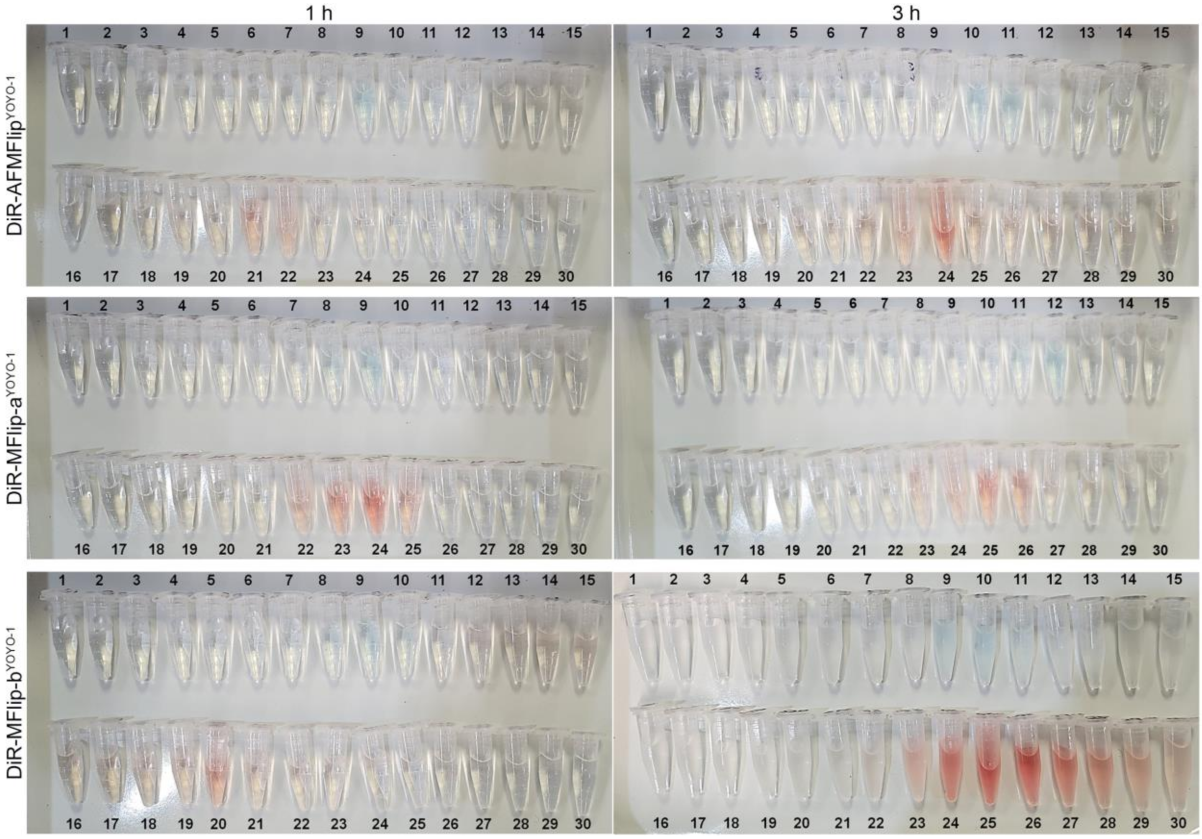

Supplementary Fig. 15. The photograph of recovery plasma solutions after separation by size exclusion chromatography. The plasma was collected from mice treated with DiR-AFMFlip^{YOYO-1}, DiR-MFlip-a^{YOYO-1}, and DiR-MFlip-b^{YOYO-1} at 1 h and 3 h after in vivo circulation. The chromatographic fractions from number 7 to 16 were selected for the next Stewart assay. Experiment was repeated three times independently with similar results.

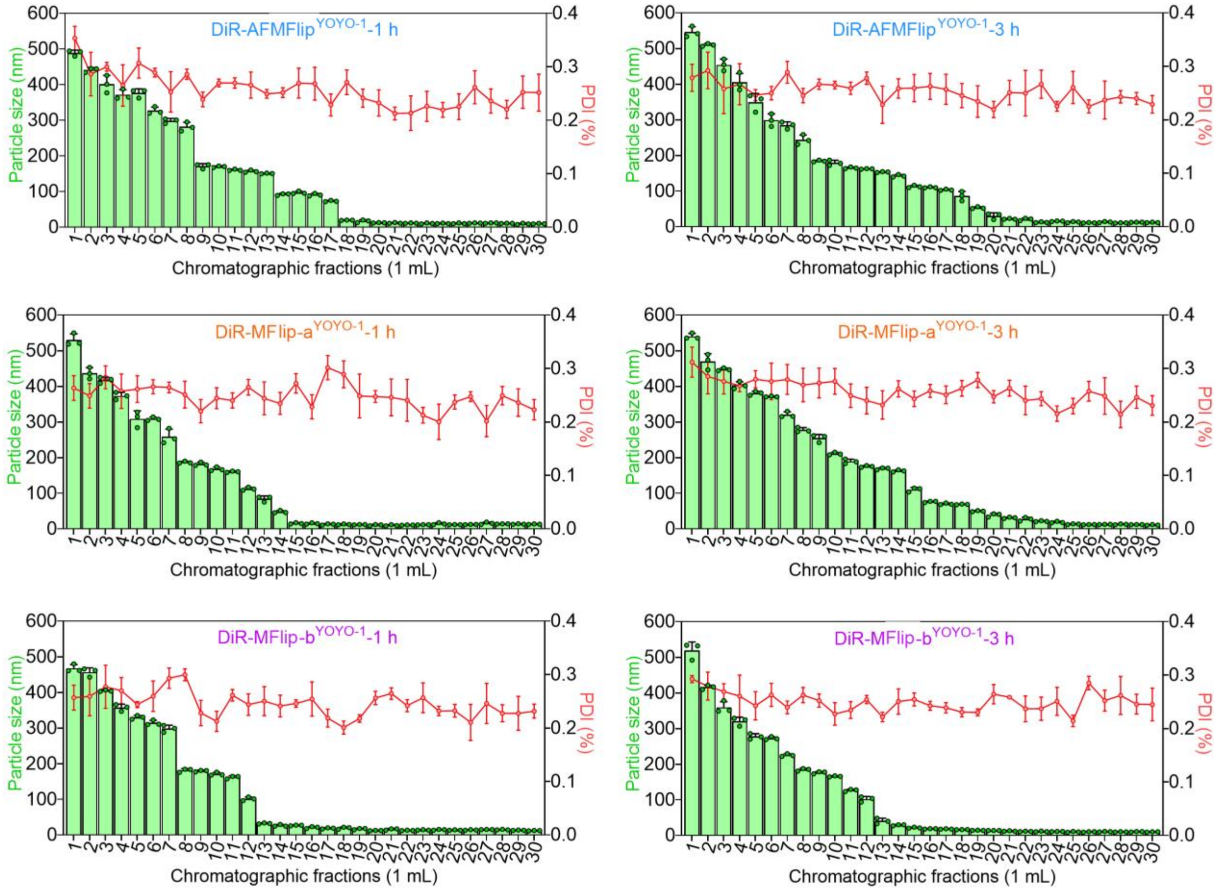

Supplementary Fig. 16. DLS measurements of chromatographic fractions (1 mL) separated from the plasma of mice treated with DiR-AFMFlip$^{YOYO\text{-}1}$, DiR-MFlip-a$^{YOYO\text{-}1}$, and DiR-MFlip-b$^{YOYO\text{-}1}$ at 1 h and 3 h post-injection. The chromatographic fractions from numbers 7 to 16 were selected for the next Stewart assay. Data are presented as mean ± SD (n = 3 biologically independent samples).

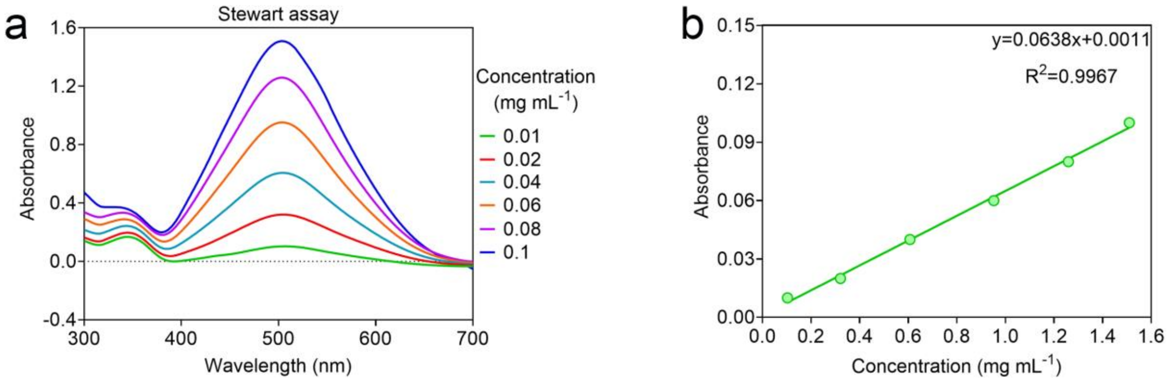

Supplementary Fig. 17. The established standard curve and regression equation from the Stewart assay. This Stewart assay is applied for the next lipid qualification. Experiment was repeated three times independently with similar results.

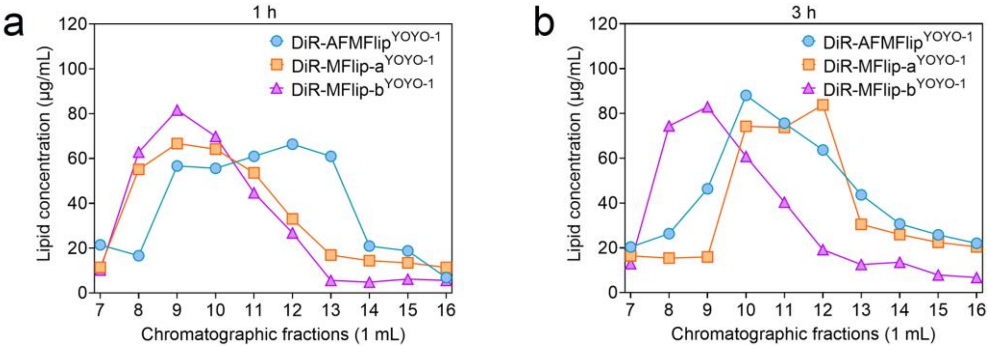

Supplementary Fig. 18. The lipid concentration in the chromatographic fractions from numbers 7 to 16 for each group. The calculated lipid concentrations were determined by the Stewart assay based on Supplementary Fig. 18. Experiment was repeated three times independently with similar results.

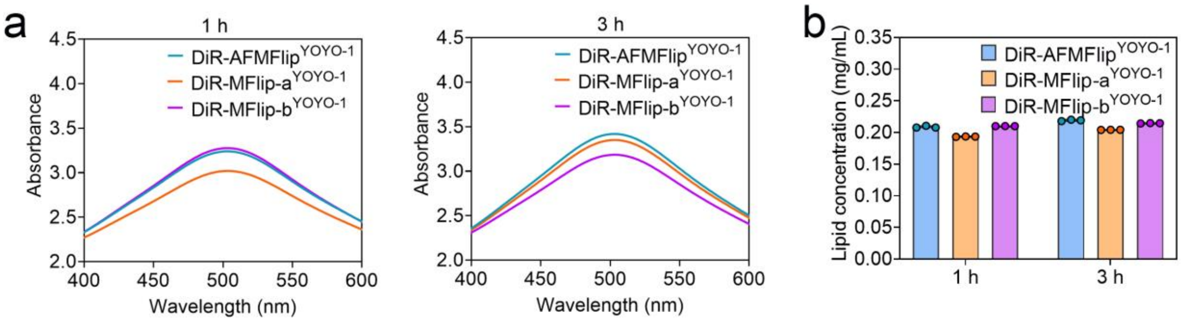

Supplementary Fig. 19. The lipid determination of concentrated chromatographic fractions containing lipid nanoparticles by membrane ultrafiltration. (**a**) Spectrum analysis of the concentrated chromatographic fractions for each group. Experiment was repeated three times independently with similar results. (**b**) Quantification of the concentrated chromatographic fractions for each group. Data are presented as mean ± SD (n = 3 biologically independent samples).

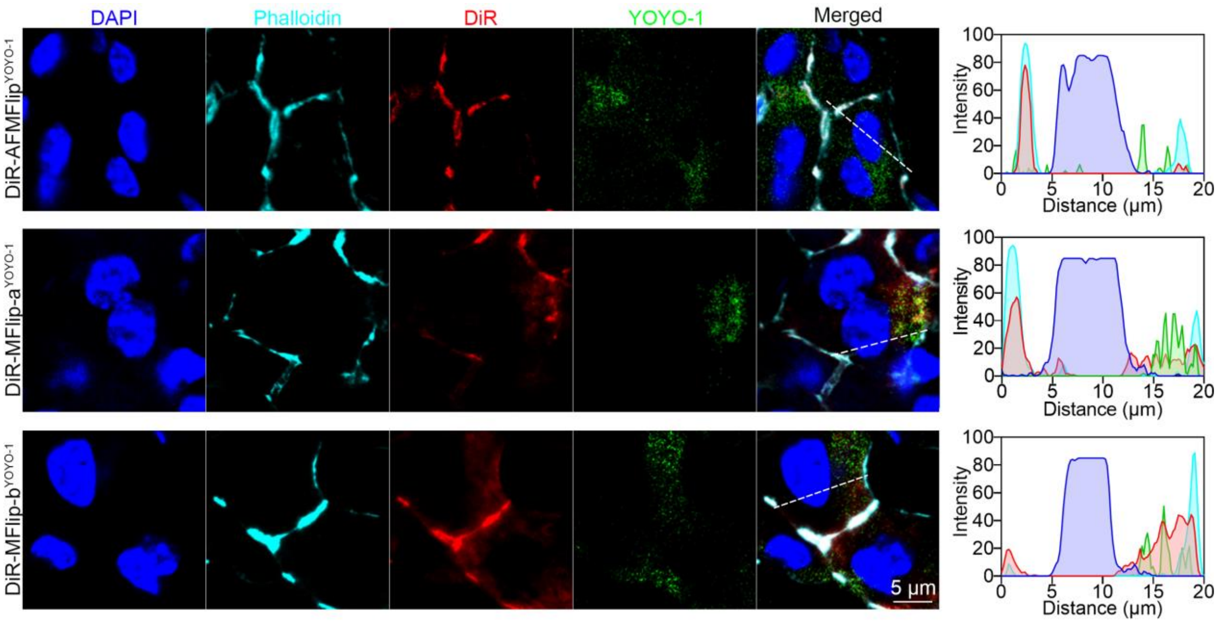

Supplementary Fig. 20. CLSM images of hepatocytes in the liver tissues from mice treated with DiR-AFMFlip^{YOYO-1}, DiR-MFlip-a^{YOYO-1}, and DiR-MFlip-b^{YOYO-1} for 1 h post-injection. The nuclei were stained with DAPI, and the cell membranes were labeled with Phalloidin. Co-localization profiles are shown on the right side and were analyzed along the white lines by ImageJ software. Experiment was repeated three times independently with similar results.

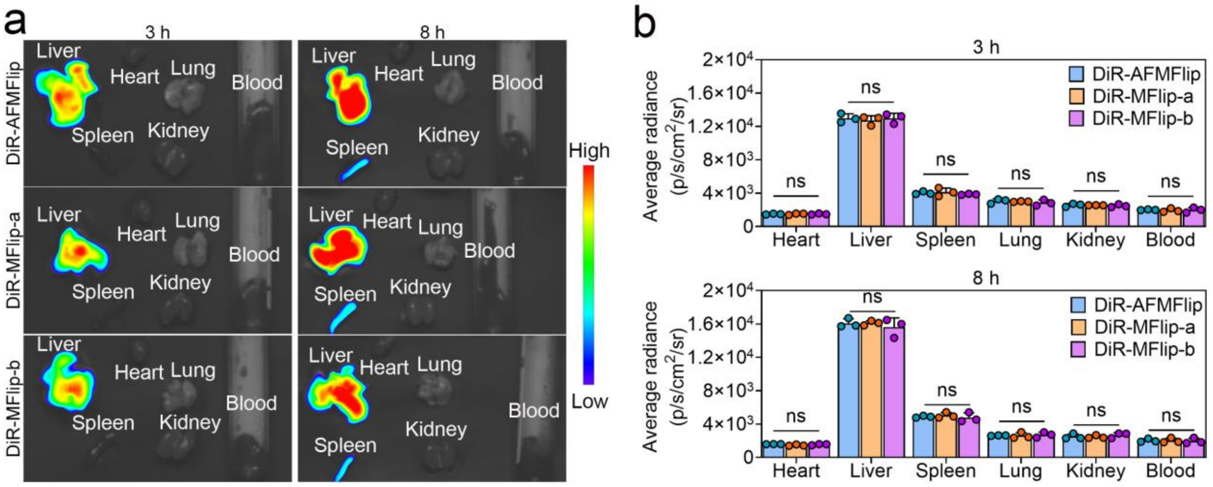

Supplementary Fig. 21. In vivo distribution of AFMFlip, MFlip-a, and MFlip-b. (**a**) Representative ex vivo images of distribution. (**b**) Corresponding quantification analysis in major organs (heart, liver, spleen, lung, and kidney) from mice at 3 h and 8 h post-injection of DiR-AFMFlip, DiR-MFlip-a, and DiR-MFlip-b. Data are presented as mean ± SD and statistically analyzed using one-way ANOVA (n = 3 biologically independent animals). ns: no significance ($p > 0.05$).

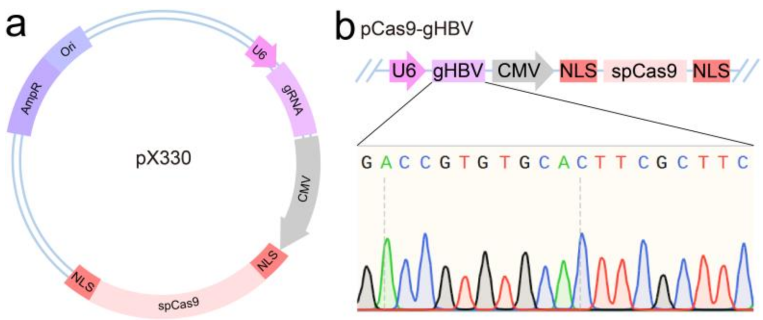

Supplementary Fig. 22. Plasmid profile of pCas9-gHBV encoded with the Cas9 protein and gRNA targeting the HBV genome among open reading frameworks of polymerase- (P) and X protein (X). (**a**) Schematic structure of pX330 vector as the CRISPR/Cas9 plasmid. (**b**) Sequencing result of gRNA targeting the HBV genome, which was inserted into the pX330 to establish pCas9-gHBV.

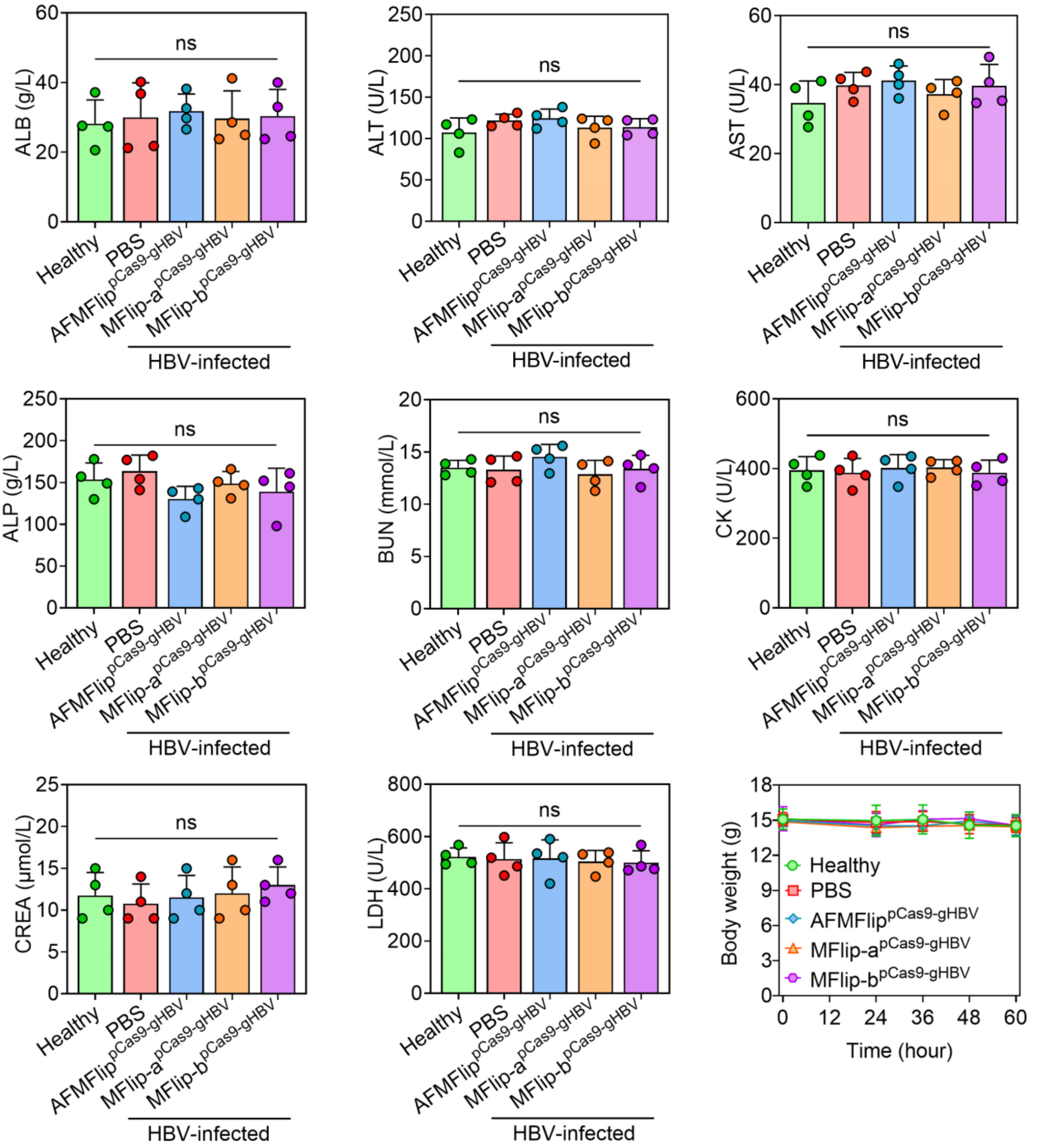

Supplementary Fig. 23. The detection of mice's blood biochemistry indexes and weight changes in different groups. The biochemical blood indexes included albumin (ALB), alanine aminotransferase (ALT), aspartate aminotransferase (AST), alkaline phosphatase (ALP), blood urea nitrogen (BUN), creatine kinase (CK), creatinine (CREA), and lactate dehydrogenase (LDH). Data are presented as mean ± SD and statistically analyzed using one-way ANOVA (n = 4 biologically independent animals). ns: no significance ($p > 0.05$).

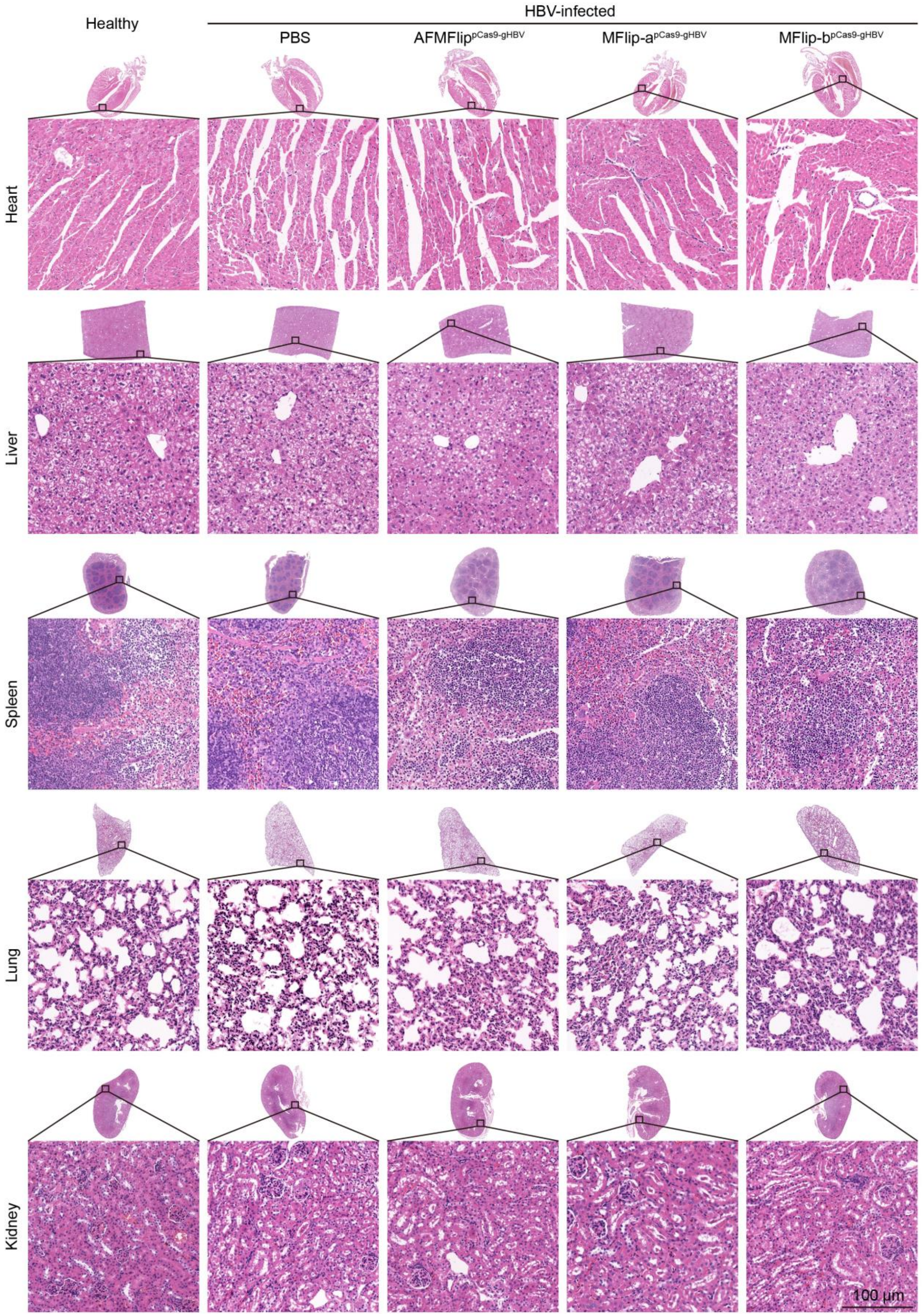

Supplementary Fig. 24. Hematoxylin and eosin (H&E) staining of mouse tissues from the major organs (heart, liver, spleen, lung, and kidney) after the injection with PBS,

AFMFlip$^{pCas9\text{-}gHBV}$, MFlip-a$^{pCas9\text{-}gHBV}$, or MFlip-b$^{pCas9\text{-}gHBV}$. Experiment was repeated three times independently with similar results.

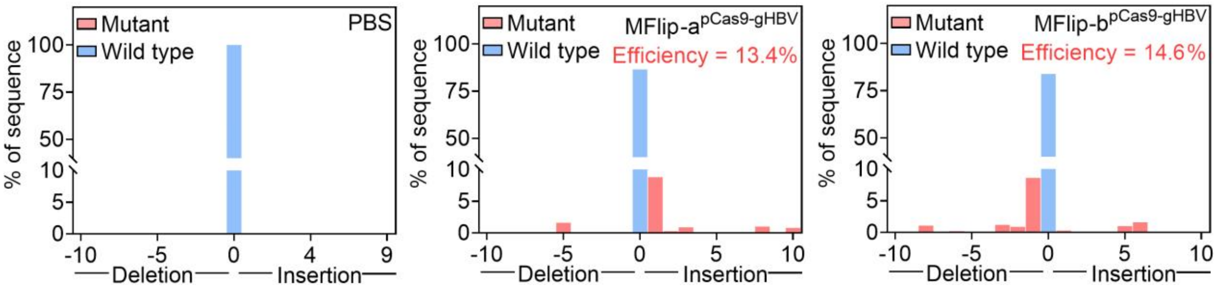

Supplementary Fig. 25. Tracking of indels by decomposition (TIDE) analysis of the editing efficacy in the HBV-targeting site after treatment with PBS, MFlip-a$^{pCas9-gHBV}$, and MFlip-b$^{pCas9-gHBV}$. The editing efficiency for TIDE analysis was calculated online (https://tide.nki.nl/). Experiment was repeated three times independently with similar results.

Supplementary Table 1. The five AFMFlip systems (NP-1 to NP-5) formulated with different molar ratios of lipid components.

	**Lipids (molar ratio）**	**Size (nm)**	**PDI**	**Zeta potential (mV)**
NP-1	DSPE-PEG/DOPC/DOTAP (5/95/0)	187.4 ± 3.4	0.15 ± 0.01	-7.5 ± 1.3
NP-2	DSPE-PEG/DOPC/DOTAP (5/90/5)	190.5 ± 2.6	0.18 ± 0.01	1.2 ± 2.6
NP-3	DSPE-PEG/DOPC/DOTAP (5/85/10)	193.0 ± 1.3	0.20 ± 0.01	3.7 ± 1.7
NP-4	DSPE-PEG/DOPC/DOTAP (5/75/20)	199.5 ± 3.0	0.13 ± 0.02	11.2 ± 2.3
NP-5	DSPE-PEG/DOPC/DOTAP (5/65/30)	185.9 ± 2.4	0.22 ± 0.01	19.2 ± 1.2

Supplementary Table 2. Raw abundance (log10) of the top 20 most-abundant proteins identified in the coronas of AFMFlip, MFlip-a, and MFlip-b after incubation with FBS.

No.	AFMFlip		MFlip-a		MFlip-b	
1	Apolipoprotein B	3.26	Alpha-2-macroglobulin	3.65	Alpha-2-macroglobulin	3.63
2	Histidine-rich glycoprotein	2.91	Ceruloplasmin	2.96	Apolipoprotein B	3.09
3	Alpha-2-macroglobulin	2.66	Primary amine oxidase	2.94	Gelsolin	2.81
4	Plasminogen	2.51	Apolipoprotein B	2.87	Ceruloplasmin	2.81
5	Coagulation factor V	2.14	Histidine-rich glycoprotein	2.65	Histidine-rich glycoprotein	2.67
6	Factor XIIIa substate	2.03	Gelsolin	2.64	Maltase-glucoamylase	2.55
7	ALB protein	1.89	Uncharacterized protein	2.53	Primary amine oxidase	2.52
8	Uncharacterized protein	1.80	Uncharacterized protein	2.48	Uncharacterized protein	2.44
9	Primary amine oxidase	1.75	Plasminogen	2.47	Uncharacterized protein	2.42
10	Insulin-like growth factor-binding protein 2	1.71	ALB protein	2.43	Coagulation factor V	2.29
11	Ceruloplasmin	1.70	Maltase-glucoamylase	2.42	ALB protein	2.26
12	Gelsolin	1.67	Coagulation factor V	2.33	Plasminogen	2.16
13	Transthyretin	1.66	Actin, cytoplasmic 2	2.21	Fibronectin	2.16
14	Uncharacterized protein	1.59	Coagulation factor IX	2.16	Uncharacterized protein	2.16
15	Fructose-bisphosphate aldolase	1.58	Serpin A3-8	2.02	Actin, cytoplasmic 2	2.09
16	SERPIN domain-containing protein	1.54	Fibronectin	1.99	Serpin A3-8	1.98

No.	AFMFlip		MFlip-a		MFlip-b	
17	BPTI/Kunitz inhibitor domain-containing protein	1.51	Alpha-2-macroglobulin variant 5	1.94	Proteoglycan 4	1.89
18	Serpin family G member 1	1.42	Apolipoprotein C-III	1.90	Alpha-2-macroglobulin	1.87
19	Ig-like domain-containing protein	1.41	Uncharacterized protein	1.89	Thrombospondin-1	1.85
20	Thrombospondin-1	1.33	Proteoglycan 4	1.88	CD5 molecule like	1.84
