# Supplementary material for: An antifouling membrane-fusogenic liposome for effective intracellular delivery in vivo
Source: Nat Commun. 2024 May 20;15:4267. doi: 10.1038/s41467-024-46533-z (PMC11106281; doi:10.1038/s41467-024-46533-z)
Supplement: Supplementary file 2 — Reporting Summary [file 41467_2024_46533_MOESM2_ESM.pdf]

nature portfolio

Corresponding author(s): Mingqiang Li

Last updated by author(s): Jan 22, 2024

Reporting Summary

Nature Portfolio wishes to improve the reproducibility of the work that we publish. This form provides structure for consistency and transparency in reporting. For further information on Nature Portfolio policies, see our Editorial Policies and the Editorial Policy Checklist.

Statistics

For all statistical analyses, confirm that the following items are present in the figure legend, table legend, main text, or Methods section.

n/a	Confirmed	
☐	☒	The exact sample size (*n*) for each experimental group/condition, given as a discrete number and unit of measurement
☐	☒	A statement on whether measurements were taken from distinct samples or whether the same sample was measured repeatedly
☐	☒	The statistical test(s) used AND whether they are one- or two-sided<br>*Only common tests should be described solely by name; describe more complex techniques in the Methods section.*
☒	☐	A description of all covariates tested
☒	☐	A description of any assumptions or corrections, such as tests of normality and adjustment for multiple comparisons
☐	☒	A full description of the statistical parameters including central tendency (e.g. means) or other basic estimates (e.g. regression coefficient) AND variation (e.g. standard deviation) or associated estimates of uncertainty (e.g. confidence intervals)
☐	☒	For null hypothesis testing, the test statistic (e.g. *F*, *t*, *r*) with confidence intervals, effect sizes, degrees of freedom and *P* value noted<br>*Give P values as exact values whenever suitable.*
☒	☐	For Bayesian analysis, information on the choice of priors and Markov chain Monte Carlo settings
☒	☐	For hierarchical and complex designs, identification of the appropriate level for tests and full reporting of outcomes
☐	☒	Estimates of effect sizes (e.g. Cohen's *d*, Pearson's *r*), indicating how they were calculated

Our web collection on statistics for biologists contains articles on many of the points above.

Software and code

Policy information about availability of computer code

Data collection	Anton Paar Litesizer 500 particle analyzer, FEI Tecnai G2 F30 TEM microscope, Leica STELLARIS STED confocal microscope, Thermo Scientific Q Exactive mass spectrometer, Nikon Ti2-U inverted fluorescence microscope, BD FACS Calibur flow cytometer, Tecan Spark spectrofluorometer, Shimadzu UV-2600 UV-Vis Spectrophotometer, BioTek (SYNERGY H1MF) plate reader, VISQUE InVivo Smart-LF Imaging and Analysis system, Thermo Scientific 3730xl DNA Analyzer.
Data analysis	All the quantitative data were expressed as mean ± SD and analyzed with GraphPad Prism (v.9.0) software. GraphPad Prism (v.9.0), CytExpert (2.3) and Image J (v1.8.0) were used for statistical analysis and figure production.

For manuscripts utilizing custom algorithms or software that are central to the research but not yet described in published literature, software must be made available to editors and reviewers. We strongly encourage code deposition in a community repository (e.g. GitHub). See the Nature Portfolio guidelines for submitting code & software for further information.

Data

Policy information about availability of data

All manuscripts must include a data availability statement. This statement should provide the following information, where applicable:

- Accession codes, unique identifiers, or web links for publicly available datasets
- A description of any restrictions on data availability
- For clinical datasets or third party data, please ensure that the statement adheres to our policy

All relevant data that support the plots within this paper and other findings of this study are available with the article and supplementary files. Other relevant data

during the study are available for research purposes from the corresponding authors upon reasonable request.

Research involving human participants, their data, or biological material

Policy information about studies with human participants or human data. See also policy information about sex, gender (identity/presentation), and sexual orientation and race, ethnicity and racism.

Reporting on sex and gender	n/a
Reporting on race, ethnicity, or other socially relevant groupings	n/a
Population characteristics	n/a
Recruitment	n/a
Ethics oversight	n/a

Note that full information on the approval of the study protocol must also be provided in the manuscript.

Field-specific reporting

Please select the one below that is the best fit for your research. If you are not sure, read the appropriate sections before making your selection.

- [x] Life sciences
- [ ] Behavioural & social sciences
- [ ] Ecological, evolutionary & environmental sciences

For a reference copy of the document with all sections, see nature.com/documents/nr-reporting-summary-flat.pdf

Life sciences study design

All studies must disclose on these points even when the disclosure is negative.

Sample size	No statistical method was used to predetermine the sample size for each study. For property measurement experiments, samples were prepared and tested at least twice. For in vivo studies, each group contains at least 3 (n ≥3) for evaluating the statistical significance. Sample size was chosen to ensure reproducibility of the experiments in accordance with the replacement, reduction and refinement principles of animal ethics regulation.
Data exclusions	No data was excluded from the analysis.
Replication	For property measurement experiments, samples were replicated and tested independently for 2-3 times and after analysis the standard deviation was displayed. For each experiment the statistical analysis is indicated in the figure legends.
Randomization	All samples were randomly allocated into experimental groups.
Blinding	No blinding was employed as the researcher performing the treatment was also responsible for the analysis. The researchers should keep careful track of protocols because that most of the experiments needed multiple treatments (including formulation, cells or mouse tumor treatment, sample collection, and so on). Hence, it would be difficult to blind the investigators to group allocation during data collection and analysis.

Reporting for specific materials, systems and methods

We require information from authors about some types of materials, experimental systems and methods used in many studies. Here, indicate whether each material, system or method listed is relevant to your study. If you are not sure if a list item applies to your research, read the appropriate section before selecting a response.

Materials & experimental systems

n/a	Involved in the study	
[ ]	[x]	Antibodies
[ ]	[x]	Eukaryotic cell lines
[x]	[ ]	Palaeontology and archaeology
[ ]	[x]	Animals and other organisms
[x]	[ ]	Clinical data
[x]	[ ]	Dual use research of concern
[x]	[ ]	Plants

Methods

n/a	Involved in the study	
[x]	[ ]	ChIP-seq
[ ]	[x]	Flow cytometry
[x]	[ ]	MRI-based neuroimaging

Antibodies

Antibodies used	1. Goat anti-HBsAg antibody (Bioss, catalog: #bs-1557G, 200 ×)<br>2. Cy3-conjugated Donkey Anti-Goat IgG H&L (Servicebio, catalog: #GB21404, 200 ×)
Validation	1. Goat anti-HBsAg antibody (catalog: #bs-15557G): http://www.bioss.com.cn/prolook_03.asp?id=AF08169606001427&pro37=1<br>2. Cy3-conjugated Donkey Anti-Goat IgG H&L (catalog: #GB21404): https://www.servicebio.cn/goodsdetail?id=256

Eukaryotic cell lines

Policy information about cell lines and Sex and Gender in Research

Cell line source(s)	The human cervical cancer cell line HeLa was purchased from the National Collection of Authenticated Cell Cultures (Shanghai, China).
Authentication	Cell lines have been authenticated by short tandem repeat profiling, and the results were compared with reference database.
Mycoplasma contamination	All cell lines tested negative for mycoplasma contamination.
Commonly misidentified lines<br>(See ICLAC register)	No commonly misidentified cell lines were used.

Animals and other research organisms

Policy information about studies involving animals; ARRIVE guidelines recommended for reporting animal research, and Sex and Gender in Research

Laboratory animals	Male C57/BL mice (3-5 weeks old) were obtained from the Laboratory Animal Center of Sun Yat-sen University (Guangzhou, China). Animals were housed under SPF conditions in groups of 4–5 mice per cage, maintained at a temperature of ~25 °C and a humidity of 30%-70% with a 12 h light/dark cycle, with free access to standard food and water.
Wild animals	This study did not involve wild animals.
Reporting on sex	This study did not involve reporting on sex.
Field-collected samples	This study did not involve sample collected from the field.
Ethics oversight	All animal experiments complied with Guidelines for Care and Use of Laboratory Animals of Sun Yat-sen University and approved by the Institutional Animal Care and Use Committee of Sun Yat-sen University (Guangzhou, China).

Note that full information on the approval of the study protocol must also be provided in the manuscript.

Flow Cytometry

Plots

Confirm that:

- [x] The axis labels state the marker and fluorochrome used (e.g. CD4-FITC).
- [x] The axis scales are clearly visible. Include numbers along axes only for bottom left plot of group (a 'group' is an analysis of identical markers).
- [x] All plots are contour plots with outliers or pseudocolor plots.
- [x] A numerical value for number of cells or percentage (with statistics) is provided.

Methodology

Sample preparation	The detached cells were washed three times with PBS to remove free nanoparticles. The cells were then resuspended in 200 ul PBS. In the flow cytometry analysis, at least 7000 cells were counted. The fluorescent data were represented as mean fluorescence intensity (MFI).
Instrument	BD FACS Calibur flow cytometer
Software	CytExpert
Cell population abundance	No cell sorting was performed.
Gating strategy	The preliminary FSC/SSC gates were determined by the blank cell samples.

- [x] Tick this box to confirm that a figure exemplifying the gating strategy is provided in the Supplementary Information.
