# Supplementary material for: An antifouling membrane-fusogenic liposome for effective intracellular delivery in vivo
Source: Nat Commun. 2024 May 20;15:4267. doi: 10.1038/s41467-024-46533-z (PMC11106281; doi:10.1038/s41467-024-46533-z)
Supplement: Supplementary file 3 — Peer Review File [file 41467_2024_46533_MOESM3_ESM.pdf]

nature portfolio

Peer Review File

An antifouling membrane-fusogenic liposome for effective intracellular delivery in vivo

Open Access This file is licensed under a Creative Commons Attribution 4.0 International License, which permits use, sharing, adaptation, distribution and reproduction in any medium or format, as long as you give appropriate credit to the original author(s) and the source, provide a link to the Creative Commons license, and indicate if changes were made. In the cases where the authors are anonymous, such as is the case for the reports of anonymous peer reviewers, author attribution should be to 'Anonymous Referee' followed by a clear attribution to the source work. The images or other third party material in this file are included in the article's Creative Commons license, unless indicated otherwise in a credit line to the material. If material is not included in the article's Creative Commons license and your intended use is not permitted by statutory regulation or exceeds the permitted use, you will need to obtain permission directly from the copyright holder. To view a copy of this license, visit http://creativecommons.org/licenses/by/4.0/.

Reviewers' Comments:

Reviewer #1 (Remarks to the Author)

This paper reports a nice systematic study of an antifouling membrane-fusogenic liposome (AFMFlip) as an effective delivery system for gene therapy, and it is benchmarked to two “traditional” fusogenic liposomes (MFlip-a and MFlip-b). The authors performed thorough in vitro and in vivo studies to demonstrate the superior capability of AFMFlip to resist protein adsorption, as protein adsorption is the key factor that hinders the membrane-fusion process of fusogenic liposomes. The authors conduct a range of comparisons, including protein adsorption, analysis of adsorbed protein composition, investigation of cellular internalization pathways using inhibitors and lysotracker, as well as in vivo experiments involving luciferase transfection and the assessment of HBV therapy effects. The study appears comprehensive. As a result of reduced protein adsorption, AFMFlip showed enhanced cellular uptake when being incubated in media containing FBS. The internalization pathway of AFMFlip does not switch from membrane-fusion to endocytosis until the concentration of FBS reaches 38%, much higher than that of MFlip-a and MFlip-b (4% and 6% respectively). Furthermore, AFMFlip showed improved transfection efficiencies in vitro and in vivo, compared to MFlip-a and MFlip-b. Finally, an effective AFMFlip-based gene therapy for HBV infection treatment is also reported. Regarding the novelty of the lipid formulation, it may not be entirely groundbreaking. However, the study itself is comprehensive and valuable.

Questions/Comments:

1. Does the antifouling effect really originate from the zwitterionic component? The authors claim that the antifouling effect is attributed to both reduced positive charge and the introduction of the zwitterionic DOPC lipid. However, the two traditional liposomes also contain zwitterionic lipids (DMPC and DOPE for MFlip-a and MFlip-b respectively). Thus, I do not think it is reasonable to conclude the zwitterionic property is the key contributor to the antifouling effect.

2. The introduction of zwitterionic lipids as helper lipids in conjunction with cationic DOTAP to allow fusion of liposomes with the cell membrane or endosomes is well established; this is not a new idea.

3. The control groups used for the systematic study lack control of variables. Using MFlip-a as an example, it uses DMPC instead of DOPC and contains more DOTAP and fewer DSPE-PEG than AFMFlip. Without controlling the variables of lipid composition, the conclusion that the contribution of DOPC is not persuasive. In addition, although the authors showed the reduced positive charge contributes to the antifouling effect (Figure 1b is more persuasive than comparing AFMFlip with MFlip-a), they ignored the influence of the DSPE-PEG ratio, which may also be a significant factor here.

4. Figure 2d: Clarification is needed regarding whether the DLS measurements on the instrument were conducted in water, FBS, or buffer. Additional information is required about the temperature at which the particles with FBS were treated, specifically whether it was at room temperature or 37°C.

5. Figures 2 and 3: It would be beneficial to also stain the cell membrane in the confocal images. Also, including a greater number of cells in the images would enhance the statistical significance of the findings and have better visualization. Figure 3a seems to lack noticeable differences except for MFlip-b with FBS. Additionally, consider performing experiments at different time intervals to investigate the influence of the lipid formulation on the cell membrane over time.

6. Figure 4: There is a missing subtitle for f.

7. Supplementary Figure 4: Error bars and significance analysis are absent.

8. Supplementary Figure 11: Some green overlap with the nuclei is observed. It would be valuable to clarify whether the nanoparticle delivery plasmid is present in the nuclei.

Reviewer #2 (Remarks to the Author)

Kong et al constructed antifouling membrane-fusogenic liposomes by utilizing high content (90%) zwitterionic lipids in the formulation of conventional membrane-fusogenic liposomes to decrease the surface charges and protein adsorption, retaining the membrane-fusion capacity of liposomes in the presence of high serum. These effects were verified via in vivo pDNA delivery and CRISPR/Cas9-mediated gene editing against hepatitis B virus infection in a mouse model. The findings are impressive and of interest to the community of biomedicine and nanotechnology. However, some critical concerns should be addressed before publication.

1. The relationship between protein resistance and membrane-fusion of AFMFlip should be carefully tested. 1) The author compared AFMFlip and the conventional MFlip and ascribed the maintenance of the membrane-fusion capacity in the presence of high serum proteins to the introduction of zwitterionic groups and the consequent decrease of surface charges of liposomes. However, the structure of hydrophobic tails of DOPC used in AFMFlip are difference from those in the conventional MFlip-a, too. Does this difference contribute to the membrane-fusion capacity besides the antifouling properties of liposomes? If the content of the zwitterionic DMPC in MFlip-a increases to 90%, does MFlip-a have membrane-fusion capability in the presence of high serum proteins? 2)Increasing the content of DSPE-PEG is able to decrease the surface charges and the protein adsorption. Does it work for the maintenance of membrane-fusion capacity of liposomes in the presence of high serum?

2. The adsorption of hepatocyte-target proteins may contribute to the accumulation of NPs in liver. There is big difference in protein adsorption for AFMFlip and MFlip-a or MFlip-b, why are all three liposomes accumulated in liver in Figure 6b and 6d? Is there any cell type-selectivity for the membrane-fusion of AFMFlip? How to avoid the fusion with the membrane of red blood cells during blood circulation?

3. The colocalization confocal imaging of plasma membrane and DiD is needed to confirm the fusion with plasma membrane in Figure 2b.

4. There is not enough information of NP-1…NP-5 in Figure 2b and these NPs were not discussed in the Results part. The author claimed that “cells treated with NP-2 and NP-3 in the FBS-free medium were observed with DiD (red) signals uniformly distributed on the cell membrane”. I don’t believe NP-3 clearly showed the uniform distribution around the nucleus.

5. Lipo2k forms NPs or nanocomplexes with plasmids, but not liposomes. It is not a good control to show the effect of FBS dependent fusion-endocytosis switch on the delivery efficacy of pDNA. Delivery with MFlip-a or MFlip-b should be performed for this purpose.

6. The statistical analysis should be performed for the groups of AFMFlip and MFlip-a or MFlip-b in Figure 5b.

7. There are mistakes in a, b, c…. numbering in the caption of Figure 4.

Reviewer #3 (Remarks to the Author)

The authors provide an interesting report on a fusogenic liposome formulation with lower protein-fouling than previous examples and improved fusion with cells at higher serum content and including two in vivo examples. However, before publication, there are several major and minor considerations to be taken into account.

Major comments:

- Can the authors please justify and put in literature context their choice of lipids for AFMFlip with respect to fusion (not the antifouling argument, which is understandable for DOPC/DSPE-PEG-containing liposomes)? Is DOPC/DOTAP/DSPE-PEG not a more standard liposome formulation, rather than a fusogenic one (incorporation of DOPE for fusion is standard but not DOPC)? For example, in papers by Agnes Csiszár et al. it was shown that DOTAP/DOPC shows very low fusion and adding DSPE-PEG into a fusogenic version (DOTAP/DOPE) did actually remove the fusogenic property:
1. https://www.ncbi.nlm.nih.gov/pmc/articles/PMC5855568/#sec2-ijms-19-00346
2. https://www.mdpi.com/2079-4991/9/7/1025

- Structural characterisation, e.g. cryoTEM, is missing, hence, the authors do not currently know whether their formulations (+/- plasmid) are liposomes or LNPs.

- Discussion on the relevance of protein corona needs to be changed and extended. The authors characterised nicely the protein corona forming on their three nanoparticle versions, including on AFMFlip. Hence, the more efficient liver accumulation of AFMFlip is not due to an absence of a corona (antifouling would suggest this) but more likely due to the specific proteins that accumulated on the AFMFlip surface. E.g. ApoE accumulation on LNPs has previously been shown to be responsible for hepatocyte uptake. The authors show here that ApoB is one of the main corona proteins (also on AFMFlip), what is the relevance of this? And how does their formulation compare to the well-known ionisable lipid-based nanoparticles (LNPs) previously used in some of the Covid vaccines, and for liver-targeted nucleic acid delivery, e.g. Onpattro. What is the advantage of their formulation over these others?

- Antifouling would suggest there is no/very little protein binding to the surface, I suggest calling it low fouling, as the authors still found quite a lot of protein binding also on AFMFlip (see Fig.2g). Throughout the text, statements like "AFMFlip could effectively retain membrane-fusion-dependent behavior in the presence of serum protein-containing medium, resulting from the ability to prevent protein corona formation" are not entirely accurate, as the authors have detected protein coronas on all three particle versions. Yes, AFMFlip binds less protein, but not non. Also, fusion is maintained up to 38% FBS, which is great, but physiologically, there is 100% FBS in serum; this should be discussed in more detail.

- For Fig.2b and 3a (also 5e), for the comparison of membrane fusion for various formulations, more than just a single/few cell/s per image is needed. Overview images with many cells should be provided in the SI at least. How did the authors obtain the dotted cell outline in Fig.2b? From these images, I cannot clearly see whether there is membrane fusion or not. Also, providing images for longer than 1h of incubation would clarify whether this was just membrane association, leading to dotted structures intracellularly a bit later, or whether this is actual membrane fusion. Using an additional membrane stain for identification (e.g. WGA) could help with conclusions too. For Fig.5e, the plasmid signal appears to be quite weak, do the authors have a negative control without plasmid to ensure this is not background autofluorescence?

- YOYO plasmid loading in liposomes did not involve any purification, how are the authors sure the signal in e.g. Fig.3a and corresponding flow cytometry data comes from released plasmid rather than uptake of free plasmid? Information on loading efficiency, i.e. percentage of free plasmid should be included.

Minor corrections:

- Abstract: Before reading the paper, it was not clear to me what % threshold is referring to: "Consequently, AFMFlip can perform a steady membrane-fusion-mediated gene delivery in the serum-containing medium with a 38% concentration threshold, outclassing two traditional MFlips (4% and 6% respectively)". Please reword.

- PDI for DLS is typically not given as a percentage, DLS x-axis log-scale normally.

- p14, DMPC or DOPC used in the formulations? Both are mentioned currently.

- Number of experiments/repeats (N=XX, n=xx) need to be provided everywhere, e.g. for Fig. 2h, without showing dots in graph, not clear how many repeats were done here?

- Fig.3a; the cross-sections, especially for the bottom examples, could easily have been drawn differently in these figures, which would change the conclusion (there is clearly green within the cells for MFLips+10%FBS, the lines have just not been drawn through that area)

- Fig.4; errors in caption regarding letter labelling

- Fig.5d; quantitative information on TEM image analysis of a single particle per sample should be removed from the image (red lines) and in the text. I cannot see how the authors choose to put these lines and if doing quantification, it should be done on at least several dozens of particles per sample, not just on one.

Response to Referees

Response to Reviewer 1

This paper reports a nice systematic study of an antifouling membrane-fusogenic liposome (AFMFlip) as an effective delivery system for gene therapy, and it is benchmarked to two "traditional" fusogenic liposomes (MFlip-a and MFlip-b). The authors performed thorough *in vitro* and *in vivo* studies to demonstrate the superior capability of AFMFlip to resist protein adsorption, as protein adsorption is the key factor that hinders the membrane-fusion process of fusogenic liposomes. The authors conduct a range of comparisons, including protein adsorption, analysis of adsorbed protein composition, investigation of cellular internalization pathways using inhibitors and lysotracker, as well as *in vivo* experiments involving luciferase transfection and the assessment of HBV therapy effects. The study appears comprehensive. As a result of reduced protein adsorption, AFMFlip showed enhanced cellular uptake when being incubated in media containing FBS. The internalization pathway of AFMFlip does not switch from membrane-fusion to endocytosis until the concentration of FBS reaches 38%, much higher than that of MFlip-a and MFlip-b (4% and 6% respectively). Furthermore, AFMFlip showed improved transfection efficiencies *in vitro* and *in vivo*, compared to MFlip-a and MFlip-b. Finally, an effective AFMFlip-based gene therapy for HBV infection treatment is also reported. Regarding the novelty of the lipid formulation, it may not be entirely groundbreaking. However, the study itself is comprehensive and valuable.

Response: Thank you very much for your positive evaluation and comments.

1. Does the antifouling effect really originate from the zwitterionic component? The authors claim that the antifouling effect is attributed to both reduced positive charge and the introduction of the zwitterionic DOPC lipid. However, the two traditional liposomes also contain zwitterionic lipids (DMPC and DOPE for MFlip-a and MFlip-b respectively). Thus, I do not think it is reasonable to conclude the zwitterionic property is the key contributor to the antifouling effect.

Response: Thank you very much for your helpful comment. The antifouling effect of our developed AFMFlip, composed of DOPC/DOTAP/DSPE-PEG (90/5/5), is primarily due to the use of the zwitterionic DOPC lipid, known for its anti-protein adsorption capabilities, as extensively documented in prior studies[1-4]. To further validate the DOPC-mediated antifouling effect of AFMFlip, we also prepared a contrastive liposome (DOPA-lip) devoid of zwitterionic groups on the surface, substituting DOPC with 1,2-dioleoyl-*sn*-glycero-3-phosphate (DOPA). After the exposure to 10% FBS, there was a notable increase in the particle size of DOPA-lip, from 184.6 nm to 216.4 nm, and a shift in the zeta potential from -16.2 mV to -23.9 mV (**Fig. R1b-c**). In contrast, AFMFlip demonstrated minimal changes in both particle size and zeta potential following FBS treatment. More importantly, a BCA-based protein quantification analysis revealed a significantly higher protein accumulation on the surface of DOPA-lip compared to AFMFlip (**Fig. R1d**). These findings collectively indicate that the antifouling effect of AFMFlip is facilitated by the zwitterionic groups derived from the DOPC lipid.

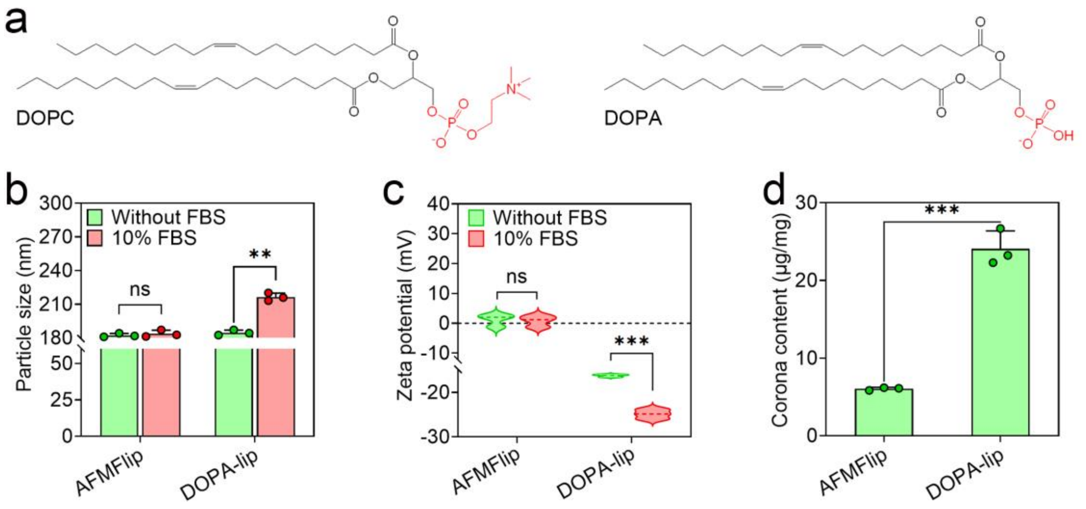

Fig. R1 | Characteration and antifouling effect of AFMFlip and DOPA-lip. a Structural difference between DOPC and DOPA used in AFMFlip and DOPA-lip, respectively. **b, c** Hydrodynamic sizes (**b**) and zeta potentials (**c**) of AFMFlip and DOPA-lip after incubation without or with 10% FBS (n = 3). **d** Protein corona quantification with bicinchoninic acid (BCA) assay in the presence of 10% FBS (n = 3). ns: $p > 0.05$, **$p < 0.01$, ***$p < 0.001$ (two-tailed t-

test).

The traditional MFlips, MFlip-a, and MFlip-b, are also constructed using zwitterionic lipids (DMPC and DOPE, respectively). However, MFlip-a consists of a relatively lower proportion of zwitterionic lipid (DMPC, 76.2%) and a higher level of cationic DOTAP (20%) compared to our AFMFlip (DOPC, 90%; DOTAP, 5%). This composition results in MFlip-a having a positively charged surface, which tends to facilitate protein adsorption through electrostatic interactions [5-8], thereby diminishing its antifouling efficacy. Similarly, MFlip-b is formulated with a lower percentage of zwitterionic DOPE lipid (69.5%), significantly less than AFMFlip. Moreover, the phosphoethanolamine group present in the DOPE is known to have a weaker antifouling capacity compared to phosphorylcholine in the DOPC[9]. Collectively, these factors contribute to our AFMFlip demonstrating a more potent antifouling effect than the traditional MFlips, attributable to its higher content of zwitterionic groups and a reduced positive charge on its surface.

2. The introduction of zwitterionic lipids as helper lipids in conjunction with cationic DOTAP to allow fusion of liposomes with the cell membrane or endosomes is well established; this is not a new idea.

Response: Thank you very much for your comment. Indeed, most of the currently-used MFlips are formulated using a mixing component of neutral lipid (typically zwitterionic lipids like DOPE and DMPC) and cationic lipid (commonly DOTAP), sometimes incorporating PEGylated lipid[7,8,10-13]. The neutral lipid, which usually constitutes over 50% of the mixture, should have a moderately low phase transition point, close to room temperature. This composition ensures that the fluidity of the lipid layer remains similar to that of the cell membrane. The cationic lipid, on the other hand, facilitates cellular uptake and fusion through electrostatic attraction with the cell membranes. Meanwhile, the presence of PEGylated lipid aids in dehydrating the space between the fusogenic lipid coating and the plasma membrane, making lipid fusion both energetically and structurally more favorable.

However, traditional MFlips, created using the conventional approach, typically contain a high percentage of cationic lipids (at least 20%). This composition results in a positively charged surface, which readily interacts electrostatically with serum proteins, leading to the formation of a thick protein corona[5-8]. Such protein adsorption poses a significant challenge; it impedes the ability of the fusogenic lipids on the surface to establish close contact with cell

membranes, thereby interfering with the membrane fusion process[5]. This reduction in membrane fusion efficiency in a protein-enriched environment severely compromises their intracellular delivery efficiency *in vivo*, greatly limiting the clinical application of their related medical products[5].

In our study, we developed a lipid coating consisting of zwitterionic DOPC, cationic DOTAP, and DSPE-PEG in a specific ratio of 90/5/5. This composition enabled a stable membrane-fusion interaction with cells, even in a medium containing 38% FBS. Unlike traditional MFlips, our AFMFlip has a higher proportion of zwitterionic lipid DOPC (90%) and a reduced amount of cationic lipid DOTAP (5%). This ratio significantly enhances resistance to protein adsorption, facilitating consistent membrane-fusion interactions with cell membranes. Despite being composed of commonly used lipids, our AFMFlip demonstrates exceptional efficacy in membrane-fusion-mediated delivery in protein-rich environments, both *in vitro* and *in vivo*. While the design of AFMFlip draws upon previous MFlip construction strategies, its extraordinary membrane-fusion performance in the body's complex environments marks a significant advancement for practical in vivo applications. These promising features underscore its importance for the future development of liposome-based nanomedicine.

We have rephased the descriptions related to the "novel strategy" of introducing zwitterionic lipids into MFlips in the manuscript, and highlighted the advantages and significance of our study (marked in red) more clearly, which was revised as follows:

"Based on this protein-resistance capacity, we optimistically assume that introducing an abundant ratio of zwitterionic groups onto the surfaces of MFlips can preserve their membrane-fusion efficiency in the protein-enriched biological environment by forming a non-fouling surface with reduced protein adsorption. While some MFlips have utilized zwitterionic lipids like DMPC and DOPE, their membrane-fusion performance in the protein environment has often been subpar, compromised by the concurrently-used cationic lipids."

"Overall, this AFMFlip presents a pioneering type of MFlips that remarkably maintains membrane-fusion effectiveness even in serum protein-enriched environments, offering an efficient nanocarrier for *in vivo* intracellular delivery of therapeutics."

"The protein corona formed on MFlip surface during body circulation is the main cause of a failure of membrane-fusion-based transportation *in vivo*. The integration of antifouling zwitterionic materials on the MFlip surface can prevent protein corona formation, thereby sustaining the membrane-fusion capability under protein-enriched physiological conditions."

3. The control groups used for the systematic study lack control of variables. Using MFlip-a as an example, it uses DMPC instead of DOPC and contains more DOTAP and fewer DSPE-PEG than AFMFlip. Without controlling the variables of lipid composition, the conclusion that the contribution of DOPC is not persuasive. In addition, although the authors showed the reduced positive charge contributes to the antifouling effect (Figure 1b is more persuasive than comparing AFMFlip with MFlip-a), they ignored the influence of the DSPE-PEG ratio, which may also be a significant factor here.

Response: Thank you very much for your insightful comment. Before evaluating the antifouling effect, it is crucial that the liposomes are designed with the capability to fuse with cell membranes. This membrane-fusion performance hinges on both the structures and ratios of lipid components[8,10,14-16]. According to the previous studies, MFlips can be constructed using three lipid components: (1) a substantial portion of neutral lipid to modulate fluidity and facilitate the fusion; (2) cationic DOTAP for electrostatic attraction to the cell membrane; (3) DSPE-PEG to dehydrate the space between the nanoparticle lipid coatings and the plasma membrane[10,11]. After selecting appropriate lipid components, their composition ratio should be subsequently screened for optimal membrane fusion[8,14]. In our study, we selected DOPC, a widely-used zwitterionic lipid known for its antifouling properties[1-4], along with cationic DOTAP and DSPE-PEG for liposome construction and optimized their optimal ratio as 90/5/5 for membrane fusion. MFlip-a (DMPC/DOTAP/DSPE-PEG, 76.2/20/3.8) and MFlip-b (DOPE/CHEMS/DSPE-PEG, 69.5/18.2/12.3) are also previously reported MFlips, each with its optimal lipid composition ratio. Therefore, the lipid compositions of these liposomes in our study are very different and cannot be standardized with a single variable. To demonstrate the contribution of DOPC, we prepared a comparative liposome (DOPA-lip) using DOPA, DOTAP, and DSPE-PEG in a 90/5/5 ratio. DOPA-lip has a similar lipid structure, composite, and size with AFMFlip, but lacks zwitterionic groups on its surface (**Fig. R1a**). However, DOPA-lip showed increased protein adsorption on the surface and more pronounced changes in size and zeta potential after incubation with 10% FBS (**Fig. R1b-d**). This single-variable comparison sufficiently indicated the antifouling effect mediated by the zwitterion groups on the DOPC.

A small amount of DSPE-PEG is crucial in dehydrating the space between the nanoparticle lipid coatings and the plasma membrane, as well as in maintaining the stability of the liposomes. To assess the impact of the PEG component, we prepared a series of liposomes with a constant DOTAP content (5%) and varying molar ratios of DSPE-PEG and DOPC (**Fig. R2a**). We then evaluated their resistance to protein adsorption after exposure to 10% FBS. The results, depicted in **Fig. R2b** and **c**, indicate that increasing DSPE-PEG to 10% began to alter the liposomes'

particle sizes and zeta potentials following FBS exposure, highlighting DOPC's superior anti-protein-adsorption capacity compared to DSPE-PEG. To confirm this observation, we quantified the proteins adsorbed on the liposomes using a BCA-based assay after incubation in 10% FBS (**Fig. R2d**) and mouse serum (**Fig. R2e**). Consistently, liposomes with more than 10% DSPE-PEG showed significant protein adsorption on their surfaces, while those containing 0-5% DSPE-PEG demonstrated relatively low protein adsorption. These results confirmed the antifouling superiority of the zwitterionic DOPC component in the protein-containing condition compared with the DSPE-PEG, which was consistent with previous studies[17,18].

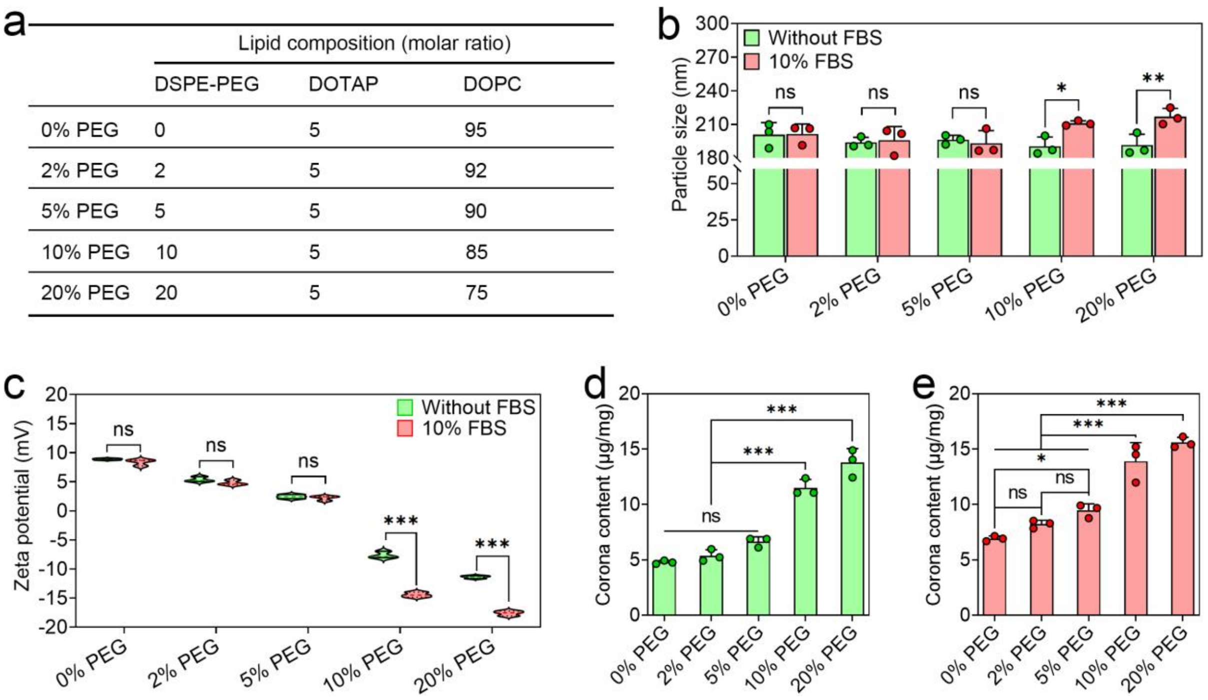

Fig. R2 | Influence of DSPE-PEG on the antifouling effect of AFMFlip. a The lipid construction of liposomes with different DSPE-PEG ratios. **b, c** Hydrodynamic sizes (**b**) and zeta potentials (**c**) of liposomes after incubation in the medium without or with 10% FBS (n = 3). **d, e** Protein corona quantification with BCA assay in the presence of 10% FBS (**d**) and mouse serum (**e**) (n = 3). ns: $p > 0.05$, $^{**}p < 0.01$, $^{***}p < 0.001$ (two-tailed t-test).

We further assessed the impact of PEG on the membrane-fusion capacity of AFMFlip using Confocal Laser Scanning Microscopy (CLSM) observation and a Fluorescence Resonance Energy Transfer (FRET)-based assay[19,20]. CLSM images revealed that only cells treated with liposomes comprising 5% DSPE-PEG, 90% DOPC, and 5% DOTAP exhibited DiD (red) signals on the cell membrane (**Fig. R3a**), indicating the membrane fusion-mediated uptake. For the FRET-based assay, we labeled the lipid coating of these liposomes with both DiO and DiI. In this configuration, the fluorescence pair underwent a FRET effect (excited at Ex (DiO) = 483

nm, with emission at Em (DiI) = 565 nm). We set this spectrum as a negative control representing a 0% fusion point, and the spectrum of this suspension post Triton X-100 addition at 0.1% (v/v) as indicative of a 100% fusion pattern.

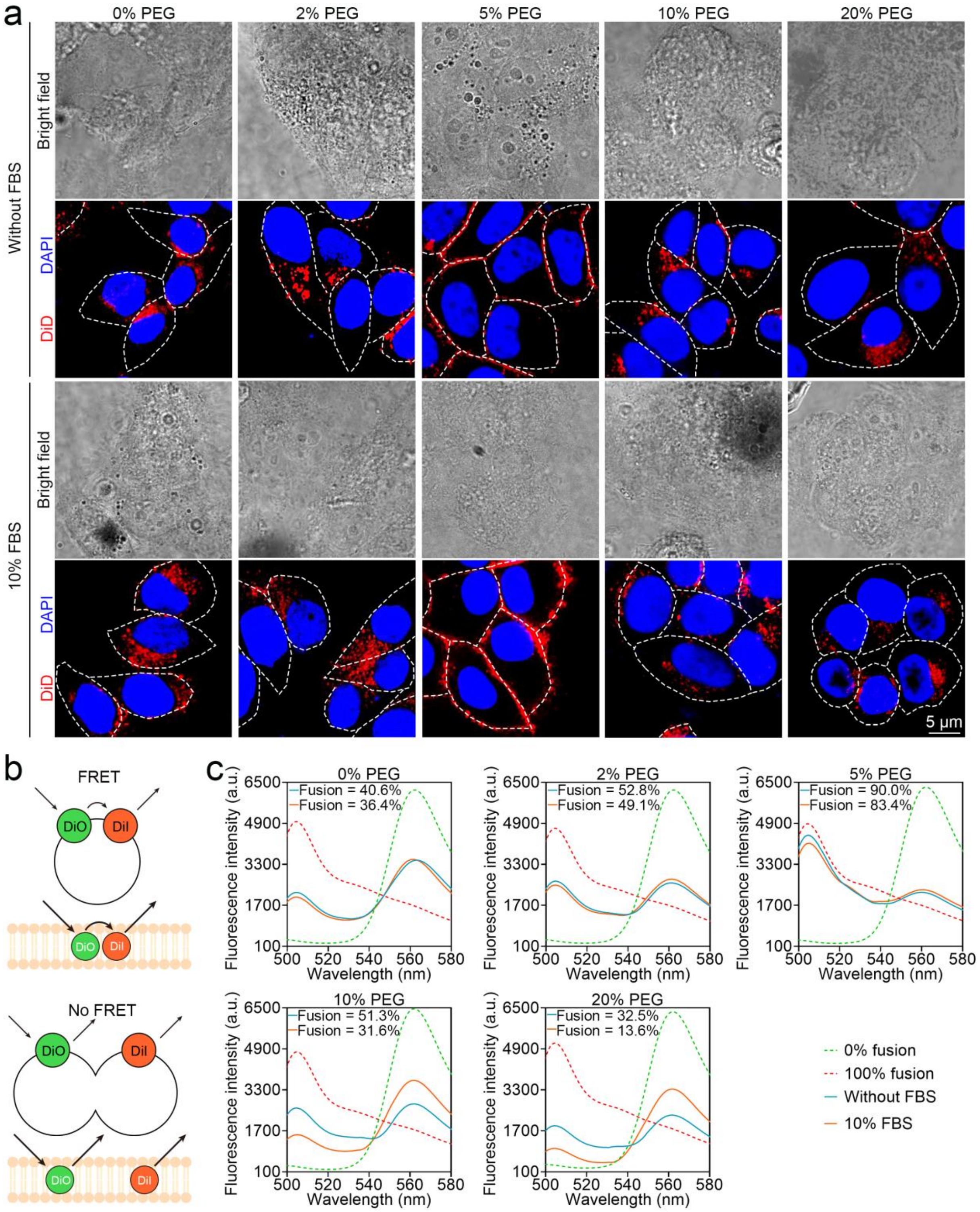

Fig. R3 | Evaluation of the impact of DSPE-PEG on the membrane-fusion efficacy of AFMFlip. a Fluorescence microscopy images of HeLa cells treated with different DiD-labeled liposomes without or with 10% FBS for 1 h. The white dotted lines indicate the cellular outlines

according to the images of the bright field. **b** Schematic illustration of fluorescence resonance energy transfer (FRET)-based assay to evaluate the liposome-cell fusion. **c** The emission fluorescence spectra of different constructed liposomes labeled with DiO and DiI incubation with HeLa cells for 1 h under the condition without or with FBS at 37 °C. The initial fluorescence of the labeled liposome suspension was recorded as 0% fluorescence (0% fusion, green lines), and the 100% fluorescence (100% fusion, red lines) was determined after adding Triton X-100 at 0.1% (v/v).

After incubation with cells in the presence/absence of 10% FBS for 1 h at 37 °C, the fluorescence spectra of liposome/cell mixtures were also recorded to determine the membrane-fusion performance by the formula: Fusion (%) = (F_{DiO} - F0) / (F100 - F0). As shown in **Fig R3b**, the liposomes with a 5% DSPE-PEG ratio demonstrated optimal membrane-fusion efficiency with cells, even in conditions containing FBS. This result was in accordance with the previous studies, which showed that PEG assisted in dehydrating the space between the fusogenic lipid coating on the nanoparticle and the plasma membrane, thereby making lipid fusion both energetically and structurally favorable[11]. Overall, the specific ratio of DOPC, DOTAP, and DSPE-PEG in our AFMFlip plays a crucial role in both its antifouling effect and membrane-fusion performance.

4. Figure 2d: Clarification is needed regarding whether the DLS measurements on the instrument were conducted in water, FBS, or buffer. Additional information is required about the temperature at which the particles with FBS were treated, specifically whether it was at room temperature or 37 °C.

Response: Thank you very much for your thorough review and helpful reminder. We have incorporated detailed information about our experiments in the description of **Fig. 2** and its legends, listed as follows:

"Primarily, the physical characterizations of these MFlips were determined after 1-h incubation without or with 10% FBS at 37 °C. The exposure to FBS caused negligible changes in particle size and zeta potential of AFMFlip (**Fig. 2d, e**);"

"Hydrodynamic sizes (**d**) and zeta potentials (**e**) of AFMFlip, MFlip-a, and MFlip-b after 1-h incubation in the medium without or with 10% FBS at 37 °C (n = 3). ns: $p > 0.05$, *$p < 0.05$, **$p < 0.01$, ***$p < 0.001$ (two-tailed t-test). Measurements were conducted in water at room temperature."

Moreover, we have revised the related part of Methods in our manuscript as follows: "After that, the collected precipitates were washed carefully with sterile deionized water, followed by measurements of particle sizes and zeta potentials at room temperature."

5. Figures 2 and 3: It would be beneficial to also stain the cell membrane in the confocal images. Also, including a greater number of cells in the images would enhance the statistical significance of the findings and have better visualization. Figure 3a seems to lack noticeable differences except for MFlip-b with FBS. Additionally, consider performing experiments at different time intervals to investigate the influence of the lipid formulation on the cell membrane over time.

Response: Thank you very much for your valuable advice. For **Fig. 2b**, we have repeated the experiment, followed by staining the cell membrane and capturing more cells as you suggested for improved visualization. As evident in the updated CLSM images in **Fig. R4a** (**Fig. 2b** and **Supplementary Fig. 1a** in the revised version), cells treated with NP-2, exhibited uniformly-distributed DiD (red) signals on the labeled membrane (green), suggesting the potential uptake through membrane fusion. More importantly, when exposed to the 10% FBS-containing medium, only cells treated with NP-2 showed persistent red-green fluorescence overlap patterns. In contrast, cells treated with other liposomes exhibited either dotted or spread green fluorescence signals throughout. To further corroborate this observation, we employed a FRET-based assay to quantify the membrane fusion efficiency, thereby providing robust statistical evidence. Results in **Fig. R4b** demonstrated that NP-2 achieved the highest level of fusion in the 10% FBS-containing and FBS-free media (86.0% and 86.2%, respectively). This indicates that NP-2, with its molar ratio of 90/5/5 (DOPC/DOTAP/DSPE-PEG), is capable of maintaining fusion behavior in protein-rich conditions. These data have been added as the updated **Supplementary Fig. 1b** in the updated version. Additionally, corresponding updates and revisions have been made to the text and Methods section, marked in red for clarity.

For **Fig. 3a**, we have also repeated the experiments and stained the cell membrane for better visualization. As shown in **Fig. R5c** (updated as **Fig. 3a** and **Supplementary Fig. 12a** in the revised version), these dual-labeled MFlips performed an obvious membrane-fusion interaction with HeLa cells in the FBS-free medium. This is evident as the fluorescent signals from the DiD-labeled shell (red) were predominantly located on the CellMask-labeled plasma membrane (cyan), while YOYO-1-labeled plasmids (green) were spread into the cytoplasm in the CLSM images.

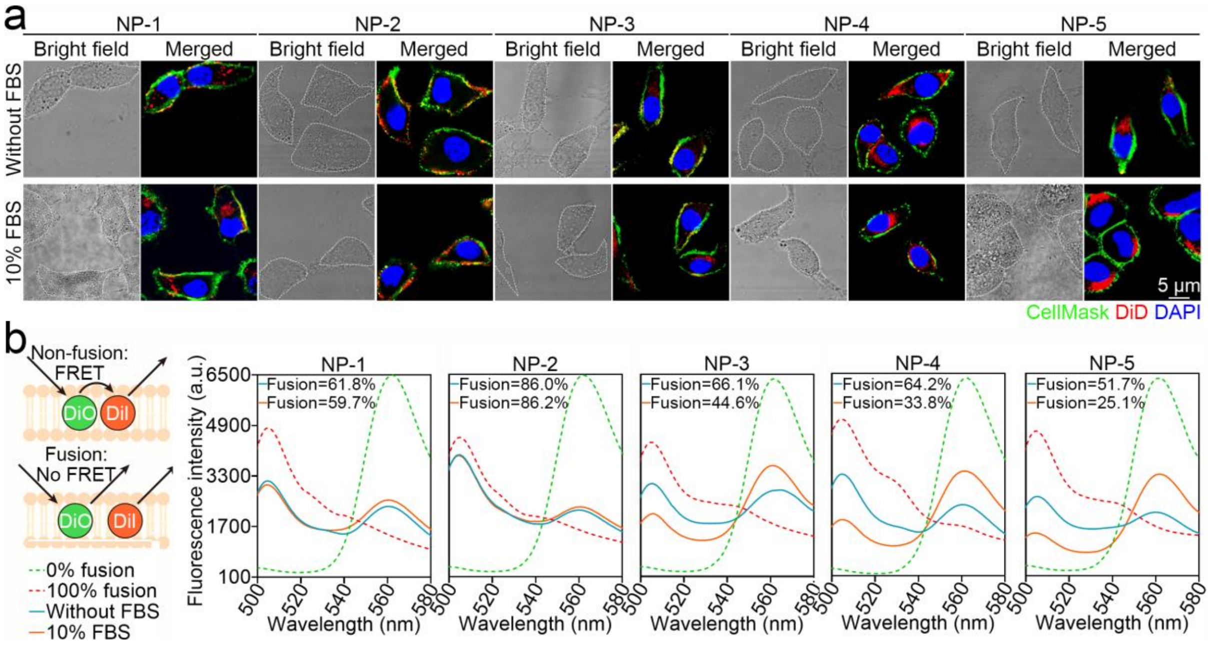

Fig. R4 | Optimization of AFMFlip with serum-resistance membrane-fusion capacity. a CLSM images of liposomes (NP-1 to NP-5 with different lipid component ratios) in the medium with or without 10% FBS. The liposomes were labelled with 1,1'-dioctadecyl-3,3,3',3'-tetramethylindodicarbocyanine,4-chlorobenzenesulfonate salt (DiD). The nuclei were stained with DAPI and the cell membranes were labelled with CellMask. **b** FRET analysis to evaluate the membrane-fusion efficacy after 1 h incubation with cells in the medium with or without 10% FBS at 37 °C.

However, in a medium containing 10% FBS, only DiD-AFMFlip$^{YOYO\text{-}1}$ continued to interact with the cells *via* a membrane-fusion mechanism. In contrast, DiD-MFlip-a$^{YOYO\text{-}1}$ and DiD-MFlip-b$^{YOYO\text{-}1}$ exhibited some overlap of red and green signals in the cytoplasm. Additional fluorescence intensity line scanning analysis, presented in **Fig. R5d** (updated as **Fig. 3a** in the revised manuscript), also confirmed these results, indicating the maintained fusion effect of AFMFlip and the involvement of endocytosis in the uptake of MFlip-a and MFlip-b in FBS-containing medium. We have added these results in our revised manuscript. Moreover, we have updated the related descriptions and marked them in red in both the revised manuscript and Supplementary Information according to the above illustration.

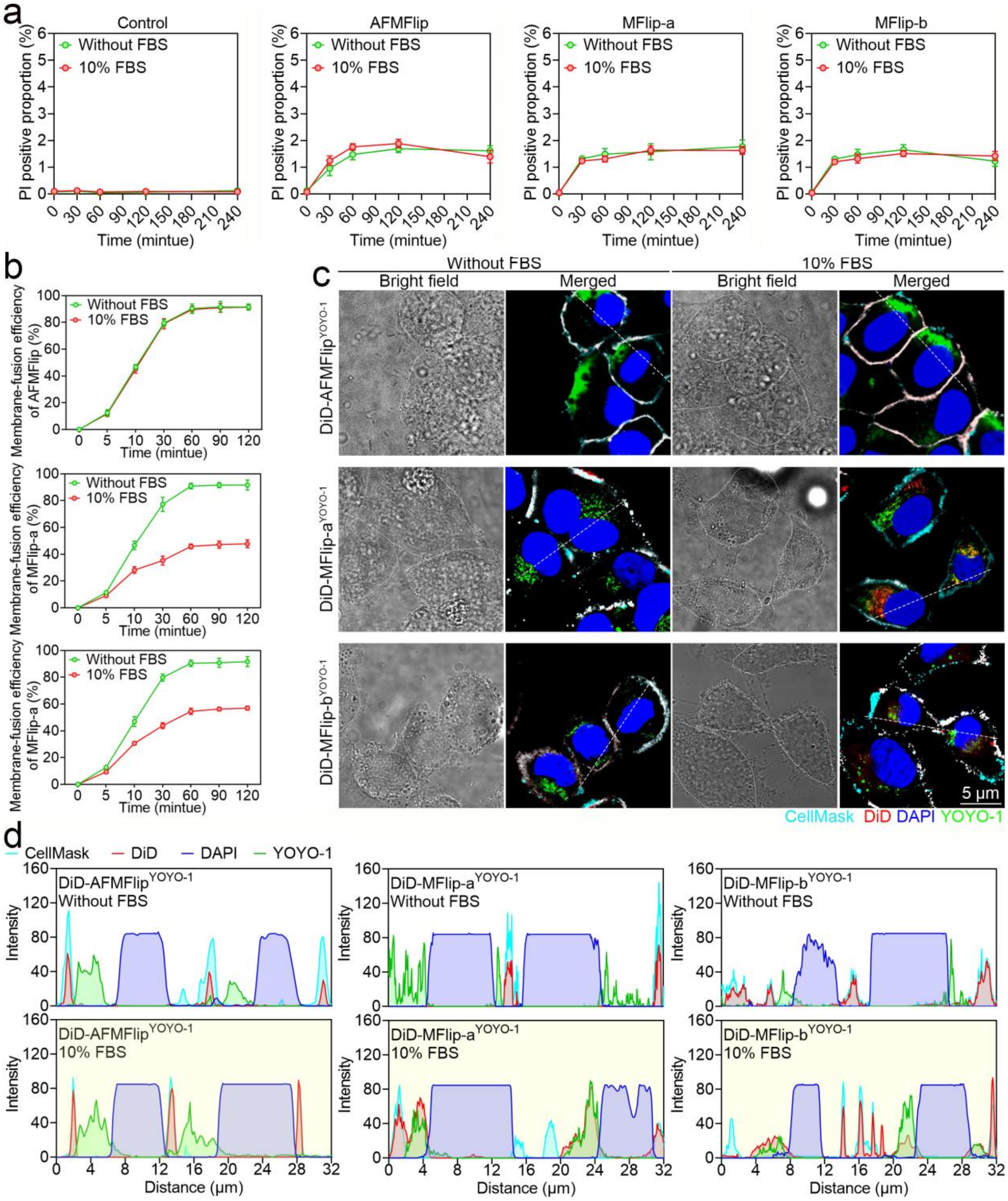

Fig. R5 | Membrane-fusion performance of AFMFlip, MFlip-a, and MFlip-b in the medium with or without 10% FBS. a Quantitative analysis of propidium iodide (PI)-positive cells post-treatment with AFMFlip, MFlip-a, and MFlip-b in media without or with 10% FBS at 37 °C. **b** Time-dependent membrane-fusion performance of AFMFlip, MFlip-a, and MFlip-b based on FRET analysis after cell incubation without or with 10% FBS at 37 °C. **c** CLSM images of the cellular uptake of DiD-AFMFlip^{YOYO-1}, DiD-MFlip-a $^{YOYO-1}$, and DiD-MFlip-b $^{YOYO-1}$ in the medium with or without 10% FBS. The liposomes were labelled with DiD. The nuclei were stained with DAPI, and the cell membranes were labelled with CellMask. **d** Line

scanning analysis along the white lines by ImageJ software.

Additionally, we investigated the influence of the lipid formulation on the cell membrane over time *via* propidium iodide (PI) staining. **Fig. R5a** (**Supplementary Fig. 12c** in the updated Supplementary Information) showed that all MFlips induced less than 2% damaged cells over time, indicating the preservation of membrane integrity after MFlip treatment. Moreover, we quantified the membrane-fusion efficiency of AFMFlip, MFlip-a, and MFlip-b at different time intervals using FRET analysis. As shown in **Fig. R5b** (**Supplementary Fig. 12b** in the updated Supplementary Information), AFMFlip, MFlip-a, and MFlip-b exhibited a nearly 90% fusion performance after 1 h incubation with cells in the FBS-free medium. However, upon exposure to 10% FBS, only AFMFlip continued to demonstrate high fusion efficiency with cells after 1 h incubation, whereas the efficiency for both MFlip-a and MFlip-b decreased markedly. Notably, these time-dependent curves indicated that the membrane-fusion efficiency of these liposomes reached the plateau at 1 h, which informed our decision to choose a 1-hour incubation period for the CLSM observation experiments.

6. Figure 4: There is a missing subtitle for f.

Response: Thank you very much for your meticulous review and helpful reminder. We have addressed the error and included a subtitle for Figure 4f. The detailed revision is as follows: "…..**f** EGFP expression in HeLa cells observed by fluorescence microscopy transfected with AFMFlip^{pEGFP} at different FBS concentrations."

7. Supplementary Figure 4: Error bars and significance analysis are absent.

Response: Thank you very much for your thoughtful comment. The raw abundance of proteins was derived from a semi-quantitative assessment and identification of protein corona on the nanoparticle surface, based on LC-MS/MS analysis, from a single sample (**Fig. R6**)[5,21]. Consequently, the inclusion of error bars and significance analysis is not applicable for this figure. It's noteworthy that the semi-quantitative results displayed in Supplementary **Fig. 4** closely align with the protein amounts quantified using the BCA method (Supplementary **Fig. 3**).

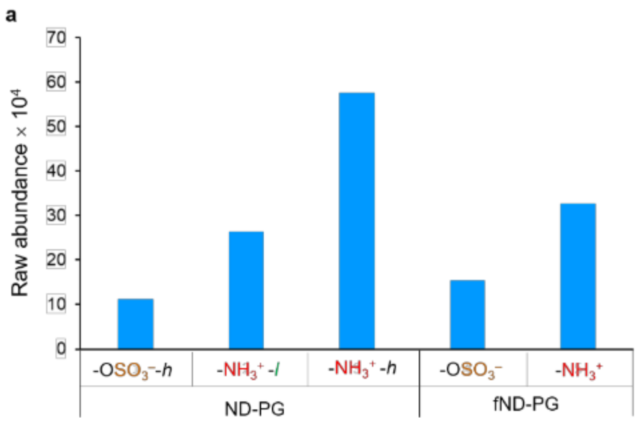

Fig. R6 | Total relative raw abundances of proteins on the nanoparticle surface. Reproduced from[21]. Copyright 2022, Wiley-VCH.

8. Supplementary Figure 11: Some green overlap with the nuclei is observed. It would be valuable to clarify whether the nanoparticle delivery plasmid is present in the nuclei.

Response: Thank you very much for your thorough review and constructive comment. In our design, we utilized a plasmid/$CaCO_3$ core to construct core-shell MFlips. Supporting our approach, previous studies have indicated that calcium ions can facilitate the transfer of DNA into the cell nucleus[22,23]. Consequently, the presence of calcium in MFlips may also enhance the nuclear entry of plasmid cargos. Moreover, as shown in the figure you referenced, only the plasmid from AFMFlip in the medium containing 10% FBS exhibited a relatively higher overlap with the nuclei. This indicates that AFMFlip is capable of executing membrane-fusion-mediated intracellular delivery, enabling direct cytoplasmic cargo release without endocytosis/lysosomal entrapment. Such a mechanism could significantly increase the likelihood of successful nuclear entry of the delivered cargo.

Response to Reviewer 2

Kong *et al*. constructed antifouling membrane-fusogenic liposomes by utilizing high content (90%) zwitterionic lipids in the formulation of conventional membrane-fusogenic liposomes to decrease the surface charges and protein adsorption, retaining the membrane-fusion capacity of liposomes in the presence of high serum. These effects were verified *via in vivo* pDNA delivery and CRISPR/Cas9-mediated gene editing against hepatitis B virus infection in a mouse model. The findings are impressive and of interest to the community of biomedicine and nanotechnology. However, some critical concerns should be addressed before publication.

Response: We sincerely appreciate your positive evaluation and insightful comments.

1. The relationship between protein resistance and membrane-fusion of AFMFlip should be carefully tested. 1) The author compared AFMFlip and the conventional MFlip and ascribed the maintenance of the membrane-fusion capacity in the presence of high serum proteins to the introduction of zwitterionic groups and the consequent decrease of surface charges of liposomes. However, the structure of hydrophobic tails of DOPC used in AFMFlip are difference from those in the conventional MFlip-a, too. Does this difference contribute to the membrane-fusion capacity besides the antifouling properties of liposomes? If the content of the zwitterionic DMPC in MFlip-a increases to 90%, does MFlip-a have membrane-fusion capability in the presence of high serum proteins? 2) Increasing the content of DSPE-PEG is able to decrease the surface charges and the protein adsorption. Does it work for the maintenance of membrane-fusion capacity of liposomes in the presence of high serum?

Response: Your professional comments and valuable suggestions are greatly appreciated. We presented **Fig.** R7 to demonstrate the relationship between protein corona content and membrane-fusion performance of AFMFlip. The result showed that the fusion effect would be affected when the protein corona on the surface of AFMFlip approached approximately 13 μg/mg.

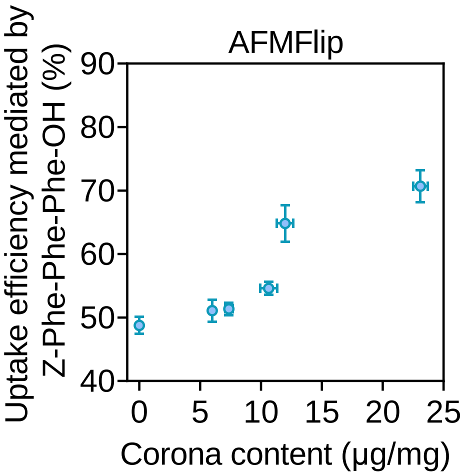

Fig. R7 | The correlation between protein corona content absorbed on the surface and uptake efficiency mediated by Z-Phe-Phe-Phe-OH (membrane-fusion inhibitor) (n=3).

Regarding your specific inquiries, our responses are as follows.

1) To investigate the influence of the hydrophobic tails of DOPC on the antifouling effect of AFMFlip, we formulated a liposome (DMPC-lip) using DMPC, DOTAP, and DSPE-PEG in the same ratio of 90/5/5. As shown in **Fig. R8a-c**, DMPC-lip also exhibited a consistent anti-protein-adsorption ability with AFMFlip, as demonstrated by the alike size, zeta potential, and protein corona content in the medium with or without 10% FBS. This suggested that the structural differences in the hydrophobic tails of DOPC and DMPC did not significantly affect the antifouling effect of AFMFlip. Moreover, we evaluated the membrane-fusion efficiency of DMPC-lip using FRET analysis[19,20].

The results in **Fig. R8d** showed a considerably lower membrane-fusion efficiency for DMPC-lip compared to AFMFlip, both with and without 10% FBS. This indicated that DMPC-lip was less effective in achieving membrane fusion with cells. Collectively, we concluded that AFMFlip with a specific ratio of DOPC/DOTAP/DSPE-PEG (90/5/5) can not only facilitate a membrane-fusion process but also maintain this effect in the protein-rich media owing to the abundant zwitterionic components of DOPC on the surface.

Despite utilizing DMPC, MFlip-a achieves membrane fusion only at a specific ratio of 76.2/20/3.8 (DMPC, DOTAP, and DSPE-PEG)[11], which differs from the composition of AFMFlip (90/5/5, DOPC/DOTAP/DSPE-PEG). The distinct characteristics of DOPC, such as its unsaturated lipid tails and longer alkyl chain, account for this variation in the optimal lipid ratio for fusion. These features modulate the fluidity of the lipid coating and enhance its interaction with the plasma membrane[24-27]. This increased fluidity afforded by DOPC allows AFMFlip to require less DOTAP for effective interaction with the cell membrane, thereby

initiating the fusion process while preserving a higher proportion of zwitterionic groups on its surface. Consequently, our AFMFlip design, with a reduced amount of cationic DOTAP, is still capable of inducing membrane fusion. This is particularly effective in environments with high serum content, where the antifouling properties of AFMFlip are most beneficial.

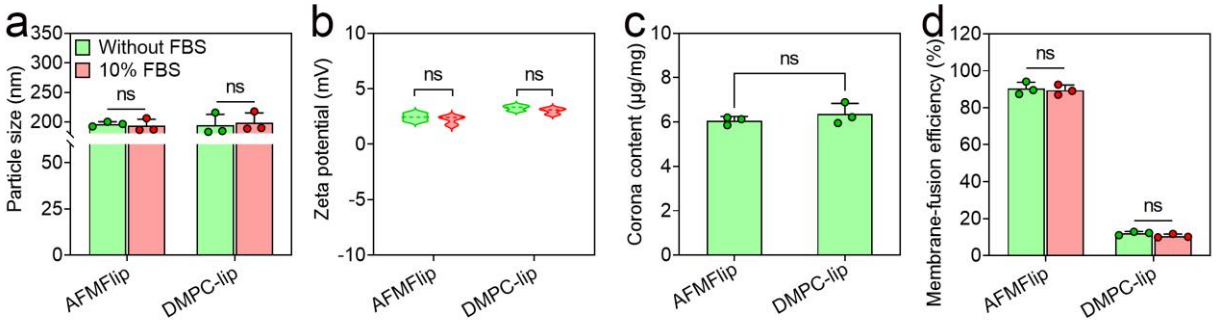

Fig. R8 | Comparison of Physical characteristics, membrane-fusion capacity, and protein absorption of DMPC-lip relative to AFMFlip. a, b Hydrodynamic sizes (**a**) and zeta potentials (**b**) of AFMFlip and DMPC-lip after 1 h incubation in the medium without or with 10% FBS at 37 °C (n = 3). **c** Protein corona quantification with BCA assay in the presence of 10% FBS (n = 3). ns: $p > 0.05$, (two-tailed t-test). **d** Evaluation of membrane-fusion efficiency through FRET analysis for AFMFlip and DMPC-lip post 1-hour cell incubation in media, both with and without 10% FBS at 37 °C (n = 3).

2) A minor proportion of DSPE-PEG in liposomes plays a crucial role in dehydrating the space between the nanoparticle lipid coatings and the plasma membrane, while also contributing to the stability of the liposomes. To examine the influence of the PEG component, we prepared a series of liposomes with a constant 5% DOTAP and varying molar ratios of DSPE-PEG and DOPC (**Fig. R2a**). We then assessed their anti-protein-adsorption properties following exposure to 10% FBS. The results showed that increasing DSPE-PEG to 10% led to noticeable changes in the particle sizes and zeta potentials of the liposomes post FBS exposure (**Fig. R2b, c**), indicating that DOPC possessed a superior anti-protein-adsorption capacity compared to DSPE-PEG. To substantiate this finding, we quantified the proteins adsorbed on the liposomes using a BCA-based assay after incubation in 10% FBS (**Fig. R2d**) and mouse serum (**Fig. R2e**). Consistently, liposomes with more than 10% DSPE-PEG showed significant protein adsorption on their surface, whereas those containing 0-5% DSPE-PEG demonstrated relatively low protein adsorption. These results confirmed the antifouling superiority of the zwitterionic DOPC component in the protein-rich conditions, which was consistent with previous studies[17,18].

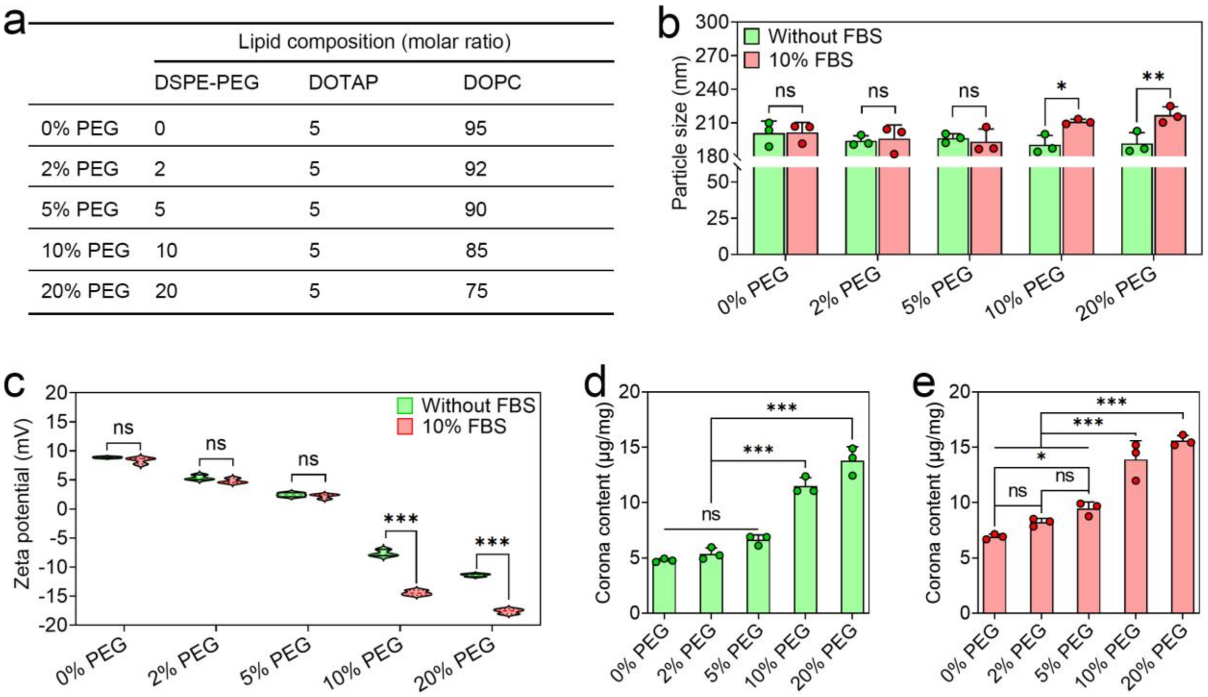

Fig. R2 | Influence of DSPE-PEG on the antifouling effect of AFMFlip. a Composition of liposomes formulated with varying ratios of DSPE-PEG. **b, c** Hydrodynamic sizes (**b**) and zeta potentials (**c**) of liposomes after incubation in the medium without or with 10% FBS (n = 3). **d, e** Determination of protein corona content using the BCA assay in the presence of 10% FBS (**d**) and mouse serum (**e**) (n = 3). ns: $p > 0.05$, $^{**}p < 0.01$, $^{***}p < 0.001$ (two-tailed t-test).

Additionally, we assessed the impact of PEG on the membrane-fusion capacity of AFMFlip using CLSM observation and fluorescence resonance energy transfer (FRET)-based assay. The CLSM images in **Fig. R3a** showed that only the cells treated with liposomes containing 5% DSPE-PEG, 90% DOPC, and 5% DOTAP exhibited DiD (red) signals on the cell membrane, indicating the membrane fusion-mediated uptake. Furthermore, the FRET-based assay demonstrated that the liposomes with a 5% DSPE-PEG ratio achieved optimal membrane-fusion efficiency with cells, even in the presence of FBS (**Fig. R3b**). This result was in accordance with the previous studies, indicating that PEG aids in dehydrating the space between the fusogenic lipid coating of the nanoparticle and the plasma membrane, thereby facilitating lipid fusion in both energetic and structural terms[11]. Overall, the specific combination of DOPC, DOTAP, and DSPE-PEG in the construction of our AFMFlip plays a crucial role in enhancing both its antifouling properties and membrane-fusion efficiency.

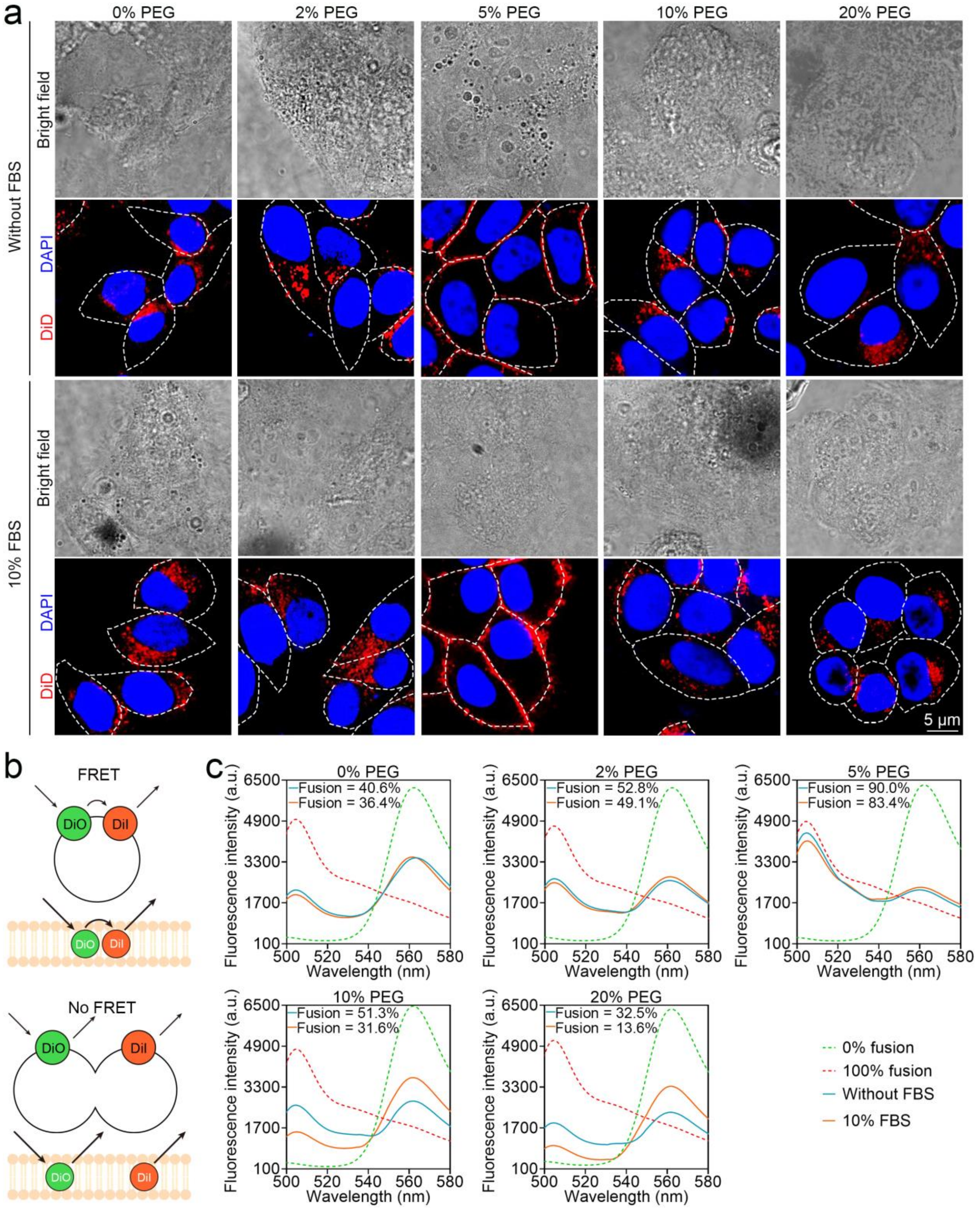

Fig. R3│Evaluation of the impact of DSPE-PEG on the membrane-fusion efficacy of AFMFlip. a Fluorescence microscopy images of HeLa cells treated with different DiD-labeled liposomes without or with 10% FBS for 1 h. The white dotted lines indicate the cellular outlines according to the images of the bright field. **b** Schematic illustration of fluorescence resonance energy transfer (FRET)-based assay to evaluate the liposome-cell fusion. **c** The emission fluorescence spectra of different constructed liposomes labeled with DiO and DiI incubation with HeLa cells for 1 h under the condition without or with FBS at 37 °C. The initial

fluorescence of the labeled liposome suspension was recorded as 0% fluorescence (0% fusion, green lines), and the 100% fluorescence (100% fusion, red lines) was determined after adding Triton X-100 at 0.1% (v/v).

2. The adsorption of hepatocyte-target proteins may contribute to the accumulation of NPs in liver. There is big difference in protein adsorption for AFMFlip and MFlip-a or MFlip-b, why are all three liposomes accumulated in liver in Figure 6b and 6d? Is there any cell type-selectivity for the membrane-fusion of AFMFlip? How to avoid the fusion with the membrane of red blood cells during blood circulation?

Response: Thank you very much for your insightful comment. The *in vivo* distribution of nanoparticles is influenced by several factors, including their intrinsic properties (such as size and surface charge), the nature of the surface protein corona, and the dosage and duration of exposure. Previous studies have demonstrated that nanoparticles larger than 50 nm predominantly accumulate in liver tissue following blood circulation[28,29]. In our study, AFMFlip, MFlip-a, and MFlip-b showed similar hydrodynamic sizes, approximately 200 nm. Meanwhile, the LC/MS-MS analysis showed unnoticeable differences in the components of the corona among these liposomes after FBS incubation (**Fig. R9**, revised from **Fig. 2** in the manuscript). This data suggested the potential of these liposomes to exhibit similar liver accumulation due to the large size.

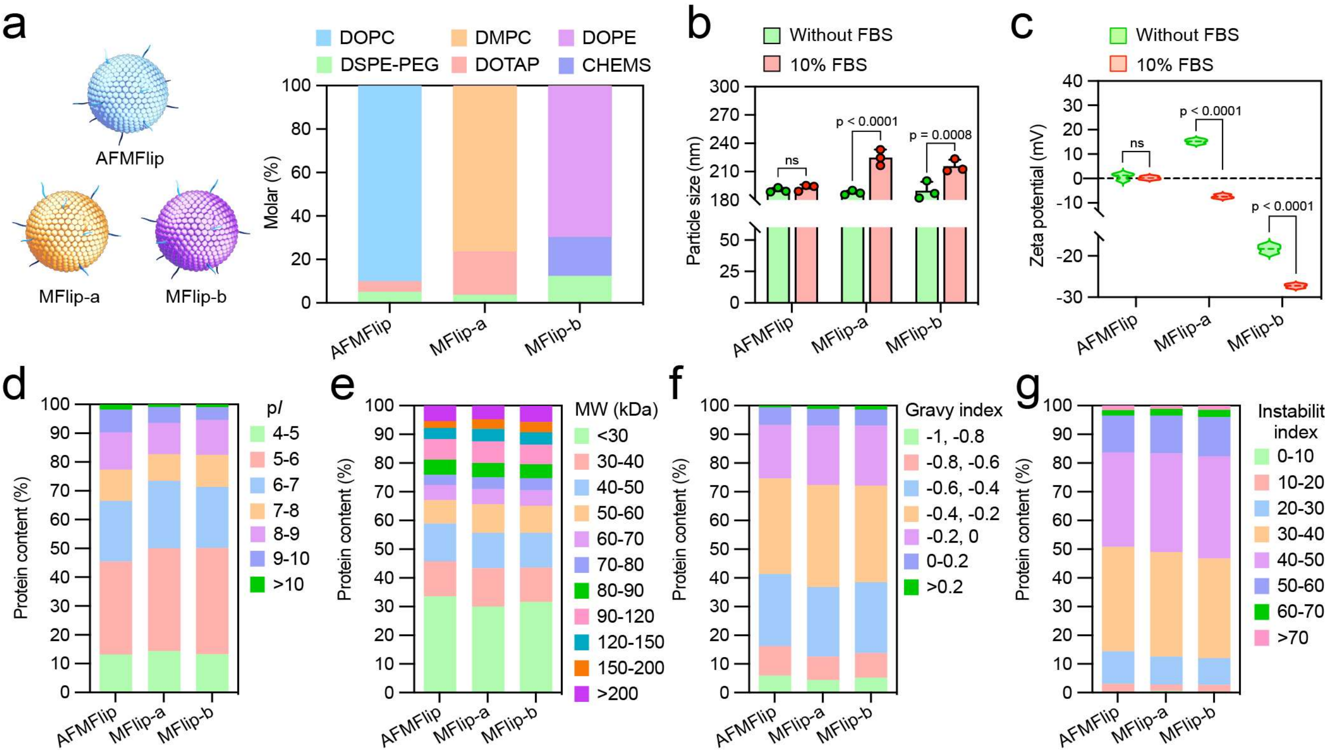

Fig. R9 | Preparation and corona analysis of AFMFlip, MFlip-a, and MFlip-b. a Composite comparison of AFMFlip and the previously reported MFlips (MFlip-a and MFlip-b). **b, c** Hydrodynamic sizes (**b**) and zeta potentials (**c**) of AFMFlip, MFlip-a, and MFlip-b after 1-h incubation in the medium without or with 10% FBS at 37 °C (n = 3). ns: $p > 0.05$, $*p < 0.05$, $**p < 0.01$, $***p < 0.001$ (two-tailed t-test). Measurements were conducted in water at room temperature. **d-g** Classification of corona proteins isolated from AFMFlip, MFlip-a, and MFlip-b after 1-h incubation in a medium containing 10% FBS, using liquid chromatography with tandem mass spectrometry analysis. The identified proteins from corona were classified based on isoelectric point (pI) (**d**), molecular weight (MW) (**e**), gravy index(**f**), and instability index (**g**).

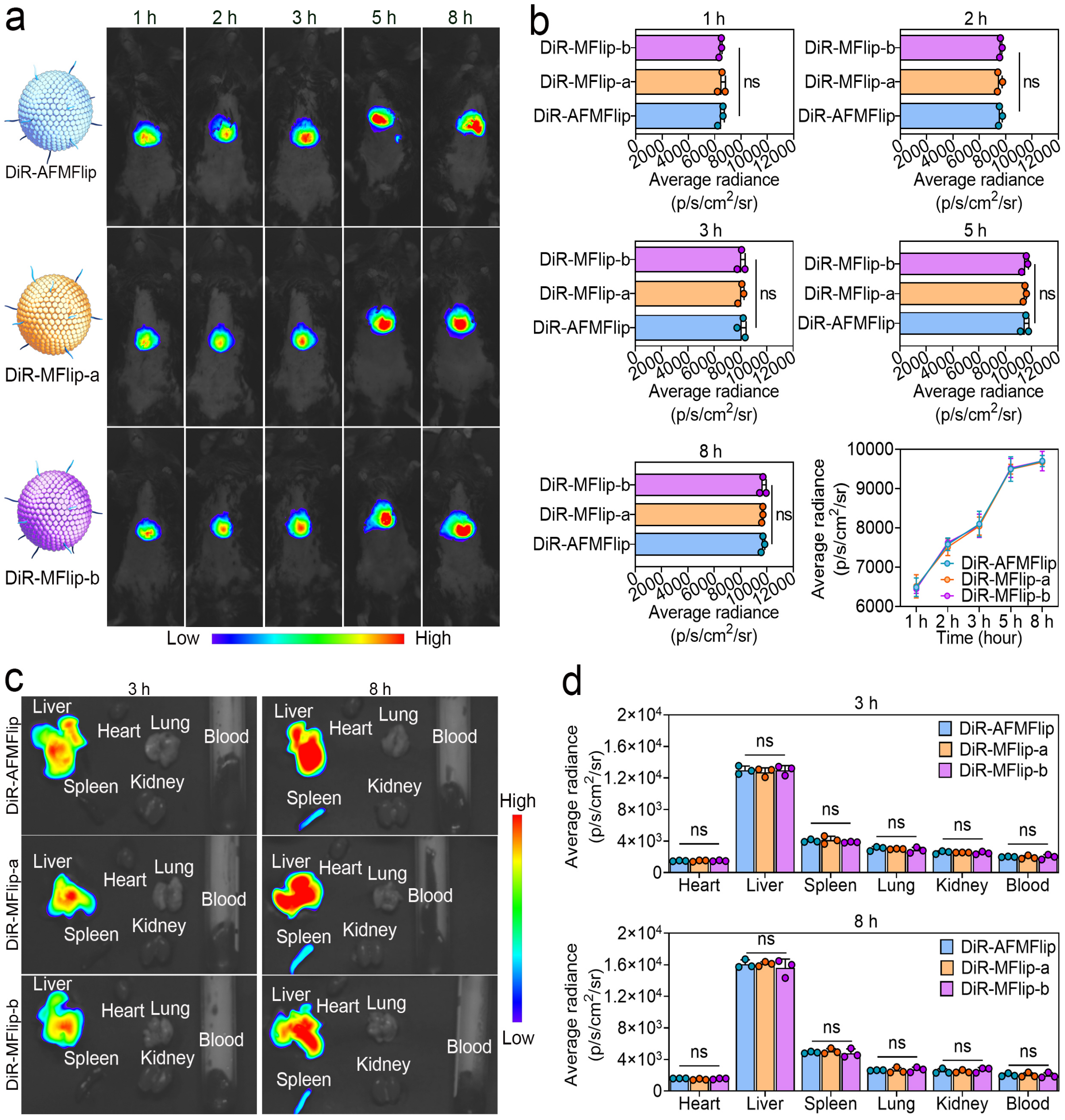

Fig. R10 | *In vivo* distribution of AFMFlip, MFlip-a, and MFlip-b. a, b *In vivo* imaging (**a**) and average intensity analysis (**b**) of mice after receiving an intravenous injection of DiR-

AFMFlip, DiR-MFlip-a, and DiR-MFlip-b (n = 3). ns: $p > 0.05$ (one-way ANOVA). **c, d** Representative *ex vivo* images of distribution (**c**) and corresponding semi-quantitative analysis in major organs (heart, liver, spleen, lung, and kidney) (**d**) from mice at 3 h and 8 h post-injection of DiR-AFMFlip, DiR-MFlip-a, and DiR-MFlip-b (n = 3). ns: $p > 0.05$ (one-way ANOVA).

Fig. 6b and **d** in the manuscript provided the results of the luciferase expression 48 h post-injection, demonstrating the *in vivo* transfection efficiency of the loaded genes. However, these results do not directly indicate the actual *in vivo* biodistribution of liposomes. Therefore, to further investigate the accumulation of the nanoparticles, we labeled AFMFlip, MFlip-a, and MFlip-b with DiR dye and administered them intravenously to mice (**Fig. R10**). The outcomes showed that AFMFlip, MFlip-a, and MFlip-b exhibited similar DiR intensity in the liver section (**Fig. R10a-b**). Additional *ex vivo* imaging confirmed consistent liver tissue accumulation for all three types of liposomes (**Fig. R10c-d**). Interestingly, despite this similar accumulation in the liver, there were differences in the in vivo luciferase transfection efficiency (**Fig. 6b-d**). This highlights the distinctive membrane-fusion-mediated cellular internalization of AFMFlip by hepatic cells, which potentially circumvents endo/lysosomal entrapment, thereby enhancing gene transfection efficiency. These evaluations of the *in vivo* distribution of AFMFlips have been included as Supplementary **Fig. 21** in the revised Supplementary Information. The related descriptions and methods have also been updated and marked in red in the revised manuscript and Supplementary Information.

Notably, these liposomes predominantly accumulated in the liver tissue within 1 h after intravenous injection. Moreover, there was minimal retention of liposomes in the bloodstream during circulation. This observation implies that these liposomes quickly reach liver tissues, minimizing their exposure to blood cells. Such a distribution pattern is advantageous for hepatic intracellular delivery, as it maximizes fusion with liver cells while avoiding interaction with blood cells, thereby enhancing bioavailability for liver-targeted therapies. Consequently, we have chosen AFMFlip as the nanocarrier in our design of nanomedicine aimed at treating liver-related diseases, such as HBV infection (**Fig. 7**).

Moreover, to investigate the cell-type selectivity, we examined the membrane-fusion performance of AFMFlip with different liver cell types (LO2 human hepatic cell line, AML12 mouse hepatic cell line, LX2 human hepatic stellate cell line, and HSC-T6 mouse hepatic stellate cell line) using FRET analysis. Results in **Fig. R11** indicated that all these different cell types exhibited a degree of membrane fusion with AFMFlip, both in the absence and presence

of FBS, confirming the universal membrane-fusion effect of AFMFlip with cells. The lack of selectivity among different cell types could potentially limit the development of precisely targeted membrane-fusion-mediated nanomedicines. The primary goal of this study is to identify a strategy for preserving the membrane-fusion effect of liposomes *in vivo*. Future research will focus on developing membrane-fusion liposomes that can selectively target specific cell types. We have also included a discussion about the limitations of AFMFlip in the "Discussion" section of the revised manuscript. Thank you once again for your professional input.

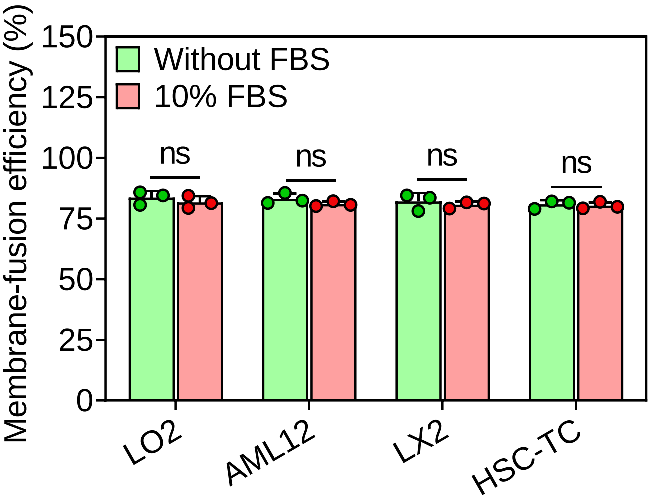

Fig. R11│**Membrane-fusion performance based on FRET analysis of AFMFlips incubated with different liver cell types (n = 3).** ns: $p > 0.05$ (two-tailed t-test).

3. The colocalization confocal imaging of plasma membrane and DiD is needed to confirm the fusion with plasma membrane in Figure 2b.

Response: Thank you very much for your valuable suggestion. Following your advice, we have repeated the experiments in **Fig. 2b** and labeled the cell membrane with CellMask for CLSM observation. As shown in **Fig. R4**, cells treated with NP-2 in the FBS-free medium exhibited the highest colocalization of most DiD (red) signals with the cell membrane (green), suggesting the potential uptake *via* membrane fusion. More importantly, when exposed to the 10% FBS-containing medium, only the cells treated with NP-2 were observed with red-green overlap fluorescence patterns on the cell membrane. In contrast, cells treated with other liposomes showed dotted or dispersed fluorescence signals throughout. To further confirm this observation, the FRET-based membrane-fusogenic quantitative assay (**Fig. R4b**) showed that

NP-2 was the most effective in achieving stable and effective fusion, even in a medium containing 10% FBS. These updated findings have been included in the revised manuscript as **Fig. 2b** and in the Supplementary Information as **Supplementary Fig. 1**.

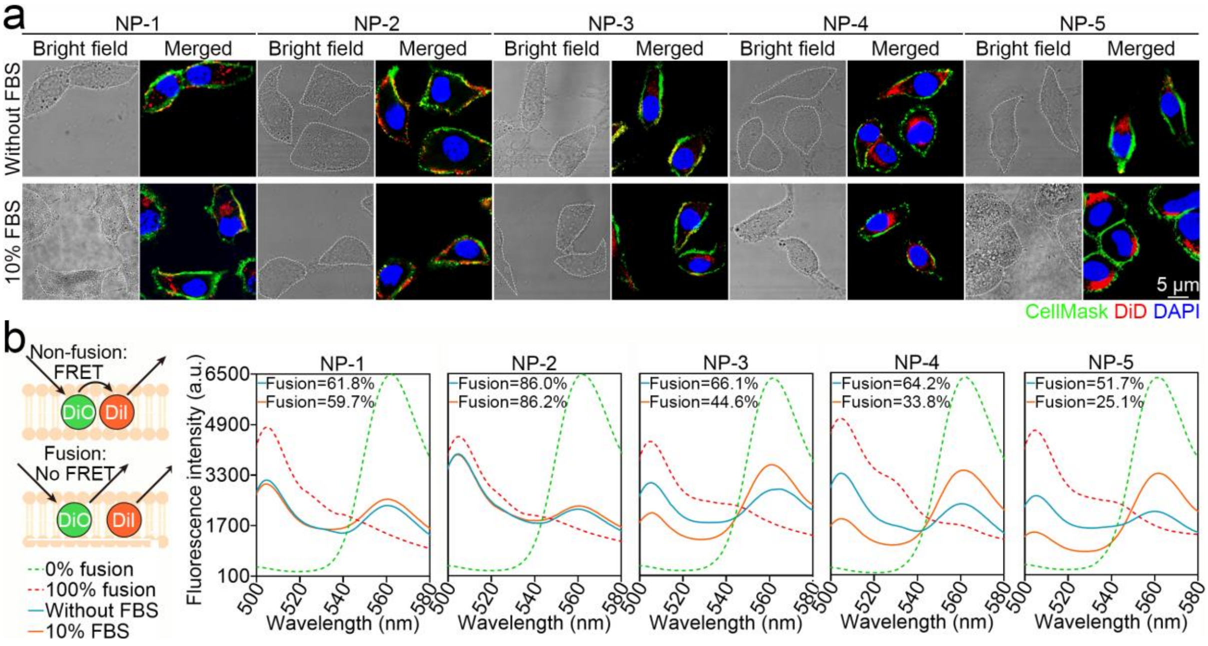

Fig. R4 | Optimization of AFMFlip with serum-resistance membrane-fusion capacity. a CLSM images of liposomes (NP-1 to NP-5 with different lipid component ratios) in the medium with or without 10% FBS. The liposomes were labelled with 1,1'-dioctadecyl-3,3,3',3'-tetramethylindodicarbocyanine,4-chlorobenzenesulfonate salt (DiD). The nuclei were stained with DAPI and the cell membranes were labelled with CellMask. **b** FRET analysis to evaluate the membrane-fusion efficacy after 1 h incubation with cells in the medium with or without 10% FBS at 37 °C.

4. There is not enough information of NP-1…NP-5 in Figure 2b and these NPs were not discussed in the Results part. The author claimed that "cells treated with NP-2 and NP-3 in the FBS-free medium were observed with DiD (red) signals uniformly distributed on the cell membrane". I don't believe NP-3 clearly showed the uniform distribution around the nucleus.

Response: Thank you very much for your professional guidance. The details of NP-1 to NP-5 have already been included in Supplementary Table 1 (Table R1). For the experiment in **Fig. 2b**, we have captured clearer CLSM images and conducted a FRET-based membrane-fusogenic quantitative assay to further support the conclusions, as displayed in **Fig. R4**. Additionally, we have enriched the discussion section with a more detailed description in the related part as follows:

"These liposomes with varying molar ratios of DOPC, DOTAP, and DSPE-PEG showed similar hydrodynamic sizes of approximately 200 nm and favorable polymer dispersity indexes (PDI) below 0.25. Concurrently, the zeta potentials increased progressively from -7.5 mV to 19.2 mV with the increasing ratios of the positively-charged DOTAP (Supplementary Table 1). These MFlips were labeled with 1,1'-dioctadecyl-3,3,3',3'-tetramethylindodicarbocyanine,4-chlorobenzenesulfonate salt (DiD) and introduced to HeLa cells in the culture medium without or with 10% FBS, followed by confocal laser scanning microscopy (CLSM) imaging. As shown in **Fig. 2b** and Supplementary **Fig. 1a**, cells treated with NP-2 in FBS-free medium showed that most of DiD (red) signals were uniformly distributed on the CellMask-labeled cell membrane (green) to yield yellow overlaps, suggesting the potential uptake *via* membrane fusion. More importantly, when exposed to the 10% FBS-containing medium, only cells treated with NP-2 were observed with the red-green colocalized fluorescence patterns on the cell membrane. In contrast, cells treated with other liposomes showed either dotted or spread red fluorescence signals throughout. To further substantiate these observations, the liposomes were labeled with both 3,3'-dioctadecyloxacarbocyanine perchlorate (DiO) and 1,1'-dioctadecyl-3,3,3',3'-tetramethylindocarbocyanine perchlorate (DiI), followed by incubation with HeLa cells. The membrane-fusion efficiency was then quantified using a fluorescence resonance energy transfer (FRET)-based assay (Supplementary **Fig. 1b**). The results indicated that NP-2 achieved the highest membrane fusion efficiency in both 10% FBS-free and -containing media."

Table R1. The five AFMFlip systems (NP-1 to NP-5) synthesized with different molar ratios of lipid components.

	Lipids (molar ratio)	Size (nm)	PDI	Zeta potential (mV)
NP-1	DSPE-PEG/DOPC/DOTAP (5/95/0)	187.4 ± 3.4	0.15 ± 0.01	-7.5 ± 1.3
NP-2	DSPE-PEG/DOPC/DOTAP (5/90/5)	190.5 ± 2.6	0.18 ± 0.01	1.2 ± 2.6
NP-3	DSPE-PEG/DOPC/DOTAP (5/85/10)	193.0 ± 1.3	0.20 ± 0.01	3.7 ± 1.7
NP-4	DSPE-PEG/DOPC/DOTAP (5/75/20)	199.5 ± 3.0	0.13 ± 0.02	11.2 ± 2.3
NP-5	DSPE-PEG/DOPC/DOTAP (5/65/30)	185.9 ± 2.4	0.22 ± 0.01	19.2 ± 1.2

5. Lipo2k forms NPs or nanocomplexes with plasmids, but not liposomes. It is not a good control to show the effect of FBS dependent fusion-endocytosis switch on the delivery efficacy of pDNA. Delivery with MFlip-a or MFlip-b should be performed for this purpose.

Response: Thank you very much for your valuable suggestion. We have supplemented MFlip-a^{pEGFP} and MFlip-b^{pEGFP} treatment groups as the comparison to evaluate their delivery efficiencies (**Fig. R12**).

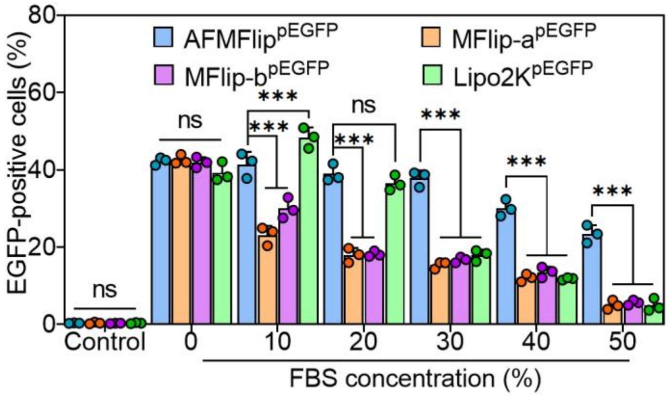

Fig. R12 | EGFP transfection efficiency of AFMFlip^{pEGFP}, MFlip-a^{pEGFP}, MFlip-b^{pEGFP}, and Lipo2K^{pEGFP} in the medium containing 0-50% FBS, analyzed *via* FACS (n = 3). ns: $p > 0.05$, ***$p < 0.001$ (one-way ANOVA).

The results in **Fig. R12** showed that the EGFP-transfection efficiency of MFlip-a^{pEGFP} and MFlip-b^{pEGFP} decreased sharply with the increase of FBS concentration. Additionally, we have added this result to **Fig. 4e** in the revised manuscript and corrected the description in the related section as follows:

1) "Owing to this excellent protein-resistance membrane-fusion-mediated transportation, AFMFlip could achieve a steadily enhanced green fluorescence protein (EGFP)-encoding plasmid (pEGFP) transfection (nearly 40% efficiency) until the FBS concentration rose to 30%, at which the transfection capacities of MFlip-a, MFlip-b, and commercial Lipofectamine 2000 (Lipo2K) significantly reduced to 15.4%, 16.7%, and 17.9%, respectively (**Fig. 4e**)";

2) Caption for **Fig. 4e**: "**e** EGFP transfection efficiency of AFMFlip^{pEGFP}, MFlip-a^{pEGFP}, MFlip-b^{pEGFP}, and Lipo2K^{pEGFP} in the medium containing 0-50% FBS, analyzed *via* FACS (n =3). ns: $p > 0.05$, ***$p < 0.001$ (one-way ANOVA)."

6. The statistical analysis should be performed for the groups of AFMFlip and MFlip-a or MFlip-b in Figure 5b.

Response: Thank you very much for your meticulous review and considerate reminder. We have conducted the necessary statistical analysis and updated the corresponding figures, shown in **Fig. R13** (**Fig. 5b** in the updated manuscript).

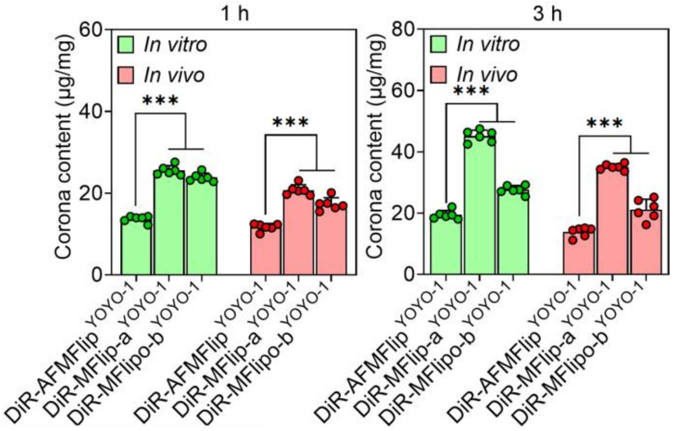

Fig. R13 | The BCA analysis for protein quantification. The *in vitro* mimicking corona was formed by incubating nanoparticles with 10% mouse serum (n = 5). ***$p < 0.001$ (one-way ANOVA).

7. There are mistakes in a, b, c…. numbering in the caption of Figure 4.

Response: Thank you very much for your attentive review and helpful reminder. We have meticulously revised the numbering in the caption of **Fig. 4** in the updated version of our manuscript. Additionally, we have thoroughly reviewed both our manuscript and Supplementary Information to identify and correct any similar errors. Your guidance has been instrumental in enhancing the accuracy and clarity of our work.

Response to Reviewer 3

The authors provide an interesting report on a fusogenic liposome formulation with lower protein-fouling than previous examples and improved fusion with cells at higher serum content and including two *in vivo* examples. However, before publication, there are several major and minor considerations to be taken into account.

Response: Thank you very much for your positive evaluation and insightful comments. We have thoughtfully addressed your questions for revision, as detailed below.

1. Can the authors please justify and put in literature context their choice of lipids for AFMFlip with respect to fusion (not the antifouling argument, which is understandable for DOPC/DSPE-PEG-containing liposomes)? Is DOPC/DOTAP/DSPE-PEG not a more standard liposome formulation, rather than a fusogenic one (incorporation of DOPE for fusion is standard but not DOPC)? For example, in papers by Agnes Csiszár *et al*. it was shown that DOTAP/DOPC shows very low fusion and adding DSPE-PEG into a fusogenic version (DOTAP/DOPE) did actually remove the fusogenic property:

1). https://www.ncbi.nlm.nih.gov/pmc/articles/PMC5855568/#sec2-ijms-19-00346

2). https://www.mdpi.com/2079-4991/9/7/1025

Response: We appreciate your professional comments. Our MFlips design draws inspiration from Professor Michael J. Sailor's study, where MFlips are crafted from three specific lipid components (at a specific ratio): neutral lipid, cationic lipid, and PEGylated lipid[10,11]. The neutral lipid should have a relatively low phase transition temperature (< 37 °C) and constitute a major fraction of the liposome, allowing the lipids to be in a fluidic liquid crystal phase (L_α) *in vivo*. The cationic lipid imparts a positive charge to facilitate cellular uptake and fusion through the electrostatic attraction with the anionic plasma membrane. The PEGylated lipid assists in dehydrating the space between the fusogenic lipid coating of the nanoparticle and the plasma membrane, thereby facilitating lipid fusion in both energetic and structural terms. According to this experience, they developed a membrane-fusogenic liposome (*i.e.* MFlip-a in our manuscript) using DMPC, DOTAP, and DSPE-PEG at a specific ratio of 76.2/20/3.8 and applied it for intracellular drug delivery. However, the positive charge on MFlip-a's surface often results in a diminished membrane-fusion effect in protein-rich environments due to protein corona formation on its surface[5]. Therefore, our goal is to innovate an antifouling

membrane-fusogenic liposome capable of achieving fusion with cell membranes even in protein-enriched conditions, thereby enhancing *in vivo* intracellular delivery.

Informed by their insights, we selected DOPC, a neutral lipid known for its antifouling properties and a phase transition temperature of -20 °C[30], to replace the DMPC in MFlip-a. Subsequently, we embarked on identifying a novel composition and ratio for MFlips. Our efforts led to the discovery of a unique lipid ratio of 90/5/5 (DOPC/DOTAP/DSPE-PEG), distinct from that of MFlip-a, which demonstrated effective membrane-fusion cell internalization even in the protein-riched environments (**Fig. R4**, revised as **Fig. 2b** and **Supplementary Fig. 1** in the updated version). This variation in fusogenic lipid ratio is likely attributed to the unsaturated lipid tails and the longer alkyl chain in the DOPC, which both contribute to regulating the fluidity of the lipid coating and enhancing its interaction with plasma membrane[24-27]. Due to these characteristics, AFMFlip, rich in DOPC, requires less DOTAP for engaging with the cell membrane to initiate fusion, thus preserving a higher proportion of zwitterionic groups on its surface. Consequently, our design of AFMFlip with a reduced amount of cationic DOTAP effectively induces membrane fusion, particularly in high-serum environments, while maintaining its antifouling efficacy.

DOPE is indeed a traditional and widely utilized component in the construction of fusogenic liposomes. Agnes Csiszár *et al.* reported that liposomes composed solely of DOPE and DOTAP at a ratio of 1/1, without incorporating DSPE-PEG, can fuse with cell membranes. This specific fusogenic composite and ratio were also endorsed by Prof. Zhou[8], who identified and confirmed that a 1/1 ratio of DOPE/DOTAP is optimal for fusion. Additionally, Agnes Csiszár *et al.* also claimed that DOPE/DOTAP liposomes exhibit higher fusion efficiency compared to DOPC/DOTAP liposomes at the same ratio of 1/1[12]. However, we think this conclusion might lack rigor, as the optimal molar ratios for DOPC/DOTAP, which could be crucial for optimal fusion, were not fully explored in their study. In our research, we have optimized an AFMFlip with a specific lipid ratio (DOPC/DOTAP/DSPE-PEG = 90/5/5) that demonstrates remarkably high membrane fusion efficiency, nearly 90% (**Table R1** and **Fig. R4**). This finding addresses the research gap in DOPC-based MFlips. Moreover, more recent studies have developed fusogenic liposomes incorporating DOPC. [14,31-33], further supporting the premise that DOPC, with an optimized ratio, is a viable component for achieving membrane fusion.

Regarding DSPE-PEG, Agnes Csiszár *et al.* did not investigate its influence on membrane fusion. However, recent studies have indicated that PEGylated lipid components can facilitate membrane fusion by creating a fully hydrated, flexible cushion between lipid bilayers[34,35]. This mechanism is thought to interact with cellular membranes in a way akin to the endogenous

soluble *N*-ethylmaleimide-sensitive-factor attachment receptor (SNARE) protein[10,11]. Notably, a recent study reported a fusogenic liposome that incorporated PEG into a DOTAP/DOPE-based fusogenic composition. This newly formulated liposome demonstrated highly efficient fusogenic properties, achieving fusion rates as high as 84.3%[8]. Overall, these studies support our assertion that the addition of PEG positively contributes to enhancing membrane fusion.

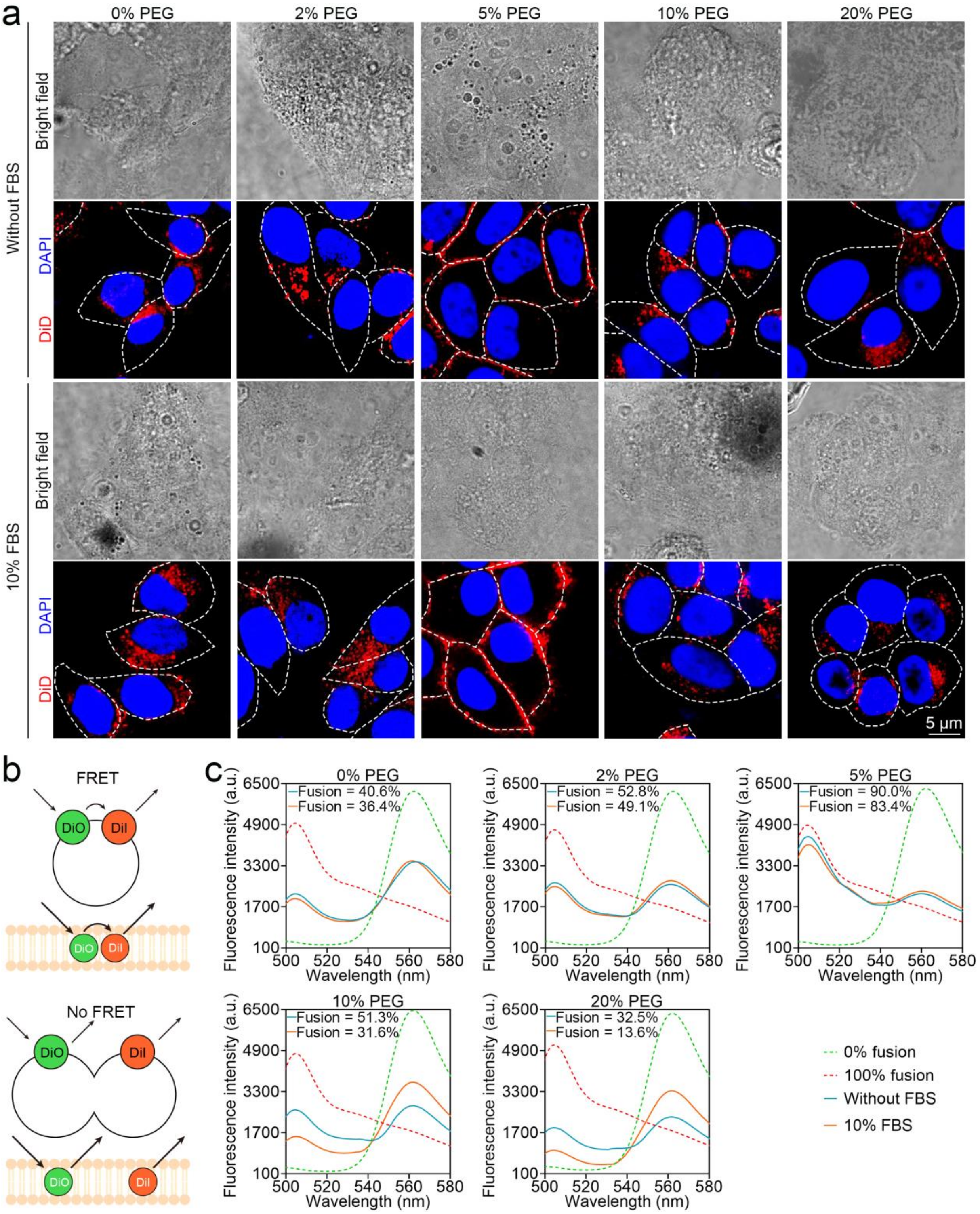

Fig. R3 | Evaluation of the impact of DSPE-PEG on the membrane-fusion efficacy of

AFMFlip. a Fluorescence microscopy images of HeLa cells treated with different DiD-labeled liposomes without or with 10% FBS for 1 h. The white dotted lines indicate the cellular outlines according to the images of the bright field. **b** Schematic illustration of fluorescence resonance energy transfer (FRET)-based assay to evaluate the liposome-cell fusion. **c** The emission fluorescence spectra of different constructed liposomes labeled with DiO and DiI incubation with HeLa cells for 1 h under the condition without or with FBS at 37 °C. The initial fluorescence of the labeled liposome suspension was recorded as 0% fluorescence (0% fusion, green lines), and the 100% fluorescence (100% fusion, red lines) was determined after adding Triton X-100 at 0.1% (v/v).

We also evaluate the influence of PEG on the membrane-fusion capacity of AFMFlip using CLSM observation and fluorescence resonance energy transfer (FRET)-based assay. The CLSM images in **Fig. R3a** showed that only the cells treated with liposomes containing 5% DSPE-PEG, 90% DOPC, and 5% DOTAP exhibited DiD (red) signals on the cell membrane, indicating the membrane fusion-mediated uptake. The FRET-based assay further demonstrated that liposomes with a 5% DSPE-PEG ratio achieved optimal membrane-fusion efficiency with cells, even in conditions containing FBS (**Fig. R3b, c**). This finding aligns with previous studies, which suggest that PEG aids in dehydrating the space between the fusogenic lipid coating of the nanoparticle and the plasma membrane, thereby facilitating lipid fusion in both energetic and structural terms [10,11]. Overall, the specific ratio of DOPC, DOTAP, and DSPE-PEG in our AFMFlip formulation plays a crucial role in enhancing both its antifouling properties and membrane-fusion efficiency.

2. Structural characterization, e.g. cryoTEM, is missing, hence, the authors do not currently know whether their formulations (+/- plasmid) are liposomes or LNPs.

Response: Thank you very much for your constructive comment. Liposomes are typically described as closed lipid bilayer vesicles[36,37], whereas lipid nanoparticles (LNPs) are colloidal particles composed of a lipid matrix stabilized with surfactants[38]. The preparation of LNPs generally involves dispersing melted lipids into a warm aqueous phase containing surfactants, using high-shear homogenization techniques such as high-pressure homogenization or the microemulsion method[39]. In contrast, our nanoparticles were formulated using the film hydration method (described in the section of Method). This method entails hydrating a thin lipid film with a sterile deionized water solution, making it well-suited for liposome preparation.

Furthermore, we have included TEM characterization in **Fig. R14** (the updated Supplementary **Fig. 11a**) to display the vesicle structures of our MFlips without plasmid loading and the core-shell structures of MFlips with $CaCO_3$/plasmids encapsulation. This visual evidence has been added to the updated version of our manuscript, with corresponding textual revisions highlighted in red.

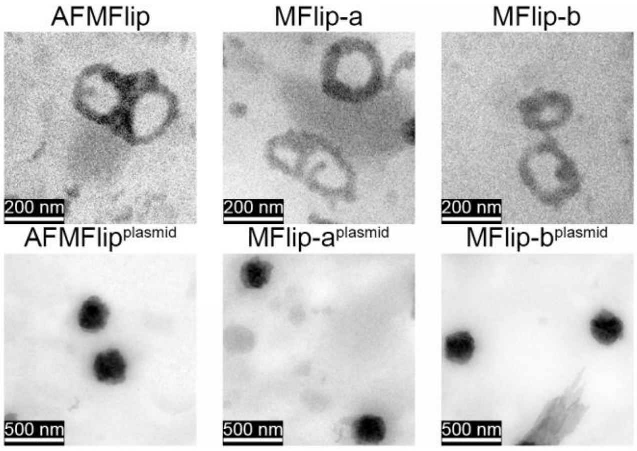

Fig. R14 | TEM images of AFMFlip, MFlip-a, and MFlip-b with and without the loading of $CaCO_3$/plasmid nanoparticles.

3. Discussion on the relevance of protein corona needs to be changed and extended. The authors characterised nicely the protein corona forming on their three nanoparticle versions, including on AFMFlip. Hence, the more efficient liver accumulation of AFMFlip is not due to an absence of a corona (antifouling would suggest this) but more likely due to the specific proteins that accumulated on the AFMFlip surface. E.g. ApoE accumulation on LNPs has previously been shown to be responsible for hepatocyte uptake. The authors show here that ApoB is one of the main corona proteins (also on AFMFlip), what is the relevance of this? And how does their formulation compare to the well-known ionizable lipid-based nanoparticles (LNPs) previously used in some of the Covid vaccines, and for liver-targeted nucleic acid delivery, *e.g.* Onpattro. What is the advantage of their formulation over these others?

Response: Thank you very much for your detailed and insightful comments. According to the previous studies, the *in vivo* biodistribution of nanoparticles is dependent on the nanoparticle properties (size, surface charge, etc.), surface protein corona composites, and the exposure dose and duration. Numerous studies have demonstrated that nanoparticles larger than 80 nm predominantly accumulate in the liver tissue after blood circulation[28]. Our AFMFlip, MFlip-a, and MFlip-b exhibited similar hydrodynamic sizes, approximately 200 nm (**Fig. R9**, revised

from **Fig. 2**). Additionally, LC/MS-MS analysis revealed minimal differences in the types of proteins adsorbed by these three liposomes following FBS incubation. Consequently, we hypothesize that their *in vivo* accumulation in the liver is primarily influenced by nanoparticle size, rather than the specific proteins adsorbed on their surface. To explore their biodistribution, we labeled AFMFlip, MFlip-a, and MFlip-b with DiR dye and administered them intravenously to mice (**Fig. R10**). The results indicated that AFMFlip, MFlip-a, and MFlip-b all showed similar DiR signal intensities in liver sections (**Fig. R10a-b**). Further *ex vivo* imaging confirmed comparable liver tissue accumulation for all three liposome types (**Fig. R10c-d**). H Despite this similar liver accumulation, mice treated with AFMFlip exhibited the highest plasmid expression for luciferase 48 h post-injection (**Fig. 5**), underscoring the delivery efficacy of AFMFlip through membrane fusion with liver cells.

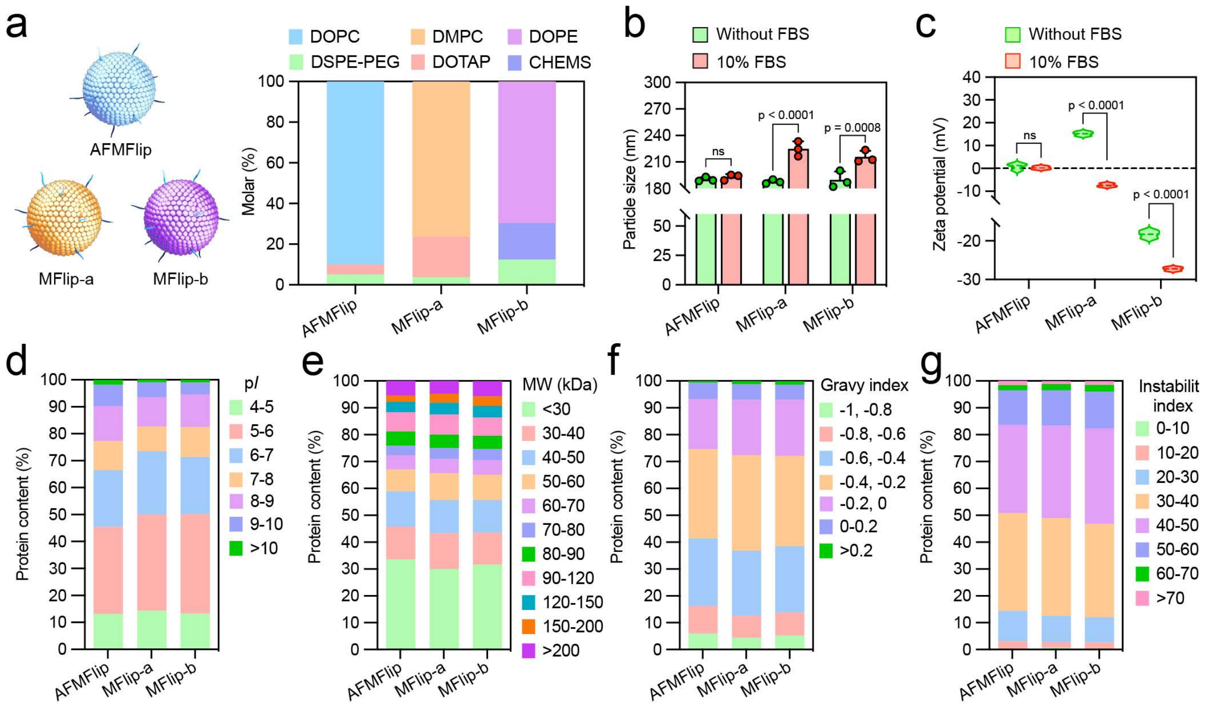

Fig. R9│ Preparation and corona analysis of AFMFlip, MFlip-a, and MFlip-b. a Composite comparison of AFMFlip and the previously reported MFlips (MFlip-a and MFlip-b). **b, c** Hydrodynamic sizes (**b**) and zeta potentials (**c**) of AFMFlip, MFlip-a, and MFlip-b after 1-h incubation in the medium without or with 10% FBS at 37 °C (n = 3). ns: $p > 0.05$, *$p < 0.05$, **$p < 0.01$, ***$p < 0.001$ (two-tailed t-test). Measurements were conducted in water at room temperature. **d-g** Protein classification for corona proteins recovered from AFMFlip, MFlip-a, and MFlip-b after 1-h incubation in 10% FBS-containing medium by liquid chromatography with tandem mass spectrometry analysis. The identified proteins from corona

were classified based on isoelectric point (pI) (**d**), molecular weight (MW) (**e**), gravy index(**f**), and instability index (**g**).

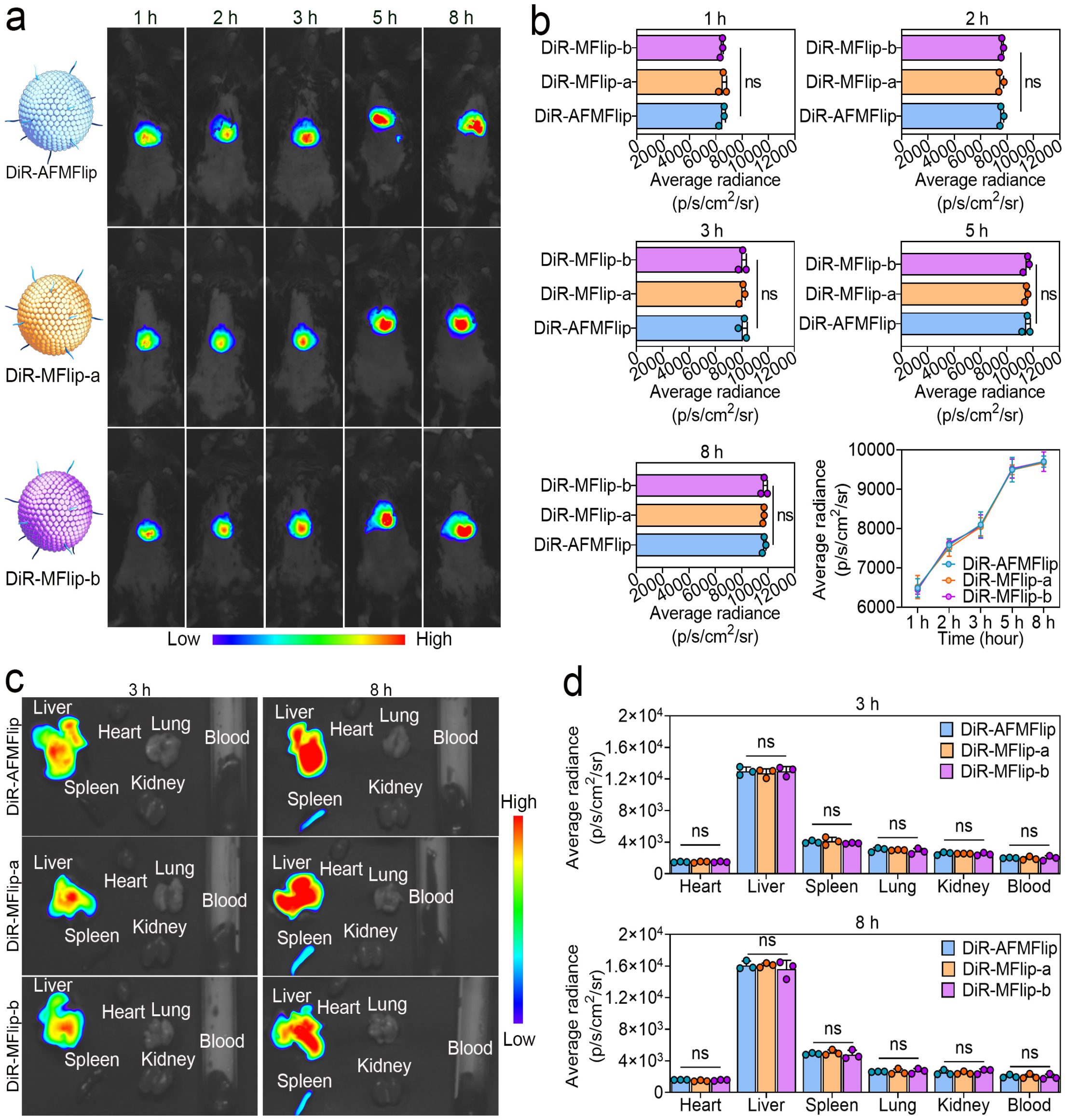

Fig. R10 | *In vivo* distribution of AFMFlip, MFlip-a, and MFlip-b. a, b *In vivo* imaging (**a**) and average intensity analysis (**b**) of mice after receiving an intravenous injection of DiR-AFMFlip, DiR-MFlip-a, and DiR-MFlip-b (n = 3). ns: $p > 0.05$ (one-way ANOVA). **c, d** Representative *ex vivo* images of distribution (**c**) and corresponding quantification analysis in major organs (heart, liver, spleen, lung, and kidney) (**d**) from mice at 3 h and 8 h post-injection of DiR-AFMFlip, DiR-MFlip-a, and DiR-MFlip-b (n = 3). ns: $p > 0.05$ (one-way ANOVA).

To explore the distinctions between AFMFlip and commercial lipid nanoparticles (LNPs), we established the MC3-LNP (an FDA-approved ionizable amide lipid used in ONPATTRO) and SM102-LNP (an FDA-approved ionizable amide lipid for Covid vaccines), both loaded

with plasmids as per established protocols (**Fig. R15a, b**)[40,41]. These plasmid cargos were then labeled with YOYO-1, creating AFMFlip^{YOYO-1}, MC3-LNP^{YOYO-1}, and SM102-LNP^{YOYO-1}. Following incubation with cells, we observed their intracellular trafficking. CLSM images showed that plasmids delivered by AFMFlip were not entrapped in lysosomes, even with the disruption of FBS. Conversely, cells treated with MC3-LNP^{YOYO-1} and SM102-LNP^{YOYO-1} displayed green-magenta fluorescence overlap, indicating lysosomal retention (**Fig. R15c**). To further investigate the cell-internalization mechanism, these nanoparticles were incubated with HeLa cells that were pretreated with the inhibitors targeting various uptake pathways. The results indicated that AFMFlip's uptake was predominantly hindered by Z-Phe-Phe-Phe-OH (membrane fusion inhibitor), but not by other endocytosis inhibitors (**Fig. R15d**). In contrast, the cellular uptake of MC3-LNP and SM102 was significantly affected by endocytic inhibitors like chlorpromazine, amiloride, and nystatin (**Fig. R15e, f**). Overall, AFMFlip demonstrated a unique membrane fusion delivery route, distinct from the endocytosis observed in well-known lipid formations (MC3-LNP and SM103-LNP). This membrane-fusion-mediated cell internalization circumvents endo/lysosomal entrapment, enabling more direct and rapid cargo release in the cytoplasm, potentially leading to more efficient delivery. Nonetheless, further enhancements are required to optimize AFMFlip's performance.

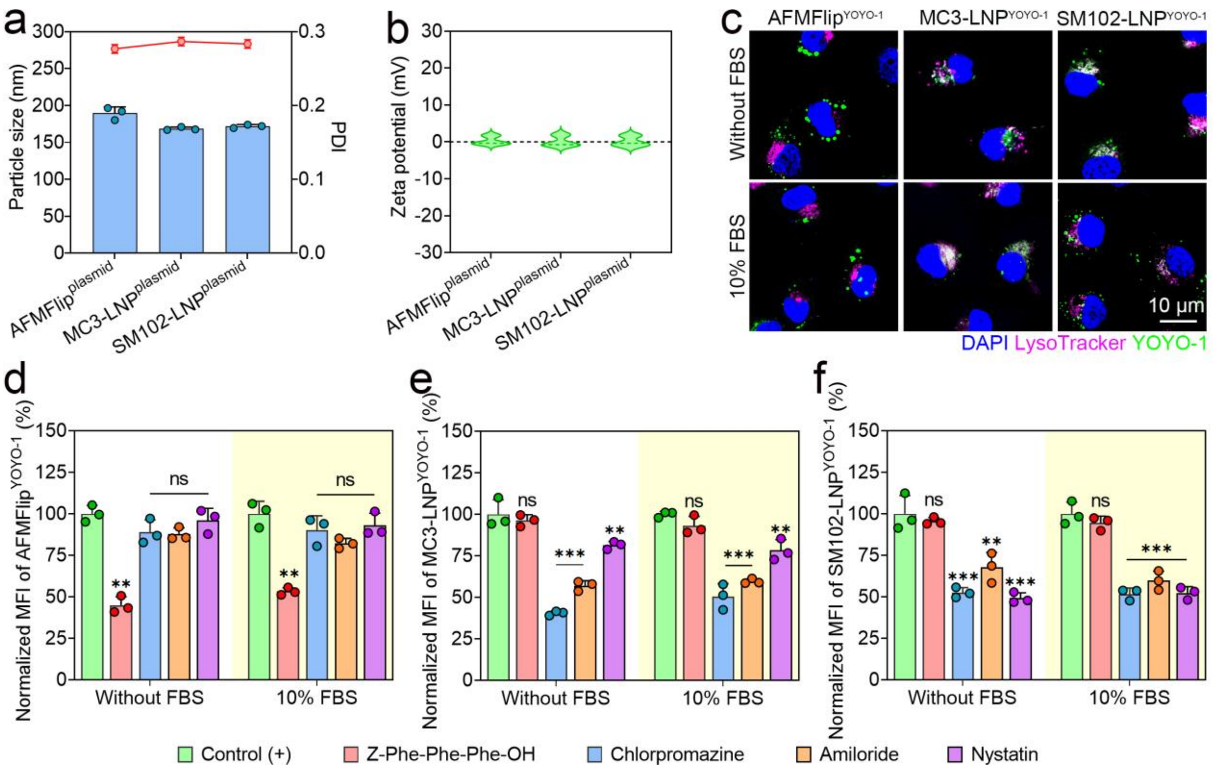

Fig. R15 | Characterization and intracellular trafficking of AFMFlip, MC3-LNP, and SM102-LNP for plasmid delivery. a, b DLS analysis (**a**) and zeta potentials (**b**) of AFMFlip,

MC3-LNP, and SM102-LNP loaded with plasmid cargos (n = 3). **c** CLSM observation of HeLa cells co-incubated with AFMFlip^{YOYO-1}, MC3-LNP^{YOYO-1}, and SM12-LNP^{YOYO-1} in the medium without or with 10% FBS, showing their intracellular trafficking process. **d-f** Fluorescence-activated cell sorting (FACS) analysis of HeLa cells after treatment with AFMFlip^{YOYO-1} (d, revised from **Fig. 3c**), MC3-LNP^{YOYO-1}, and SM102-LNP^{YOYO-1} for 2 h in the FBS-free and 10% FBS-containing medium. Cells were pretreated with the membrane-fusion (Z-Phe-Phe-Phe-OH) or endocytosis (chlorpromazine, amiloride, and nystatin) inhibitors at designed concentrations for 30 min at 37 °C (n = 3). ns: $p > 0.05$, *$p < 0.05$, **$p < 0.01$ (one-way ANOVA).

4. Antifouling would suggest there is no/very little protein binding to the surface, I suggest calling it low fouling, as the authors still found quite a lot of protein binding also on AFMFlip (see Fig.2g). Throughout the text, statements like "AFMFlip could effectively retain membrane-fusion-dependent behavior in the presence of serum protein-containing medium, resulting from the ability to prevent protein corona formation" are not entirely accurate, as the authors have detected protein coronas on all three particle versions. Yes, AFMFlip binds less protein, but not non. Also, fusion is maintained up to 38% FBS, which is great, but physiologically, there is 100% FBS in serum; this should be discussed in more detail.

Response: We appreciate your constructive comments to improve the clarity of our statements. Drawing from existing literature, the term "antifouling capacity" is understood as a relative measure, characterized by a significantly reduced, though not completely absent, protein corona formation[42]. In this context, we assert that AFMFlip exhibits a notably efficient antifouling capacity, outperforming previously reported membrane fusogenic liposomes. In line with your recommendation, we have amended the relevant descriptions in our manuscript. Specifically, phrases like "prevent protein corona formation" have been modified to "alleviate protein corona formation" to more accurately reflect this relative capacity.

Additionally, we have further investigated the antifouling and membrane-fusion capabilities of AFMFlip *in vivo*, using mouse models. Upon intravenous administration in mice, AFMFlip demonstrated remarkable antifouling properties and displayed more pronounced membrane-fusion-mediated intracellular transport compared to the conventional MFlip-a and MFlip-b (**Fig. 5**), thereby achieving better delivery efficiency (**Fig. 6** and **Fig. 7**). Additionally, we have provided an in-depth discussion on the opportunities and challenges associated with *in vivo* applications as follows: Moreover, the protein-resistant membrane-fusion-mediated delivery

efficiency of AFMFlips can also be optimized by combining other anti-protein-adsorption molecules with superior antifouling effect.

5. For Fig.2b and 3a (also 5e), for the comparison of membrane fusion for various formulations, more than just a single/few cell/s per image is needed. Overview images with many cells should be provided in the SI at least. How did the authors obtain the dotted cell outline in Fig.2b? From these images, I cannot clearly see whether there is membrane fusion or not. Also, providing images for longer than 1h of incubation would clarify whether this was just membrane association, leading to dotted structures intracellularly a bit later, or whether this is actual membrane fusion. Using an additional membrane stain for identification (e.g. WGA) could help with conclusions too. For Fig.5e, the plasmid signal appears to be quite weak, do the authors have a negative control without plasmid to ensure this is not background autofluorescence?

Response: Thank you very much for your thorough review and professional comments. For **Fig. 2b**, we have repeated the experiment and enhanced our methodology by staining the cell membrane and capturing a larger number of cells for improved visualization. As shown in the CLSM images in **Fig. R4a** (revised as **Fig. 2b** and **Supplementary Fig. 1** in the updated version), cells treated with NP-2 in the FBS-free medium predominantly exhibit a uniform distribution of DiD (red) signals on the CellMask-labeled cell membrane (green), resulting in yellow overlaps. This pattern suggests potential uptake through membrane fusion. Notably, in the medium containing 10% fetal bovine serum (FBS), only the cells treated with NP-2 displayed red-green colocalized fluorescence patterns, in stark contrast to other liposomes which showed dotted or dispersed red fluorescence signals throughout the cell. To substantiate this observation, we employed a FRET-based assay to quantify the membrane fusion efficiency. Results in **Fig. R4b** showed that NP-2 achieved the highest efficiency in both FBS-containing and FBS-free media. This indicates that NP-2, formulated with a molar ratio of 90/5/5 (DOPC/DSPE-PEG/DOTAP), has the potential to retain the fusion behavior in the protein-rich conditions. We have incorporated these findings as **Fig. 2b, c** in the updated manuscript and **Supplementary Fig. 1** in the Supplementary Information. Corresponding updates have also been made to the text and Methods section, highlighted in red for clarity.

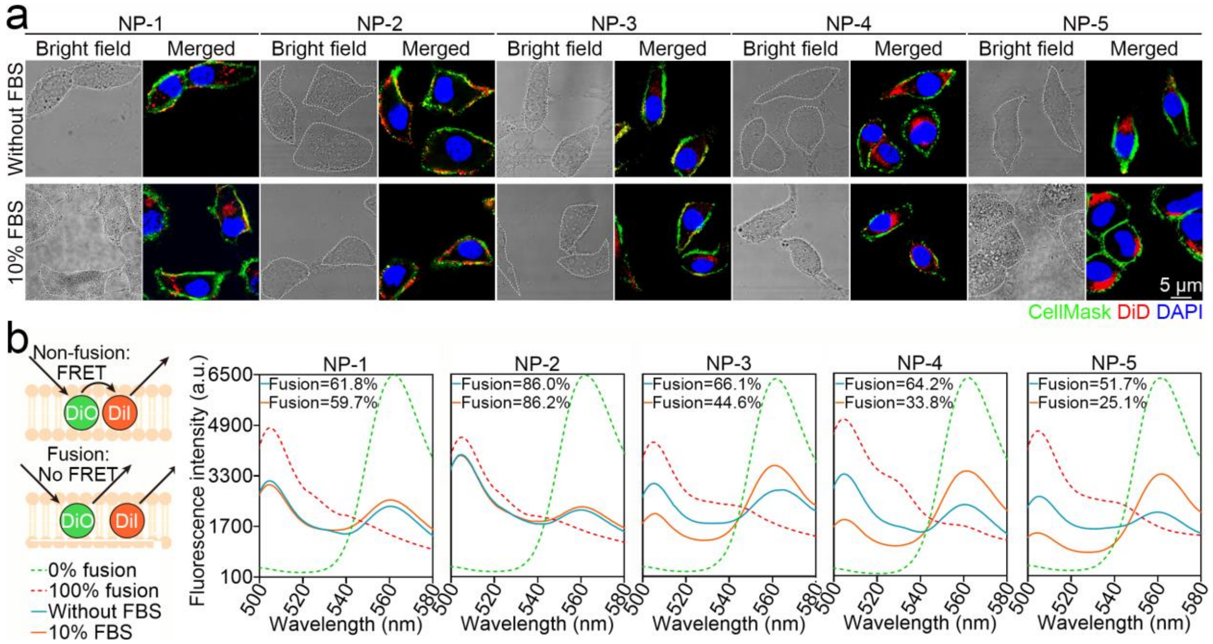

Fig. R4 | Optimization of AFMFlip with serum-resistance membrane-fusion capacity. a CLSM images of liposomes (NP-1 to NP-5 with different lipid component ratios) in the medium with or without 10% FBS. The liposomes were labelled with 1,1'-dioctadecyl-3,3,3',3'-tetramethylindodicarbocyanine,4-chlorobenzenesulfonate salt (DiD). The nuclei were stained with DAPI and the cell membranes were labelled with CellMask. **b** FRET analysis to evaluate the membrane-fusion efficacy after 1 h incubation with cells in the medium with or without 10% FBS at 37 °C.

We also supplemented the data in **Fig. 3a** with additional cell membrane staining in the analysis. As shown in **Fig. R5c** (**Fig. 3a** in the updated manuscript and **Supplementary Fig. 12a** in the Supplementary Information), these dual-labeled MFlips all performed obvious membrane-fusion interaction with HeLa cells in the FBS-free medium. Notably, the DiD-labeled shell (red) predominantly localized on the CellMask-stained plasma membrane (cyan), while the YOYO-1-labeled plasmids (green) were dispersed throughout the cytoplasm. However, in a 10% FBS-containing medium, only DiD-AFMFlip$^{YOYO\text{-}1}$ continued to interact with cells *via* membrane fusion. In contrast, DiD-MFlip-a$^{YOYO\text{-}1}$ and DiD-MFlip-b$^{YOYO\text{-}1}$ exhibited overlapping red and green signals in the cytoplasm. Further validation of these observations was provided by fluorescence intensity line scanning analysis in **Fig. R5d** (the updated fluorescence analysis in **Fig. 3a**). This analysis confirmed the sustained fusion effect of AFMFlip and the involvement of endocytosis for MFlip-a and MFlip-b in FBS-containing media. Additionally, we employed a FRET-based assay to quantify the temporal membrane-fusion performance of AFMFlip, MFlip-a, and MFlip-b. As shown in **Fig. R5b** (**Supplementary Fig. 12b** in the updated Supplementary Information), AFMFlip, MFlip-a, and

MFlip-b exhibited similarly high fusion performance in the FBS-free medium. However, upon exposure to 10% FBS, only AFMFlip maintained high membrane-fusion efficiency, whereas the efficiencies of MFlip-a and MFlip-b significantly decreased. The time-dependent efficiency curves revealed that the maximum membrane-fusion efficiency was reached at 60 minutes for these liposomes, thereby justifying the choice of a 1-hour incubation period for the CLSM observation experiments.

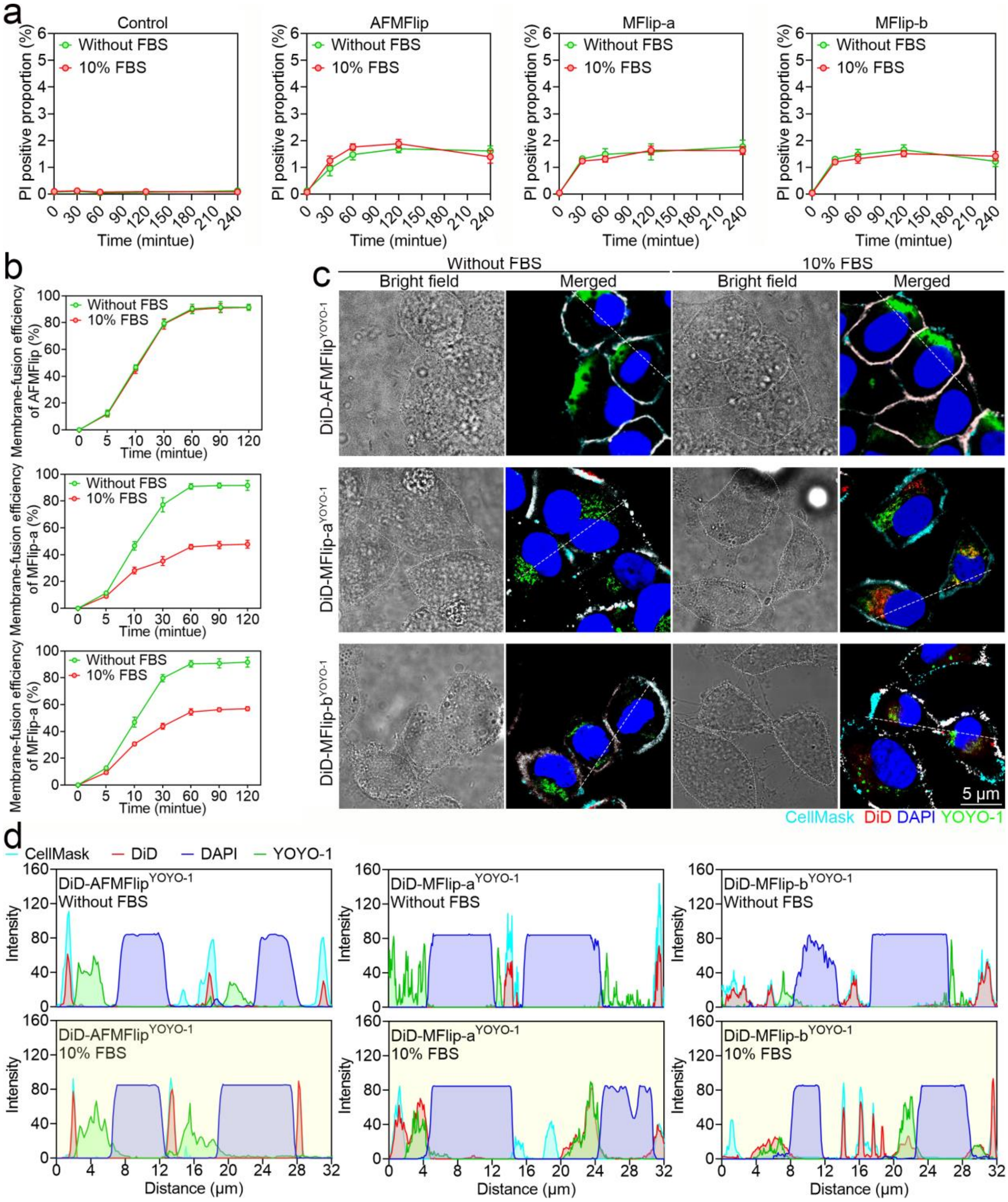

Fig. R5 | Membrane-fusion performance of AFMFlip, MFlip-a, and MFlip-b in the

medium with or without 10% FBS. a Quantitative analysis of propidium iodide (PI)-positive cells post-treatment with AFMFlip, MFlip-a, and MFlip-b in media without or with 10% FBS at 37 °C. **b** Time-dependent membrane-fusion performance of AFMFlip, MFlip-a, and MFlip-b based on FRET analysis after cell incubation without or with 10% FBS at 37 °C. **c** CLSM images of the cellular uptake of DiD-AFMFlip^{YOYO-1}, DiD-MFlip-a $^{YOYO-1}$, and DiD-MFlip-b $^{YOYO-1}$ in the medium with or without 10% FBS. The liposomes were labelled with DiD. The nuclei were stained with DAPI, and the cell membranes were labelled with CellMask. **d** Line scanning analysis along the white lines by ImageJ software.

For **Fig. 5e**, to mitigate potential interference from background autofluorescence, we conducted a repetition of the experiment using DiR-AFMFlips, DiR-MFlip-a, and DiR-MFlip-b, with the sole label of lipid layers by DiR (**Fig. R16**). Aligning with the observations in **Fig. 5e**, the DiR-AFMFlip group exhibited a notable fluorescence overlap between the DiR-marked lipid layers (red) and the phalloidin-stained cellular membranes (cyan). In contrast, the other two MFlips showed some DiR fluorescence within the cell interiors, suggesting a simultaneous occurrence of membrane fusion and endocytosis. These findings provide additional support to the observations and conclusions presented in **Fig. 5e**.

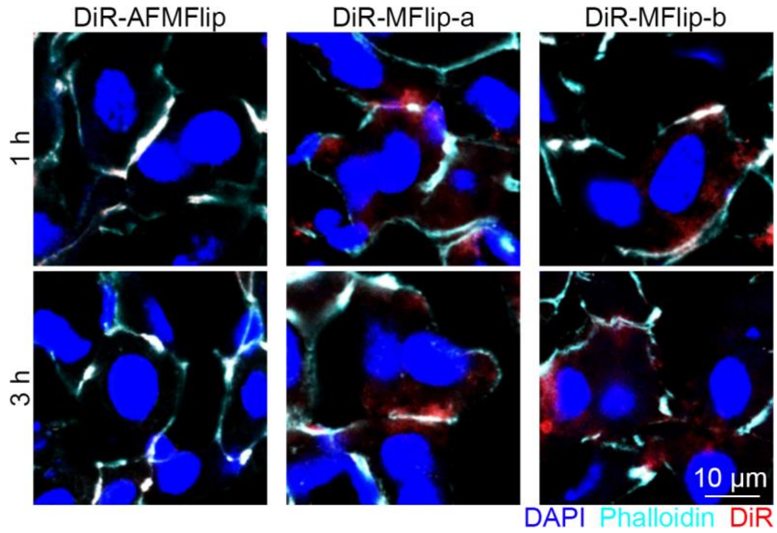

Fig. R16 | CLSM observation of the liver tissues from mice with the treatment of DiR-AFMFlip, DiR-MFlip-a, and DiR-MFlip-b *via* intravenous injection for 1 h and 3 h.

6. YOYO plasmid loading in liposomes did not involve any purification, how are the authors sure the signal in e.g. Fig.3a and corresponding flow cytometry data comes from released

plasmid rather than uptake of free plasmid? Information on loading efficiency, i.e. percentage of free plasmid should be included.

Response: Thank you very much for your meticulous review and helpful reminder. We apologize for the oversight in not initially detailing the purification step for plasmid-loaded nanoparticles before the *in vitro* experiments. Sincerely, we have included a comprehensive description of this process in the revised 'Methods - Synthesis of MFlips' section.

"Finally, the solution containing the plasmid-loaded nanoparticles underwent purification through centrifugation at 21000 g for 50 min at 4 °C. Subsequently, the precipitates were collected. To determine the encapsulation efficiency of plasmids within nanoparticles, the concentration of unencapsulated free plasmid in the supernatant of the solution after centrifugation was quantified using a NanoDrop 2000 spectrophotometer (Wilmington, DE, USA). The encapsulation efficiency was calculated using the following formula: EE (%) = (CT – CF)/CT × 100%, where EE represents the encapsulation efficiency, CT is the total concentration of plasmids, and CF denotes the concentration of unencapsulated, free plasmids in the supernatant."

Accordingly, we have included the plasmid loading efficiency data for AFMFlip, MFlip-a, and MFlip-b in **Fig. R17** (**Supplementary Fig. 11b** the updated Supplementary Information) and added the related description in the manuscript text. The results indicated that these nanoparticles showed excellent plasmid loading efficiency, reaching nearly 90%.

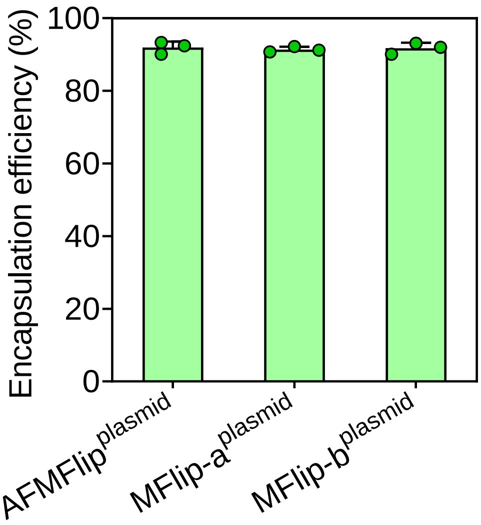

Fig. R17 | Plasmid encapsulation efficiencies of the plasmid-loaded AFMFlip, MFlip-a, and MFlip-b (n = 3).

7. Abstract: Before reading the paper, it was not clear to me what % threshold is referring to: "Consequently, AFMFlip can perform a steady membrane-fusion-mediated gene delivery in the serum-containing medium with a 38% concentration threshold, outclassing two traditional MFlips (4% and 6% respectively)". Please reword.

Response: Thank you very much for your thorough review and insightful comments. We have revised our description in the part of the abstract, and the detailed revision is as follows: "Consequently, AFMFlip demonstrates robust membrane-fusion-mediated gene delivery in the medium with up to 38% fetal bovine serum, outclassing two traditional MFlips effective at 4% and 6% concentrations."

8. PDI for DLS is typically not given as a percentage, DLS x-axis log-scale normally.

Response: Thank you very much for your meticulous review and helpful reminder. In accordance with your suggestions, we have included **Fig. R18** as the revised Supplementary **Fig. 2**. Additionally, we have updated the Supplementary Table to include the PDI values as you suggested (**Table R1**).

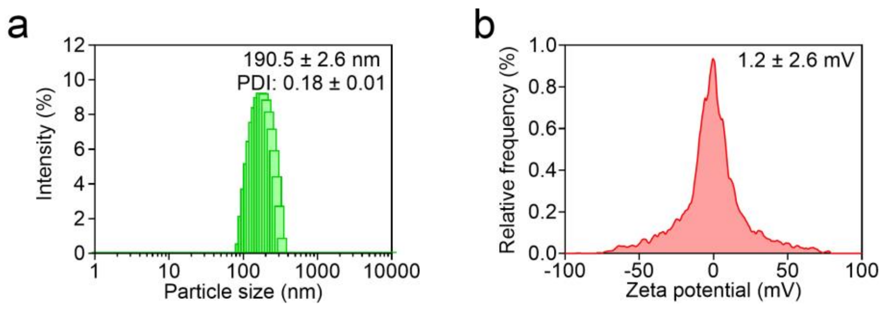

Fig. R18 | Size distribution and zeta potential of the optimized AFMFlip.

9. p14, DMPC or DOPC used in the formulations? Both are mentioned currently.

Response: Thank you for your thorough review and valuable reminder. We apologize for any confusion caused by our oversight and have corrected this error. Additionally, we have meticulously reviewed both the manuscript and the Supplementary Information to identify and correct any similar errors.

10. Number of experiments/repeats (N=XX, n=xx) need to be provided everywhere, e.g. for Fig. 2h, without showing dots in graph, not clear how many repeats were done here?

Response: Thank you very much for your attentive review and helpful reminder. We have carefully reviewed and updated our manuscript to include a specific number of experiments and repeats. These revisions have been clearly highlighted in red in the updated version for ease of identification.

11. Fig.3a; the cross-sections, especially for the bottom examples, could easily have been drawn differently in these figures, which would change the conclusion (there is clearly green within the cells for MFLips+10% FBS, the lines have just not been drawn through that area)

Response: Thank you for your detailed review and constructive comment. Accordingly, we have repeated CLSM observations, incorporating additional plasma membrane staining and bright field imaging to enhance visualization. The corresponding line scanning analysis has also been updated (**Fig. R6**, the updated **Fig. 3a**). Moreover, to more conclusively verify the intracellular transportation mechanisms, we have investigated the internalization pathways by membrane fusion and endocytic inhibitors (**Fig. 3c** and **Fig. 3e**). The findings from these figures align with and support the conclusions drawn in the updated **Fig. 3a**.

12. Fig.4; errors in caption regarding letter labelling

Response: Thank you very much for your meticulous review and thoughtful reminder. We greatly appreciate your attention to detail. We have taken the necessary steps to rectify the mistake and have conducted a thorough review of both the manuscript and Supplementary Information to address any similar errors. Your input has been invaluable, and we are grateful for your assistance in improving the quality of our work.

13. Fig.5d; quantitative information on TEM image analysis of a single particle per sample should be removed from the image (red lines) and in the text. I cannot see how the authors choose to put these lines and if doing quantification, it should be done on at least several dozens of particles per sample, not just on one.

Response: We sincerely appreciate your professional and constructive suggestions. After carefully considering your comments, we concur that the method used for quantification was not rational. Consequently, we have taken the necessary steps to improve the clarity of our work. Specifically, we have removed the red lines and eliminated the quantitative information from

the text. Additionally, acknowledging the difficulty in observing the protein corona on the liposome surface, we have incorporated TEM images (n=3) with enlarged views to facilitate better visualization. As illustrated in **Fig. R19**, the AFMFlip group displayed a thinner corona compared to the other two MFlips.Furthermore, recognizing the concern regarding the inadequacy of TEM images for providing precise quantitative information, we have included SDS-PAGE analysis and BCA analysis to address this issue. These analyses confirm our findings, demonstrating that AFMFlip exhibits an exceptional antifouling effect (**Fig. 5b, c**). Your feedback has been invaluable in enhancing the rigor and comprehensibility of our research.

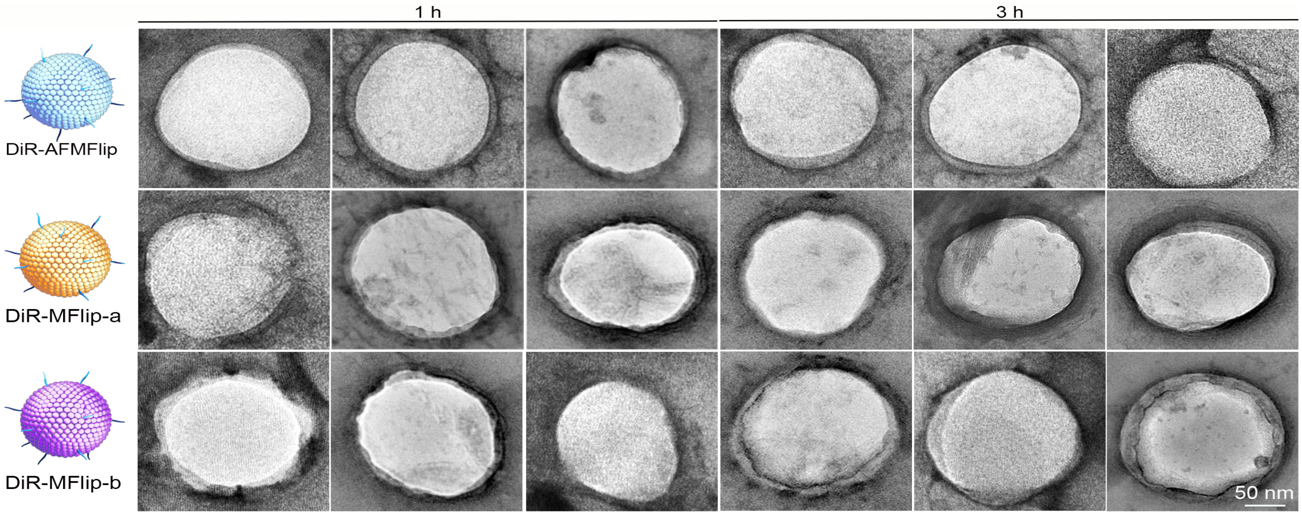

Fig. R19│TEM images of DiR-AFMFlip$^{YOYO\text{-}1}$, DiR-MFlip-a$^{YOYO\text{-}1}$, and DiR-MFlip-b$^{YOYO\text{-}1}$, recovered after intravenous injection for 1 h and 3 h (n = 3).

References

1 Mansfield, E., Ross, E. E. & Aspinwall, C. A. Preparation and characterization of cross-linked phospholipid bilayer capillary coatings for protein separations. *Anal. Chem.* **79**, 3135-3141, (2007).

2 van Weerd, J., Karperien, M. & Jonkheijm, P. Supported lipid bilayers for the generation of dynamic cell-material interfaces. *Adv. Healthcare Mater.* **4**, 2743-2779, (2015).

3 Liu, H. Y. *et al.* Rapid capture of cancer extracellular vesicles by lipid patch microarrays. *Adv. Mater.* **33**, 2008493, (2021).

4 Sut, T. N., Yoon, B. K., Jeon, W.-Y., Jackman, J. A. & Cho, N.-J. Supported lipid bilayer coatings: Fabrication, bioconjugation, and diagnostic applications. *Applied Materials Today* **25**, 101183, (2021).

5 Wang, Y.-F. *et al.* Transportation of aie-visualized nanoliposomes is dominated by the protein corona. *Natl. Sci. Rev.* **8**, nwab068, (2021).

6 Zheng, C. *et al.* Anti-phagocytosis-blocking repolarization-resistant membrane-fusogenic liposome (armful) for adoptive cell immunotherapy. *Sci. Adv.* **9**, eadh2413, (2023).

7 Zheng, C. *et al.* Membrane-fusion-mediated multiplex engineering of tumor cell surface glycans for enhanced nk cell therapy. *Adv. Mater.* **35**, e2206989, (2023).

8 Shi, C. *et al.* Targeting the activity of t cells by membrane surface redox regulation for cancer theranostics. *Nat. Nanotechnol.* **18**, 86-97, (2023).

9 Shao, Q. & Jiang, S. Molecular understanding and design of zwitterionic materials. *Adv. Mater.* **27**, 15-26, (2015).

10 Kim, B. *et al.* Immunogene therapy with fusogenic nanoparticles modulates macrophage response to staphylococcus aureus. *Nat. Commun.* **9**, 1969, (2018).

11 Kim, B. *et al.* Securing the payload, finding the cell, and avoiding the endosome: Peptide-targeted, fusogenic porous silicon nanoparticles for delivery of sirna. *Adv. Mater.* **31**, e1902952, (2019).

12 Kolašinac, R., Kleusch, C., Braun, T., Merkel, R. & Csiszár, A. Deciphering the functional composition of fusogenic liposomes. *Int. J. Mol. Sci.* **19**, 346, (2018).

13 Kolasinac, R. *et al.* Influence of environmental conditions on the fusion of cationic liposomes with living mammalian cells. *Nanomaterials (Basel)* **9**, (2019).

14 Lin, M. *et al.* A biomimetic approach for spatially-controlled cell membrane engineering using fusogenic spherical nucleic acid. *Angew. Chem., Int. Ed.* **61**, e202111647, (2021).

15 Cavalcanti, R. R. M., Lira, R. B. & Riske, K. A. Membrane fusion biophysical analysis of fusogenic liposomes. *Langmuir* **38**, 10430-10441, (2022).

16 Paez-Perez, M., Russell, I. A., Cicuta, P. & Di Michele, L. Modulating membrane fusion through the design of fusogenic DNA circuits and bilayer composition. *Soft Matter* **18**, 7035-7044, (2022).

17 Choi, W. *et al.* Reverse actuation of polyelectrolyte effect for in vivo antifouling. *ACS Nano* **15**, 6811-6828, (2021).

18 Chen, S. F., Zheng, J., Li, L. Y. & Jiang, S. Y. Strong resistance of phosphorylcholine self-assembled monolayers to protein adsorption: Insights into nonfouling properties of zwitterionic materials. *J. Am. Chem. Soc.* **127**, 14473-14478, (2005).

19 Liu, X. *et al.* Fusogenic reactive oxygen species triggered charge-reversal vector for effective gene delivery. *Adv. Mater.* **28**, 1743-1752, (2016).

20 Jin, Y. *et al.* A nanodisc-paved biobridge facilitates stem cell membrane fusogenicity for intracerebral shuttling and bystander effects. *Adv. Mater.*, e2302367, (2023).

21 Zou, Y., Ito, S., Fujiwara, M. & Komatsu, N. Probing the role of charged functional groups on nanoparticles grafted with polyglycerol in protein adsorption and cellular uptake. *Adv. Funct. Mater.* **32**, (2022).

22 Cheang, T.-y. *et al.* Calcium carbonate/caip6 nanocomposite particles as gene delivery vehicles for human vascular smooth muscle cells. *J. Mater. Chem.* **20**, 8050–8055, (2010).

23 Wei, W. *et al.* Preparation of hierarchical hollow caco3 particles and the application as anticancer drug carrier. *J. Am. Chem. Soc.* **130**, 15809, (2008).

24 Ou, L., Chen, H., Yuan, B. & Yang, K. Membrane-specific binding of 4 nm lipid nanoparticles mediated by an entropy-driven interaction mechanism. *ACS Nano* **16**, 18090-18100, (2022).

25 Wagner, A. M. *et al.* Ionic combisomes: A new class of biomimetic vesicles to fuse with life. *Adv. Sci.* **9**, e2200617, (2022).

26 Yang, J., Tu, J., Lamers, G. E. M., Olsthoorn, R. C. L. & Kros, A. Membrane fusion mediated intracellular delivery of lipid bilayer coated mesoporous silica nanoparticles. *Adv. Healthcare Mater.* **6**, 1700759, (2017).

27 Qiu, M. *et al.* Lipid nanoparticle-mediated codelivery of cas9 mrna and single-guide rna achieves liver-specific in vivo genome editing of angptl3. *Proc. Natl. Acad. Sci. U. S. A.* **118**, (2021).

28 Jin, Y. *et al.* Applications of nanobiomaterials in the therapy and imaging of acute liver failure. *Nanomicro Lett.* **13**, 25, (2020).

29 Bottger, R. *et al.* Lipid-based nanoparticle technologies for liver targeting. *Adv. Drug Deliv. Rev.* **154-155**, 79-101, (2020).

30 Maherani, B., Arab-Tehrany, E., Kheirolomoom, A., Cleymand, F. & Linder, M. Influence of lipid composition on physicochemical properties of nanoliposomes encapsulating natural dipeptide antioxidant l-carnosine. *Food Chem.* **134**, 632-640, (2012).

31 Deng, H. *et al.* Tumor microenvironment activated membrane fusogenic liposome with speedy antibody and doxorubicin delivery for synergistic treatment of metastatic tumors. *ACS Appl. Mater. Interfaces* **9**, 9315-9326, (2017).

32 Guo, P., Yang, J., Huang, J., Auguste, D. T. & Moses, M. A. Therapeutic genome editing of triple-negative breast tumors using a noncationic and deformable nanolipogel. *Proc. Natl. Acad. Sci. U. S. A.* **116**, 18295-18303, (2019).

33 Chen, Y., Wu, T., Xie, S., Bai, Y. & Xing, H. Orientation-controlled membrane anchoring of bioorthogonal catalysts on live cells via liposome fusion-based transport. *Sci. Adv.* **9**, eadg2583, (2023).

34 Martens, S. & McMahon, H. T. Mechanisms of membrane fusion: Disparate players and common principles. *Nat. Rev. Mol. Cell Bio.* **9**, 543-556, (2008).

35 Karatekin, E. & Rothman, J. E. Fusion of single proteoliposomes with planar, cushioned bilayers in microfluidic flow cells. *Nat. Protoc.* **7**, 903-920, (2012).

36 Allen, T. M. Liposomes. *Drugs* **54**, 8-14, (1997).

37 Gabizon, A., Goren, D., Cohen, R. & Barenholz, Y. Development of liposomal anthracyclines: From basics to clinical applications1this paper is based on a lecture presented at the 8th international symposium on recent advances in drug delivery systems (salt lake city, ut, USA, 1997).1. *J. Controlled Release* **53**, 275-279, (1998).

38 Tenchov, R., Bird, R., Curtze, A. E. & Zhou, Q. Lipid nanoparticles─from liposomes to mrna vaccine delivery, a landscape of research diversity and advancement. *ACS Nano* **15**, 16982-17015, (2021).

39 Evers, M. J. W. *et al.* State-of-the-art design and rapid-mixing production techniques of lipid nanoparticles for nucleic acid delivery. *Small Methods* **2**, 1700375, (2018).

40 Li, C. *et al.* Ionizable lipid-assisted efficient hepatic delivery of gene editing elements for oncotherapy. *Bioact. Mater.* **9**, 590-601, (2022).

41 Zhang, Y. *et al.* Close the cancer-immunity cycle by integrating lipid nanoparticle-mrna formulations and dendritic cell therapy. *Nat. Nanotechnol.*, (2023).

42 Ren, J. *et al.* Chemical and biophysical signatures of the protein corona in nanomedicine. *J. Am. Chem. Soc.* **144**, 9184-9205, (2022).

REVIEWER COMMENTS

Reviewer #1 (Remarks to the Author):

Revised manuscript has addressed all of my concerns and it is now ready for publication.

Reviewer #2 (Remarks to the Author):

"The results in Fig. R8d showed a considerably lower membrane-fusion efficiency for DMPC-lip compared to AFMFlip, both with and without 10% FBS. This indicated that DMPClip was less effective in achieving membrane fusion with cells. Collectively, we concluded that AFMFlip with a specific ratio of DOPC/DOTAP/DSPE-PEG (90/5/5) can not only facilitate a membrane-fusion process but also maintain this effect in the protein-rich media owing to the abundant zwitterionic components of DOPC on the surface."

Comment 1: DMPC is a zwitterionic lipid as DOPC. DMPC-lips show identical size, surface charges, and antifouling properties to AFMF-lips at the same ratio (90/5/5) but no membrane-fusion efficiency. Why could the membrane-fusion capacity of AFMF-lips be ascribed to the abundant zwitterionic groups of DOPC? The antifouling does not necessarily lead to the conversion of endocytosis to membrane-fusion-mediated cellular uptake. The role of the surface properties (e.g. antifouling capacity) and the inner structure in the membrane-fusion efficiency of various liposomes should be fair elucidated, in particular in the abstract and conclusion section.

"Notably, these liposomes predominantly accumulated in the liver tissue within 1 h after intravenous injection. Moreover, there was minimal retention of liposomes in the bloodstream during circulation. This observation implies that these liposomes quickly reach liver tissues, minimizing their exposure to blood cells."

Comment 2: As shown in Figure 2, AFMFlips were remarkably untaken by cells via membrane-fusion mechanism within 1 h. Does AFMFlip fuse with the membrane of vascular endothelial cells or red blood cells before approaching liver? This is important for the whole-body biodistribution of the AFMFlip after systemic administration.

Reviewer #3 (Remarks to the Author):

I thank the authors for a thorough revision of their manuscript, which has satisfied the concerns I have raised.

Response to Referees

Dear reviewers,

Thank you so much for your insightful comments on our manuscript entitled "An antifouling membrane-fusogenic liposome for effective intracellular delivery *in vivo*". According to your advice, we have meticulously checked and revised our manuscript. We are confident that these revisions have enriched the manuscript, aligning it with the standards required for publication in ***Nature Communications***. Below, we have detailed our responses to each of your comments. Once again, we extend our sincere gratitude for your valuable contributions to our work.

Response to Reviewer 1

Revised manuscript has addressed all of my concerns and it is now ready for publication.

Response: Thank you very much for your feedback on our revised manuscript. We are pleased to hear that it has successfully addressed all of your concerns. Your insights and suggestions have been invaluable in enhancing the quality and clarity of our work. We appreciate the time and effort you dedicated to reviewing our manuscript.

Response to Reviewer 3

I thank the authors for a thorough revision of their manuscript, which has satisfied the concerns I have raised.

Response: We are grateful for your acknowledgment of our efforts in revising the manuscript. Your comments and suggestions were instrumental in guiding our revisions, and we are pleased to learn that these adjustments have satisfied your concerns.

Response to Reviewer 2

"The results in Fig. R8d showed a considerably lower membrane-fusion efficiency for DMPC-lip compared to AFMFlip, both with and without 10% FBS. This indicated that DMPClip was less effective in achieving membrane fusion with cells. Collectively, we concluded that AFMFlip with a specific ratio of DOPC/DOTAP/DSPE-PEG (90/5/5) can not only facilitate a

membrane-fusion process but also maintain this effect in the protein-rich media owing to the abundant zwitterionic components of DOPC on the surface."

Comment 1: DMPC is a zwitterionic lipid as DOPC. DMPC-lips show identical size, surface charges, and antifouling properties to AFMF-lips at the same ratio (90/5/5) but no membrane-fusion efficiency. Why could the membrane-fusion capacity of AFMF-lips be ascribed to the abundant zwitterionic groups of DOPC? The antifouling does not necessarily lead to the conversion of endocytosis to membrane-fusion-mediated cellular uptake. The role of the surface properties (e.g. antifouling capacity) and the inner structure in the membrane-fusion efficiency of various liposomes should be fair elucidated, in particular in the abstract and conclusion section.

Response: Thank you very much for your insightful comment. The membrane-fusion capacity is influenced by both the physiochemical properties and the ratios of lipid components[1-5]. According to the previous studies, MFlips can be engineered using three lipid components: (1) a significant proportion of neutral lipid to enhance fluidity and facilitate the fusion; (2) cationic DOTAP for electrostatic attraction to the cell membrane; (3) DSPE-PEG to dehydrate the space between the nanoparticle lipid coatings and the plasma membrane[1,6]. In our study, we selected DOPC, a widely-used zwitterionic lipid renowned for its antifouling properties[7-10], in combination with DOTAP and DSPE-PEG. We optimized their ratio to 90/5/5 for effective membrane fusion. DOPC's distinctive features, such as its unsaturated lipid tails and extended alkyl chain, aid in modulating the lipid coating's fluidity and boosting its interaction with the plasma membrane[11-14]. This increased fluidity from DOPC enables our AFMFlip to use less DOTAP for efficient cellular interaction, thereby initiating fusion while maintaining a higher proportion of zwitterionic groups on its surface. Therefore, the specific physicochemical properties and optimized ratio of DOPC, DOTAP, and DSPE-PEG in our AFMFlip (not the abundant zwitterionic groups of DOPC) contribute significantly to its membrane-fusion efficacy. Interestingly, our experimentally identified AFMFlip exhibited a high concentration of zwitterionic components (90% DOPC), which enhances its ability to perform the membrane-fusion process in protein-rich environments.

In our previous correspondence, we regret any confusion caused by our statement regarding AFMFlip and its composition. To clarify, the sentence "Collectively, we concluded that AFMFlip with a specific ratio of DOPC/DOTAP/DSPE-PEG (90/5/5) can not only facilitate a membrane-fusion process but also maintain this effect in the protein-rich media owing to the abundant zwitterionic components of DOPC on the surface" should be revised to "Collectively,

we concluded that AFMFlip with a specific ratio of DOPC/DOTAP/DSPE-PEG (90/5/5) can not only facilitate a membrane-fusion process, but also maintain this effect in the protein-rich media owing to the abundant zwitterionic components of DOPC on the surface".

Additionally, we have carefully reviewed and revised the relevant sections of our manuscript to ensure a precise and coherent presentation of this concept. The key modifications include: "Leveraging the unique physicochemical characteristics and the optimized DOPC/DOTAP/DSPE-PEG ratio (90/5/5), AFMFlip effectively facilitates membrane fusion. Importantly, this fusion capacity is preserved in protein-rich conditions, attributed to the copious zwitterionic phosphorylcholine groups that inherently resist protein adsorption" and "The integration of zwitterionic DOPC on the surface enables AFMFlips to adsorb fewer proteins, alleviating corona formation. Consequently, AFMFlip with its specific DOPC/DOTAP/DSPE-PEG ratio (90/5/5) can not only facilitate a membrane-fusion-mediated intracellular delivery without lysosomal entrapment, but also sustains this functionality in the serum-enriched environment due to the abundant zwitterionic DOPC.".

"Notably, these liposomes predominantly accumulated in the liver tissue within 1 h after intravenous injection. Moreover, there was minimal retention of liposomes in the bloodstream during circulation. This observation implies that these liposomes quickly reach liver tissues, minimizing their exposure to blood cells."

Comment 2: As shown in Figure 2, AFMFlips were remarkably untaken by cells via membrane-fusion mechanism within 1 h. Does AFMFlip fuse with the membrane of vascular endothelial cells or red blood cells before approaching liver? This is important for the whole-body biodistribution of the AFMFlip after systemic administration.

Response: We appreciate your thorough review and insightful comments. It's well-documented that lipid-based nanoparticles, when administered intravenously, tend to accumulate predominantly in the liver shortly after entering systemic circulation, a phenomenon that has spurred numerous liver-targeted therapeutic applications[14-17]. In alignment with this, we have included **Fig. R1** (Fig. R10c, d in the previous response), which presents *ex vivo* imaging of major organs (heart, liver, spleen, lung, and kidney) and blood samples from mice post intravenous administration of DiR-labeled liposomes. The results unequivocally show that AFMFlips are characterized by a rapid and pronounced accumulation in liver tissue. This accumulation is substantially greater than in any other organ or blood.

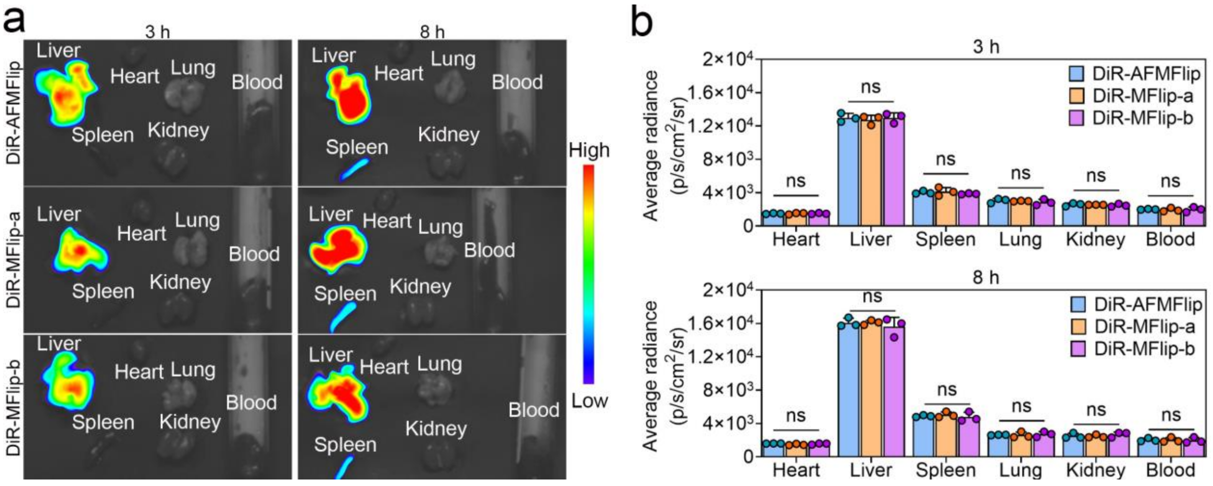

Fig. R1 | Representative *ex vivo* images of distribution (a) and corresponding semi-quantitative analysis in major organs (heart, liver, spleen, lung, and kidney) and blood (b) from mice at 3 h and 8 h post-injection of DiR-AFMFlip, DiR-MFlip-a, and DiR-MFlip-b (n = 3). ns: $p > 0.05$ (one-way ANOVA).

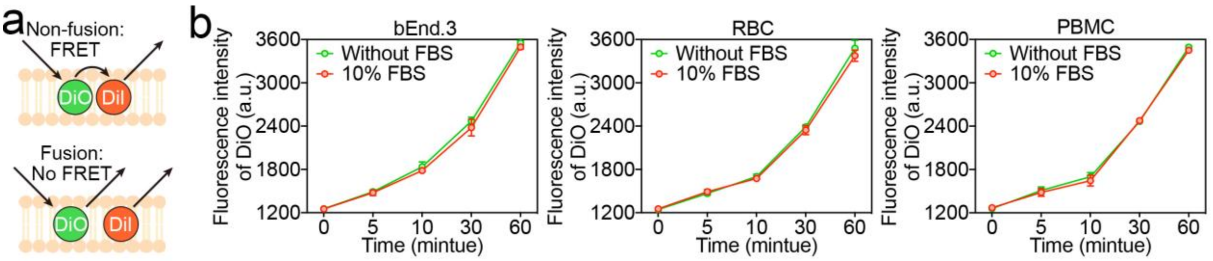

Fig. R2 | Membrane-fusion performance of AFMFlips with bEnd.3 vascular endothelial cells, red blood cells (RBCs), and peripheral blood mononuclear cells (PBMCs). a Schematic illustration of fluorescence resonance energy transfer (FRET)-based assay to evaluate the liposome-cell fusion. **b** Time-dependent DiO fluorescence changes based on FRET analysis after cell incubation without or with 10% FBS at 37 °C (n = 3).

Furthermore, to assess the membrane fusion capability of AFMFlip with blood cells, we quantitatively analyzed its fusion efficiency with the bEnd.3 mouse endothelial cell line, red blood cells (RBCs), and peripheral blood mononuclear cells (PBMCs) derived from mouse blood samples (C57BL/6 male mice, 3-5 weeks old). As shown in **Fig. R2**, these cells demonstrated a certain degree of membrane fusion with AFMFlip, observable both in the presence and absence of FBS, verifying the universal membrane-fusion effect of AFMFlip with cells. Consequently, following systemic administration of AFMFlip, a certain number of liposomes might inevitably fuse with blood cells or endothelial cells during circulation. However, the rapid accumulation of AFMFlips in liver tissues can mitigate exposure to blood

cells, potentially increasing the likelihood of membrane fusion with liver cells. Looking ahead, devising strategies to minimize non-specific membrane fusion will be a critical focus in refining the AFMFlip system.

In summary, our study focuses on addressing the significant challenge of retaining membrane-fusion effectiveness of MFlips *in vivo*. Future research endeavors will concentrate not only on preserving the *in vivo* membrane-fusion capabilities of AFMFlips but also on reducing unwanted biodistribution. Additionally, we will aim to refine targeting capabilities towards specific cell types critical for therapeutic interventions in various diseases.

Again, we sincerely appreciate all the constructive feedback and comments you provided, which have undoubtedly improved the quality of our work. Thank you for the time and attention you have dedicated to our manuscript.

References

1 Kim, B. *et al.* Immunogene therapy with fusogenic nanoparticles modulates macrophage response to Staphylococcus aureus. *Nat. Commun.* **9**, 1969, doi:10.1038/s41467-018-04390-7 (2018).

2 Lin, M. *et al.* A Biomimetic Approach for Spatially-Controlled Cell Membrane Engineering Using Fusogenic Spherical Nucleic Acid. *Angew. Chem., Int. Ed.* **61**, e202111647, doi:10.1002/anie.202111647 (2021).

3 Cavalcanti, R. R. M., Lira, R. B. & Riske, K. A. Membrane Fusion Biophysical Analysis of Fusogenic Liposomes. *Langmuir* **38**, 10430-10441, doi:10.1021/acs.langmuir.2c01169 (2022).

4 Paez-Perez, M., Russell, I. A., Cicuta, P. & Di Michele, L. Modulating membrane fusion through the design of fusogenic DNA circuits and bilayer composition. *Soft Matter* **18**, 7035-7044, doi:10.1039/d2sm00863g (2022).

5 Shi, C. *et al.* Targeting the activity of T cells by membrane surface redox regulation for cancer theranostics. *Nat. Nanotechnol.* **18**, 86-97, doi:10.1038/s41565-022-01261-7 (2023).

6 Kim, B. *et al.* Securing the Payload, Finding the Cell, and Avoiding the Endosome: Peptide-Targeted, Fusogenic Porous Silicon Nanoparticles for Delivery of siRNA. *Adv. Mater.* **31**, e1902952, doi:10.1002/adma.201902952 (2019).

7 Mansfield, E., Ross, E. E. & Aspinwall, C. A. Preparation and characterization of cross-linked phospholipid bilayer capillary coatings for protein separations. *Anal. Chem.* **79**, 3135-3141, doi:10.1021/ac0618829 (2007).

8 van Weerd, J., Karperien, M. & Jonkheijm, P. Supported Lipid Bilayers for the Generation of Dynamic Cell-Material Interfaces. *Adv. Healthcare Mater.* **4**, 2743-2779, doi:10.1002/adhm.201500398 (2015).

9 Liu, H. Y. *et al.* Rapid Capture of Cancer Extracellular Vesicles by Lipid Patch Microarrays. *Adv. Mater.* **33**, 2008493, doi:10.1002/adma.202008493 (2021).

10 Sut, T. N., Yoon, B. K., Jeon, W.-Y., Jackman, J. A. & Cho, N.-J. Supported lipid bilayer coatings: Fabrication, bioconjugation, and diagnostic applications. *Appl. Mater. Today* **25**, 101183, doi:10.1016/j.apmt.2021.101183 (2021).

11 Ou, L., Chen, H., Yuan, B. & Yang, K. Membrane-Specific Binding of 4 nm Lipid Nanoparticles Mediated by an Entropy-Driven Interaction Mechanism. *ACS Nano* **16**, 18090-18100, doi:10.1021/acsnano.2c04774 (2022).

12 Wagner, A. M. *et al.* Ionic Combisomes: A New Class of Biomimetic Vesicles to Fuse with Life. *Adv. Sci.* **9**, e2200617, doi:10.1002/advs.202200617 (2022).

13 Yang, J., Tu, J., Lamers, G. E. M., Olsthoorn, R. C. L. & Kros, A. Membrane Fusion Mediated Intracellular Delivery of Lipid Bilayer Coated Mesoporous Silica Nanoparticles. *Adv. Healthcare Mater.* **6**, 1700759, doi:10.1002/adhm.201700759 (2017).

14 Qiu, M. *et al.* Lipid nanoparticle-mediated codelivery of Cas9 mRNA and single-guide RNA achieves liver-specific in vivo genome editing of Angptl3. *Proc. Natl. Acad. Sci. U. S. A.* **118**, doi:10.1073/pnas.2020401118 (2021).

15 Li, J., Chen, C. & Xia, T. Understanding Nanomaterial-Liver Interactions to Facilitate the Development of Safer Nanoapplications. *Adv. Mater.* **34**, e2106456, doi:10.1002/adma.202106456 (2022).

16 Truong, B. *et al.* Lipid nanoparticle-targeted mRNA therapy as a treatment for the inherited metabolic liver disorder arginase deficiency. *Proc. Natl. Acad. Sci. U. S. A.* **116**, 21150-21159, doi:doi:10.1073/pnas.1906182116 (2019).

17 Liu, J. *et al.* Fast and Efficient CRISPR/Cas9 Genome Editing In Vivo Enabled by Bioreducible Lipid and Messenger RNA Nanoparticles. *Adv. Mater.* **31**, e1902575, doi:10.1002/adma.201902575 (2019).

REVIEWERS' COMMENTS

Reviewer #2 (Remarks to the Author):

The question has been fully addressed.
